# Oseltamivir aziridines are potent influenza neuraminidase inhibitors and imaging agents

**DOI:** 10.1073/pnas.2504045123

**Published:** 2026-03-23

**Authors:** Merijn B. L. Vriends, Elisha Moran, Martín Calvelo, Thomas Hansen, Isabelle B. Pickles, Xincheng Xin, Marieke Biezeno, Zachary W. B. Armstrong, Maria J. Ferraz, Lei Li, Alice Lilley, Ruth Harvey, Dmitri V. Filippov, Qinghua Liao, Sybrin P. Schröder, Gijsbert A. van der Marel, Marta Artola, Johannes M. F. G. Aerts, James N. Blaza, Jeroen D. C. Codée, Carme Rovira, Herman S. Overkleeft, Gideon J. Davies

**Affiliations:** ^a^Department of Bio-organic Synthesis, Leiden Institute of Chemistry, Leiden University, Leiden 2300 RA, The Netherlands; ^b^Department of Chemistry, University of York Heslington, York YO10 5DD, United Kingdom; ^c^Departament de Química Inorgaǹica i Orgaǹica (Seccióde Química Orgaǹica) and Institut de Química TeorÌica I Computacional, Universitat de Barcelona, Barcelona 08028, Spain; ^d^Fundació Catalana de Recerca i Estudis Avancats, Barcelona 08010, Spain; ^e^Department of Medical Biochemistry, Leiden Institute of Chemistry, Leiden University, Leiden 2300 RA, The Netherlands; ^f^Worldwide Influenza Centre, The Francis Crick Institute, London NW1 1AT, United Kingdom

**Keywords:** neuraminidase, influenza, cryoEM, chemical biology, carbohydrate-active enzyme

## Abstract

Influenza neuraminidase (NA) is a critical target for seasonal and pandemic antivirals, including the strains of current concern. Current treatments, such as Zanamivir and Oseltamivir, are limited by noncovalent binding and emerging resistance. We hypothesized that Oseltamivir aziridines would unite transition-state mimicry for tight binding, with aziridine-enabled covalent capture of the catalytic tyrosine, thereby supporting both therapy and activity-based quantification. Here, we present oseltamivir-based aziridines, inspired by cyclophellitol chemistry, that act as covalent inhibitors and activity-based probes via an *N*-acylaziridine warhead. Free-energy calculations, and NMR observations, indicate a ^4^H_5_ half-chair preference consistent with the NA transition state, and selected analogues inhibit multiple NA subtypes with low nanomolar binding constants. Diverse evidence establishes covalency: time-dependent inactivation, inhibitor washout, intact-mass shifts, MS/MS identification of a tyrosine adduct, and QM/MM reaction profiles, while cryoEM of N1 aligns with the proposed binding mode, revealing an elimination product. The inhibitors demonstrate formidable activity against diverse viral neuraminidases, including H5N1, and further enable imaging and quantification of active NA. With their dual therapeutic and diagnostic potential, these first-in-class inhibitors indeed benefit from transition state mimicry and covalency, and thus offer a powerful platform for antiviral development and neuraminidase imaging, addressing urgent global health needs in influenza treatment and prevention.

## Significance

Influenza remains a major global health threat. We introduce oseltamivir-based aziridines that unite transition-state mimicry for tight binding with aziridine-enabled covalent capture of the catalytic tyrosine. This dual function yields potent, mechanism-based neuraminidase inhibition and enables activity-based quantification of active enzyme directly in complex samples. Across N1, N2, and influenza B enzymes, selected compounds show high potency against diverse viral neuraminidases and in live virus replication assays. By combining a clinically grounded scaffold with a reactivity handle, these molecules bridge therapeutic and diagnostic needs and offer a practical platform for neuraminidase imaging and antiviral development.

Our current front-line antivirals for seasonal and pandemic influenza target the viral surface neuraminidase (NA) of the virus. Once a virus has gained entry through its hemagglutinin (HA), and successfully replicated, the neuraminidase enzyme catalyzes release of the new virions from the cell surface through cleavage of terminal sialic acids (the attachment point of HA) (1). Current antivirals such as Zanamivir (Relenza; inhaled medication) **1** and Oseltamivir (Tamiflu; oral tablet) **2**, as well as Peramivir (Rapivab; intravenous medication) and Laninamivir (Inavir; inhaled medication), inhibit the influenza neuraminidase in an active-center noncovalent manner, preventing virion release and reducing viral spread (2–5). Given the danger of pandemic influenza, the evolution of seasonal influenza strains and drug-resistant variants, it is no surprise that many research efforts, worldwide, are dedicated to discovering potent, novel, neuraminidase scaffolds as next-generation antivirals (6–13). Furthermore, alongside HA, neuraminidase is frequently a component of current human vaccine compositions. Global neuraminidase level is currently frequently assayed using enzyme activity (14). The ability to assay, specifically, active individual influenza NA in vaccines, and selectively detect this enzyme in other diverse biological samples, would be a key asset in diagnosing disease, offering superiority over global activity measurements for vaccine quality control (15–17).

**Fig. 1.**
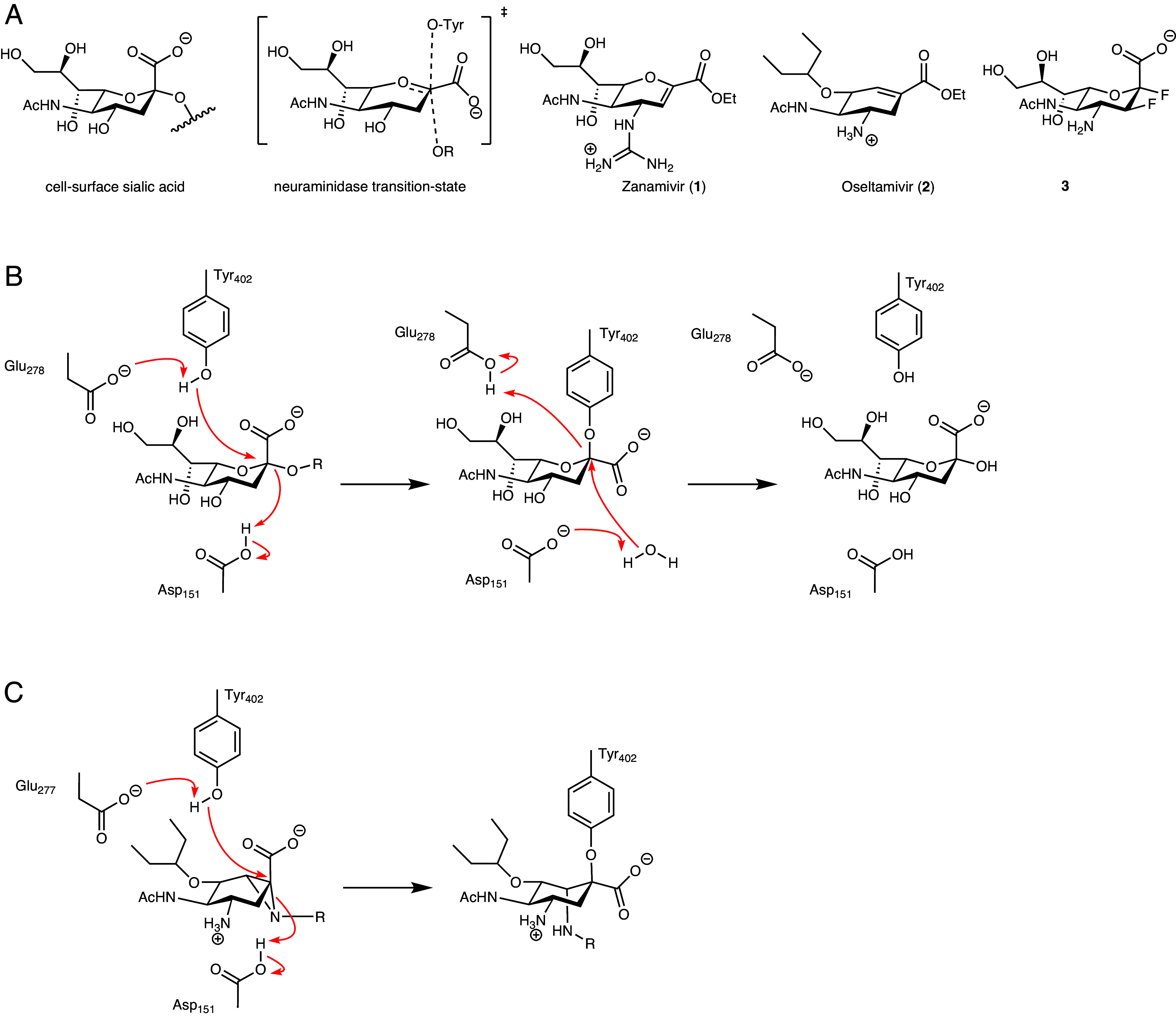
Structures of Influenza substrate and key antiviral therapeutics, mechanism of action of influenza neuraminidase with substrate and antiviral Oseltamivir. (*A*) Chemical structure of sialic acid, sialic acid–neuraminidase transition state, Zanamivir **1**, Oseltamivir **2** and 2,3 difluorosialic acid (“FeqAmDFSA” **3**). (*B*) Catalytic mechanism of influenza neuraminidase GH34 with substrate bearing a sialic acid occurs via a double displacement mechanism. (*C*) Enzymatic ring opening of cyclophellitol aziridine forming a covalent enzyme–inhibitor adduct.

Influenza viral neuraminidase catalyzes the hydrolysis of sialic acids with net retention of their anomeric configuration. Mechanistic studies have shown that this occurs via the formation and subsequent breakdown of a covalent tyrosyl-enzyme intermediate flanked by oxocarbenium-ion-like transition states (TSs), Fig. 1 (2, 18, 19). This mechanism has led to two disparate directions for enzyme inhibition. Mimicry of the transition-state ^4^H_5_ conformation is the fundamental basis for the tight but reversible binding of both Oseltamivir, and to a lesser extent Zanamivir; with both of these compounds developed to harness a negative patch in the influenza neuraminidase active center pocket (3, 20). This design feature also provides specificity over human neuraminidases. In a second approach, Withers and others have developed and exploited covalent inhibitors. These compounds do not bind initially as TS mimics, but instead exploit the nucleophilic active center tyrosine of the catalytic cycle trapping a long-lived covalent adduct through the use of 3-fluoro substituents and thus inhibiting the viral neuraminidase (2).

Inspired by the Withers 2,3-difluoro sialic-acid-derived covalent inactivators (including **“FeqAmDFSA” 3**) and by the C4 substituent pattern that underlies oseltamivir’s potency and selectivity, we sought to combine these principles in one chemotype. We therefore replaced the oseltamivir alkene with a suitably configured, substituted aziridine, leveraging cyclophellitol chemistry to enable covalent adduct capture. In addition to serving as an electrophile for the catalytic Tyr, we hypothesized that the aziridine ring might adopt TS-like (^4^H_5_) half-chair conformations potentially providing transition-state mimicry of the NA oxocarbenium-ion-like transition state. Together, we believed these features would yield mechanism-based, covalent, and irreversible NA inhibitors, with an aziridine nitrogen functionality that could subsequently be exploited for an activity-based probe (ABP).

Although never previously tested for a phenolic nucleophile, retaining glycosidase inhibitors and probes have in the past been designed successfully based on the natural product, cyclophellitol, a glucose mimetic cyclitol featuring an epoxide spanning the two carbons emulating C1 and O5 in the natural substrate (21, 22). Cyclophellitol adopts a conformation that is highly similar to the conformation the β-glucoside adopts in the transition state of the hydrolysis reaction, and enzyme active site protonation of the epoxide with concomitant nucleophilic displacement by the active site nucleophile yields a covalent and irreversible enzyme inhibitor adduct. Substituting the epoxide in cyclophellitol for aziridine yields equally effective enzyme inactivators and alkylating the aziridine then provided an entry into activity-based retaining β-glucosidase ABPs. Motivated by these retaining-enzyme precedents, our objective was to create oseltamivir-based aziridines that i) mimic the neuraminidase transition state to achieve tight, selective binding and ii) subsequently form a covalent adduct with the catalytic tyrosine, enabling durable inhibition and activity-based quantification. We first establish transition-state mimicry and potency, then provide convergent biochemical, MS/MS, and QM/MM evidence for covalency, followed by structural analysis and applications to vaccine NA quantification and live-virus assays.

We chose Oseltamivir as our starting scaffold as it most closely resembles the ^4^H_5_ half chair transition state and was itself designed to achieve selectivity for influenza neuraminidases over human ones. Installing an aziridine will influence PKPD properties, possibly impacting oral availability, which is an advantageous feature of Oseltamivir. However, we pose that PKPD properties may be tuned to emulate those of the parent compound, simply by varying the N-alkyl/acyl functionality in future generations without touching the Oseltamivir-inherent ring appendages which were optimized through extensive medicinal chemistry efforts.

Here, we show that Oseltamivir-inspired aziridines are indeed tight binding, specific and covalent inactivators of viral neuraminidases. Computational analyses of their conformational landscape show that they are indeed transition-state mimics reflected in low nanomolar binding constants on different viral neuraminidases. Time-dependent inhibition, resistance to washing, and mass spectrometry reveal that the *N*-acylaziridines act covalently and are active center tyrosine directed. Although cryoEM analysis revealed an elimination product, consistent with past structural work on neuraminidases, it allowed mapping of the active center interactions of these new compounds. Furthermore, the compounds not only block viral replication in infected cells at concentrations at least equal to Oseltamivir, across different viral strains, but the aziridine nitrogen indeed allows activity-based profiling of active viral neuraminidases in complex samples.

## Results

### Synthesis of Oseltamivir Aziridines.

**Scheme 1.**
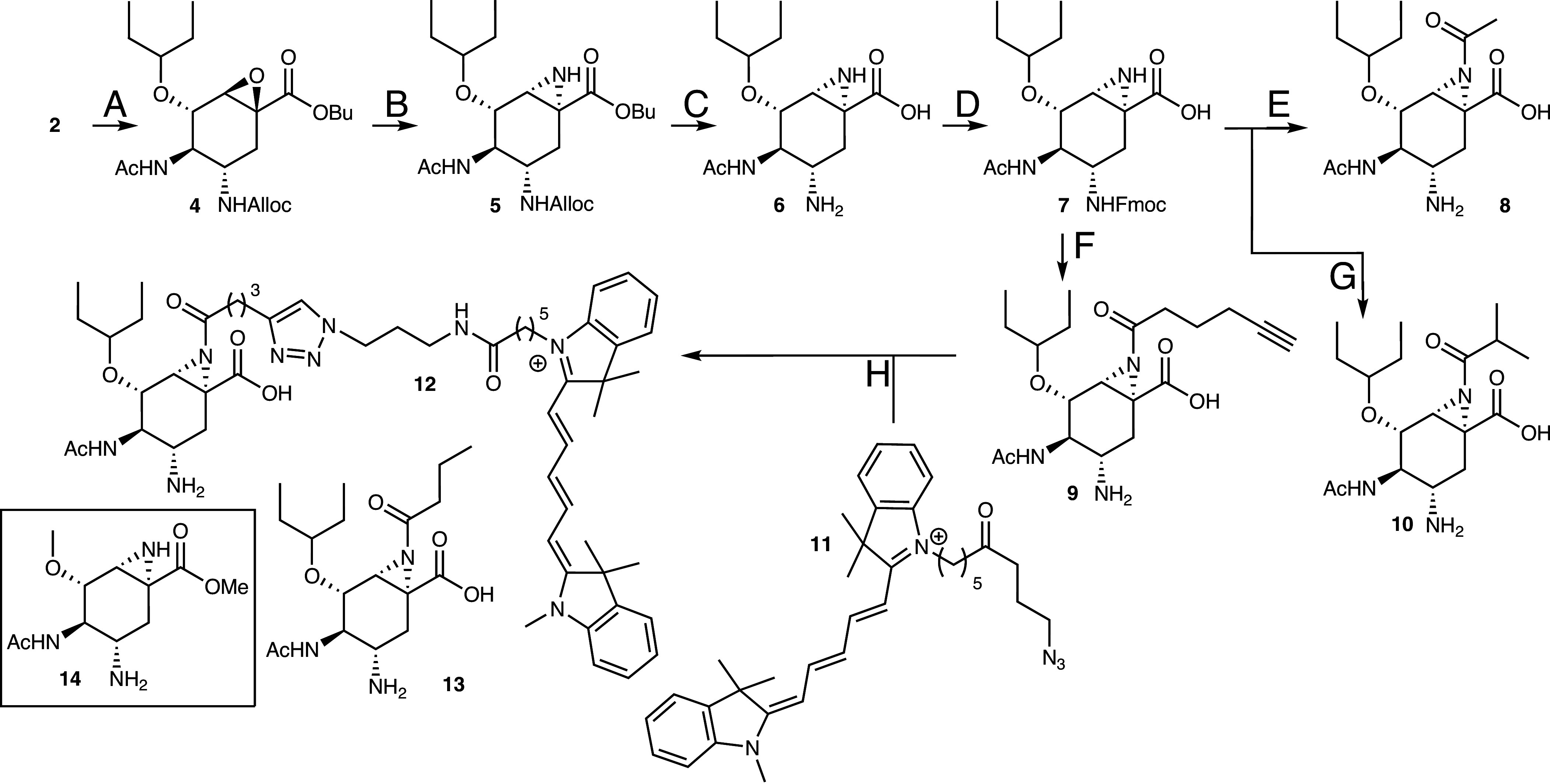
Structure and synthesis of Oseltamivir aziridine inhibitors and probes. Reagents and conditions. (*A*) (*i*) Alloc-Cl, NaHCO_3_, THF, H_2_O, 93%; (*ii*) *^t^*BuOOH, *n*-BuLi, THF, −78 °C to rt, 38%. (*B*) (*i*) NaN_3_, NH_4_Cl, EtOH, H_2_O, reflux; (*ii*) MsCl, Et_3_N, pyridine, 0 °C to rt; (*iii*) Bu_3_P, THF, then Et_3_N, H_2_O, 32% (three steps). (*C*) (*i*) NaOH, MeOH, dioxane, H_2_O; (*ii*) Pd(Ph_3_)_4_, *N*,*N*-dimethylbarbituric acid, CH_2_Cl_2_, MeOH, 77% (two steps). (*D*) FmocOSu, NaHCO_3_, THF, H_2_O; 48%. (*E*) (*i*) Ac_2_O, pyridine; (*ii*) DBU, DMF, 21% (two steps). (*F*) (*i*) 1-hexynoic anhydride, pyridine, 75%; (*ii*) DBU, DMF, 83%. (*G*) (*i*) isobutyric anhydride, pyridine, 36%; (*ii*) DBU, DMF, 83%. (*H*) CuSO_4_, sodium ascorbate, DMF, 73%. Compound **14** (boxed) is a simplified Oseltamivir aziridine used for computational energy landscape calculations (see below).

Perusal of the molecular structure of Oseltamivir **2** (Fig. 1) reveals an entry into influenza neuraminidase-targeting cyclophellitol inhibitors and probes; by transforming the double bond in the α-β-unsaturated system into an aziridine, which is then acylated or alkylated, Scheme 1. Oseltamivir **2**, which is commercially available in gram quantities, also provides a straightforward starting material to the target compounds, obviating the need for a de novo synthesis scheme. Thus, protection of the free amine as the allyl carbamate under Schotten-Baumann conditions, followed by nucleophilic epoxidation of the α,β-unsaturated ester moiety using *tert*-butylperoxide and *n*-butyllithium, yielded epoxide **4**. In this process and due to the use of *n*BuLi, the ethyl ester transesterified to the butyl ester (23). The epoxidation proceeded stereoselectively to yield β-configured cyclitol epoxide **4**. Treatment of **4** with sodium azide provided a mixture of vicinal-azido alcohols that were transformed into aziridine **5** as the single diastereomer (with the desired pseudo-anomerical configuration) by first mesylation of the secondary or tertiary alcohol, followed by reaction with tributylphosphine (formation of phosphazenes which then performed intramolecular nucleophilic attack on the mesylates) and final addition of water and base to break up the intermediate N-P bond. Ester hydrolysis followed by palladium-catalyzed Alloc removal afforded Oseltamivir aziridine **6** as our first synthesis target. Reprotection of the free primary amine in **6** as the Fmoc carbamate (**6** to **7**) was followed by aziridine-*N*-acylation (**7** to **10**) and then condensation with the appropriate anhydride using pyridine as a base. DBU was used to deprotect the Fmoc moiety. Alternatively, a Cbz-based protection scheme was used to prepare butyryl aziridine **13** (for synthesis see SI). Copper-(I)-catalyzed [2 + 3] alkyne-azide “click” cyclization of aziridine alkyne **9** with Cy5-azide **11** afforded compound **12** and completed the synthesis of the four putative mechanism-based neuraminidase inhibitors **6**, **8** to **10,** and **13**, as well as potential activity-based neuraminidase probe **12**.

### Oseltamivir-Inspired Aziridines Adopt Transition-State Mimicking Conformations and Are Tight Binding Influenza Neuraminidase Inhibitors.

**Fig. 2.**
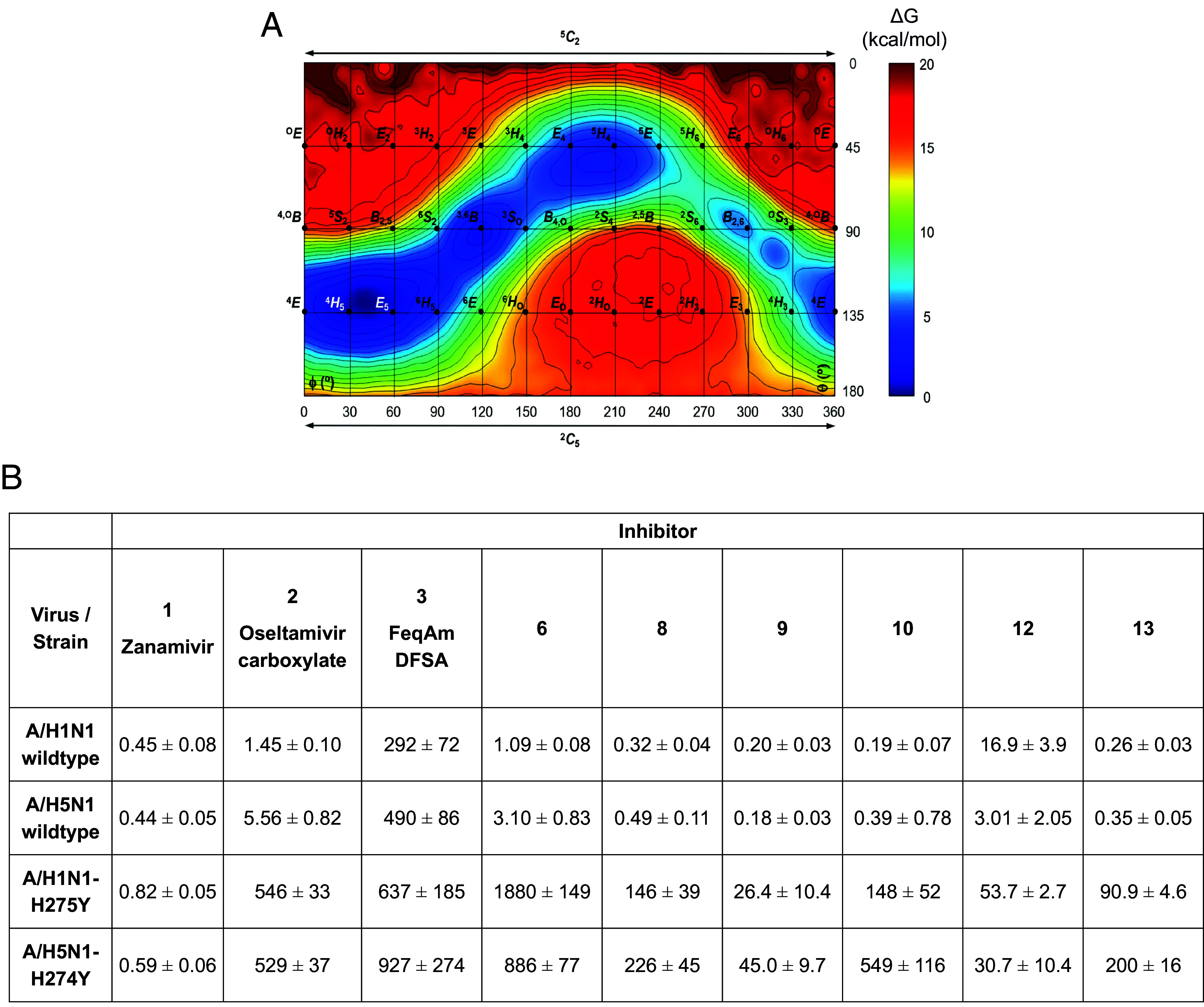
Conformational preferences of Oseltamivir aziridines and their activity. (*A*) Conformational FEL of model Oseltamivir aziridine **14** The x- and y-axes correspond to the *φ* and *θ* Cremer−Pople puckering coordinates (°), respectively. Isolines are 1 kcal mol^−1^. (*B*) Apparent IC_50_ values (nM) for in vitro inhibition of H1N1 (A/CA/04/2009) and H5N1 (A/Anhui/1/2005) influenza A neuraminidases, as well as Oseltamivir-resistant mutants (H1N1-H275Y and H5N1-H274Y). Reported values are mean ± SD from two technical triplicates. IC_50_ values were determined using MUNANA as substrate following a 30-min preincubation of the overexpression lysate (at 0.6 to 1 U/mL) with varying concentrations of inhibitor. IC_50_ graphs are shown in *SI Appendix*, Fig. S3.

We first sought to test the hypothesis that the aziridine ring would impose a conformational preference for transition-state mimicking half chair conformations and thus yield (irrespective of subsequent covalency) tight binding inhibitors. We therefore sought to understand the conformational preferences of a simplified (to remove rotatable side chains) Oseltamivir aziridine **14** (Scheme 1, *Inset*) through the calculation of its conformational free energy landscape (FEL). We computed the FEL for simplified-aziridine **14** with respect to the Cremer–Pople ring puckering coordinates (*θ* and *φ*) by *ab initio* metadynamics (see Computational Methods in the SI for more details; Fig. 2*A* and *SI Appendix*, Fig. S1). The FEL clearly reveals the ^4^H_5_ conformation (numbering based on sialic acid nomenclature) to be the favored one, in vacuo, for both molecular structures (with the flipped ^5^H_4_ form as another local energy minimum). 2D NMR analysis experimentally confirms the ^4^H_5_ conformation (*SI Appendix*, Fig. S2) through analysis of the relevant ^3^J_HH_-couplings. The ^4^H_5_ half chair is indeed the expected transition state conformation for the neuraminidase-catalyzed reaction. In fact, mimicry of this conformation [as reflected in 3D structures of neuraminidase–Oseltamivir complexes, in which the ligand indeed has ^4^H_5_ conformation, for example (24)], contributes to the tight-binding of Oseltamivir itself. Armed with the confidence of a favored transition-state mimicking conformation we next sought to determine basic inhibition parameters on a panel of different neuraminidases.

We determined the inhibition parameters for the panel of inhibitors and relevant controls. Kinetics compared oseltamivir aziridines **6**, **8** to **10**, **12,** and **13** with the mechanism-based inhibitor 2,3-difluoroneuraminic acid **3** (Fig. 1) which is known to form a stable covalent glycosyl-intermediate adduct (2), alongside Oseltamivir carboxylate, the active form of Oseltamivir **2**, as well as another clinical anti-influenza drug, Zanamivir **1** (Fig. 1). To cover a range of clinically relevant influenza strains, we selected H1N1 and H5N1 influenza A neuraminidases, as well as two Oseltamivir-resistant mutants (H1N1-H275Y and H5N1-H274Y). We supplemented these with influenza A H3N2 and influenza B neuraminidases. Inhibition constants, including half-maximal inhibitory concentration (IC_50_) values, were assessed in a fluorogenic substrate assay with 2-(4-methylumbelliferyl)-α-D-*N*-acetylneuraminic acid (MUNANA) as the substrate (Fig. 2*B* and *SI Appendix*, Table S1 and Figs. S3 and S4).

Inhibition data show that Oseltamivir aziridine **6** (low nM apparent IC_50_ values and subnanomolar *K*_I_ values) and *N*-acylaziridines **8** to **10** and **13** (subnanomolar apparent IC_50_ and *K*_I_ values) inhibit the “wild-type” H1N1 and H5N1 enzymes on a par with Zanamivir **1** (the clinical anti-influenza drug, Relenza; subnanomolar apparent IC_50_ and *K*_I_ values) and slightly more effective than the parent compound, Oseltamivir carboxylate (low nM apparent IC_50_ values and subnanomolar *K*_I_ values). In these assays, the compounds are considerably more effective than 2,3-difluoroneuraminic acid **3** (mid-high nM apparent IC_50_ and *K*_I_ values) based on data reproduced here and the original data, but we note that other analogues of the 2,3-difluoroneuraminic acids were far better inhibitors in the original paper (5 nM IC_50_ values for the very best) (2). Consistent with the literature on Oseltamivir-resistant strains, Zanamivir **1** blocks the activity of both mutants equally effective as the wild-type enzymes, whereas Oseltamivir carboxylate activity is significantly diminished on the resistant variants. These results, which are supported by literature data, are matched by Oseltamivir aziridine **6**, which inhibits the two mutants less potently. Three of the *N*-acyl aziridines (**8**, **10,** and **13**), which, though modest in comparison with Zanamivir **1**, perform better than Oseltamivir itself on the resistant enzyme variants. Interestingly, alkyne-tagged aziridine **9** and Cy5-tagged ABP **12** only lose a small fraction of their potency against the mutants, relative to the other compounds. All compounds retain nanomolar activity relative to the parent compound against the influenza A H3N2 neuraminidase. However, against the influenza B neuraminidase tested, the *N*-acyl aziridines (**8** to **10** and **13**) are more potent than Oseltamivir carboxylate **2** and unsubstituted aziridine **6** (*SI Appendix*, Table S1). Given the potent inhibition, we next assessed specificity over human neuraminidases.

Our rationale for use of the Oseltamivir scaffold included the poor binding of this to human enzymes, IC_50_ values of 2713, 17, and 487 μM for Oseltamivir carboxylate against human Neu1, 2, and 4 respectively have been reported (25). As expected, weak inhibition is observed with compounds **8** and **12** at concentrations higher than 100 μM using human Neu2, *SI Appendix*, Fig. S4. Having demonstrated specific tight-binding inhibition of the aziridines, we next sought to probe their mode of action, in particular whether the compounds bind covalently to the unusual phenolic nucleophile of the active center tyrosine.

### Covalent Binding of Oseltamivir Aziridine.

Given our hypothesis that Oseltamivir (acyl) aziridines should also benefit from subsequent covalent inactivation, enhanced by initial tighter binding, we first sought to study the feasibility of that reaction, in silico using QM/MM simulations.

Starting from an X-ray structure of a N294S variant of the N1 enzyme crystallized with Oseltamivir **2** (PDB ID: 3CL2) (24), we reconstructed the structure of the wild-type enzyme in complex with **8** (See Computational Methods in SI) and carried out classical molecular dynamics (MD) simulations. The acyl substituent of **8** was modeled in its most favored *exo* conformation (determined both computationally, where the *exo* conformation is 7.9 kcal/mol more stable, so represents >99.999% in solution, and by NMR, *SI Appendix*, Fig. S2*B*). The ligand accommodated well in the active center, without disturbing the structural integrity of the enzyme (*SI Appendix*, Fig. S5 *A* and *B*). The distance between the proton of the carboxylic acid group of Asp151 and the N of the aziridine group fluctuates significantly (*SI Appendix*, Fig. S5*C*), indicating a weak hydrogen bond interaction. In contrast, the distances between atoms of the catalytic residues (Glu278 and Tyr402) and the ligand remain constant. In particular, Tyr402 adopts a well-suited orientation for nucleophilic attack on the anomeric carbon of **8**.

We subsequently modeled the potential formation of the covalent adduct between Tyr and **8** by QM/MM MD calculations combined with the on-the-fly probability enhanced sampling (OPES) (26) method (QM/MM OPES). Using a frame from the MD simulation, we employed two collective variables (CVs) to drive the system from reactants to products. The first CV involves the deprotonation Tyr402 and its nucleophilic attack (d_O···H-Tyr402_ – d_H-Tyr402···COO-Glu278_ – d_O-Tyr402···C2_). The second CV describes the protonation and ring opening of the aziridine (d_N···C2_ + d_COO···H-Asp151_ – d_H-Asp151···N_).

**Fig. 3.**
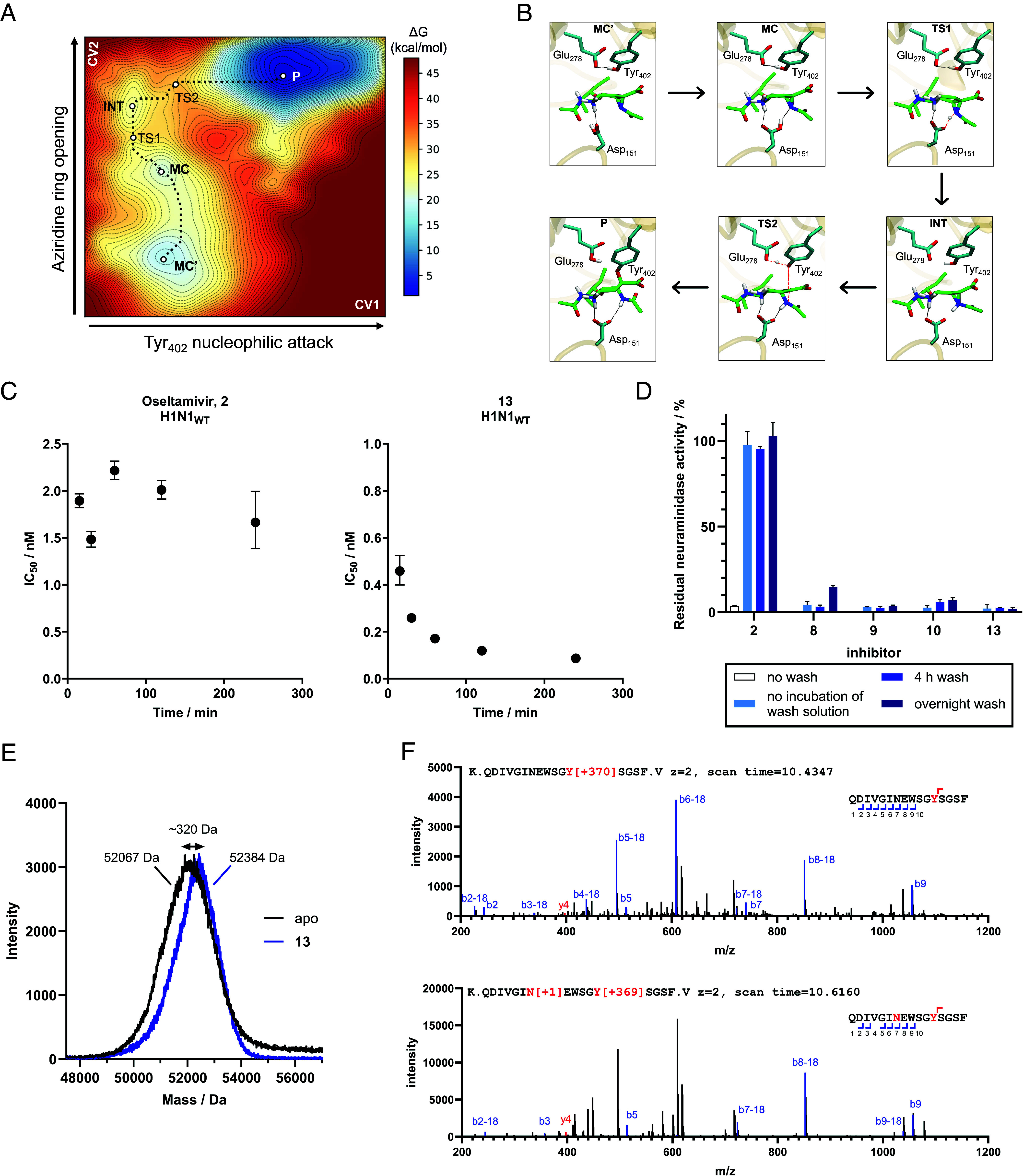
Evidence of covalency of the acyl-aziridines. (*A*) Free energy landscape and (*B*) representative structures of the main states observed in the formation of the covalent adduct from Oseltamivir *N*-acetylaziridine **8** and neuraminidase. (*C*) Time-dependency differences in IC_50_ values for noncovalent parent compound Oseltamivir **2** and Oseltamivir-aziridine **13**. IC_50_ curves are shown in *SI Appendix*, Fig. S6. (*D*) Acyl-aziridines cannot be “washed off” the neuraminidase, whereas Oseltamivir carboxylate 2 can be: H5N1 neuraminidase lysate was incubated with compounds **2**, **8**, **9**, **10**, and **13** (10 μM), and subsequently incubated with Concanavalin A (ConA) beads. These beads bound to H5N1 neuraminidase were then washed for 4 or 24 h to remove noncovalent bound compounds and MUNANA was then added to measure residual activity. Normalized residual activity is plotted as a mean of 3 repeats ± SD. (*E*) Intact mass spectrum of H1N1 neuraminidase before and after reaction with compound 13, showing the expected mass shift. (*F*) Example mass spectra of neuraminidase peptides covalently bound to compound **13** at Tyr402. Modification at residue N is deamidation.

Analysis of the FEL of the reaction (Fig. 3*A*) reveals that a covalent species forms with a low energy barrier (12 kcal/mol). The reaction coordinate shows two distinct stable configurations of the starting complex (two minima in the reactant region; MC’ and MC), dependent on the orientation of the likely catalytic acid (protonated) Asp151. In one configuration, Asp151 forms a hydrogen bond with the carbonyl group of the *N*-acetyl moiety of the C5 of ligand **8** (MC’), while in the other, it forms the catalytically required hydrogen bond with the nitrogen of the aziridine (MC). The two minima are energetically similar, within 2 kcal/mol of each other, consistent with the weak interaction between Asp151 and the nitrogen of the aziridine group observed in the classical MD.

The reaction coordinate, defined by the evolution of the catalytic distances along the minimum energy path (Fig. 3*B* and *SI Appendix*, Fig. S5*D*), indicates that the reaction initiates with the protonation of the aziridine *via* a first transition state (TS1) with a small energy barrier (8.7 kcal/mol relative to the global minimum, MC’). This in turn leads to the formation of an intermediate (INT, in which the nitrogen is protonated, but the aziridine ring has not yet opened), which is 4.8 kcal/mol less stable than MC’. The formation of this intermediate was corroborated by separate unbiased QM/MM MD simulations. The reaction proceeds with the opening of the aziridine ring, which is concurrent with nucleophilic attack by Tyr402 and leads to the formation of the covalent adduct, via a second transition state (TS2). Considering the energetic cost of protonating Asp151 (p*K*_a_ 5.0), the reaction free energy barrier is 15 kcal/mol, indicating that the formation of the covalent adduct is a feasible and favored process. The overall process is exothermic, underscoring the stability of the covalent adduct relative to the starting complex for the acyl aziridine. During these simulations, we noticed a small degree of elimination from the final product, as known to happen to the natural substrate and covalent adducts. This will be discussed more in light of cryoEM analyses, later.

Given that computation demonstrates covalency, we sought to use experimental approaches to analyze covalent inhibition. Covalent inhibition is classically determined in three ways: time dependence of inhibition, resistance of inhibition to washing, and mass spectrometry, all were deployed in this case. Covalent inhibition was initially assessed through time dependence of IC_50_ values (Fig. 3*C* and *SI Appendix*, Fig. S5) and subsequent inactivation kinetics for those compounds (all but **6**) that were covalent. The apparent second-order inactivation kinetics, *k*_inact_ and *K*_i_ were determined through continuous readout of MU release from MUNANA as a function of inhibitor concentration (*SI Appendix*, Fig. S7 and Table S2). Most of the compounds show tighter *K*_i_ than the reference compound FeqAmDFSA **3** [with the caveat that **3** is far from the best of the Withers’ 3-fluoro inhibitors originally analyzed (2)], leading to better *k*_inact_/*K*_i_ albeit with slower inactivation kinetics. This may reflect the much tighter binding afforded by the conformational mimicry of the aziridines compared to the sugar fluoride **3**, but poorer reactivity due to the challenges for suitable aziridine protonation (discussed above). Intriguingly, in this light, unsubstituted aziridine **6** did not show time-dependence on wild type neuraminidases (this is discussed further below).

Following this, H1N1 from lysates was immobilized on ConA beads and reacted with the acyl aziridines (**8** to **10** and **13**) or Oseltamivir carboxylate **2**, before “washing” of the beads to remove noncovalently bound compound. Residual activity of the ConA-bound NA was measured using MUNANA, with all acylaziridines retaining almost complete depletion of activity, whereas parent compound **2** could be completely “washed out” (Fig. 3*D*).

Final demonstration of covalency, using mass spectrometry, was performed using acyl aziridine **13**. Although we found that viral neuraminidases are extremely challenging for mass spectrometry, MALDI-TOF analysis showed an expected mass increase upon incubation of N1 (used for cryoEM studies) with **13** (Fig. 3*E*). Subsequently, we performed in-solution digestion with trypsin and chymotrypsin of N1 bound to **13** and analyzed the resultant peptides by MS/MS using a timsToF HT spectrometer. Analysis using Byonic software revealed Tyr402 had reacted specifically with **13** (Fig. 3*F*).

Oddly, in our assays unsubstituted aziridine **6** did not show classic features of covalency on wild type enzymes, yet one would certainly expect covalent reactivity. QM/MM analyses of its reaction coordinate indeed showed that formation of the covalent adduct has essentially the same free energy barrier as the acyl aziridines, consistent with covalency, but in this case the products are less stable than the reactants and revert to the starting material (*SI Appendix*, Fig. S5*E*). While this feels counterintuitive, computation is reflected in the experimental data for this compound.

In light of tight and specific binding and covalency of the acyl aziridines, we next sought to determine the 3D structure of N1-acyl aziridine complexes.

### 3-D Structure of N1 Neuraminidase With Acyl Aziridines.

**Fig. 4.**
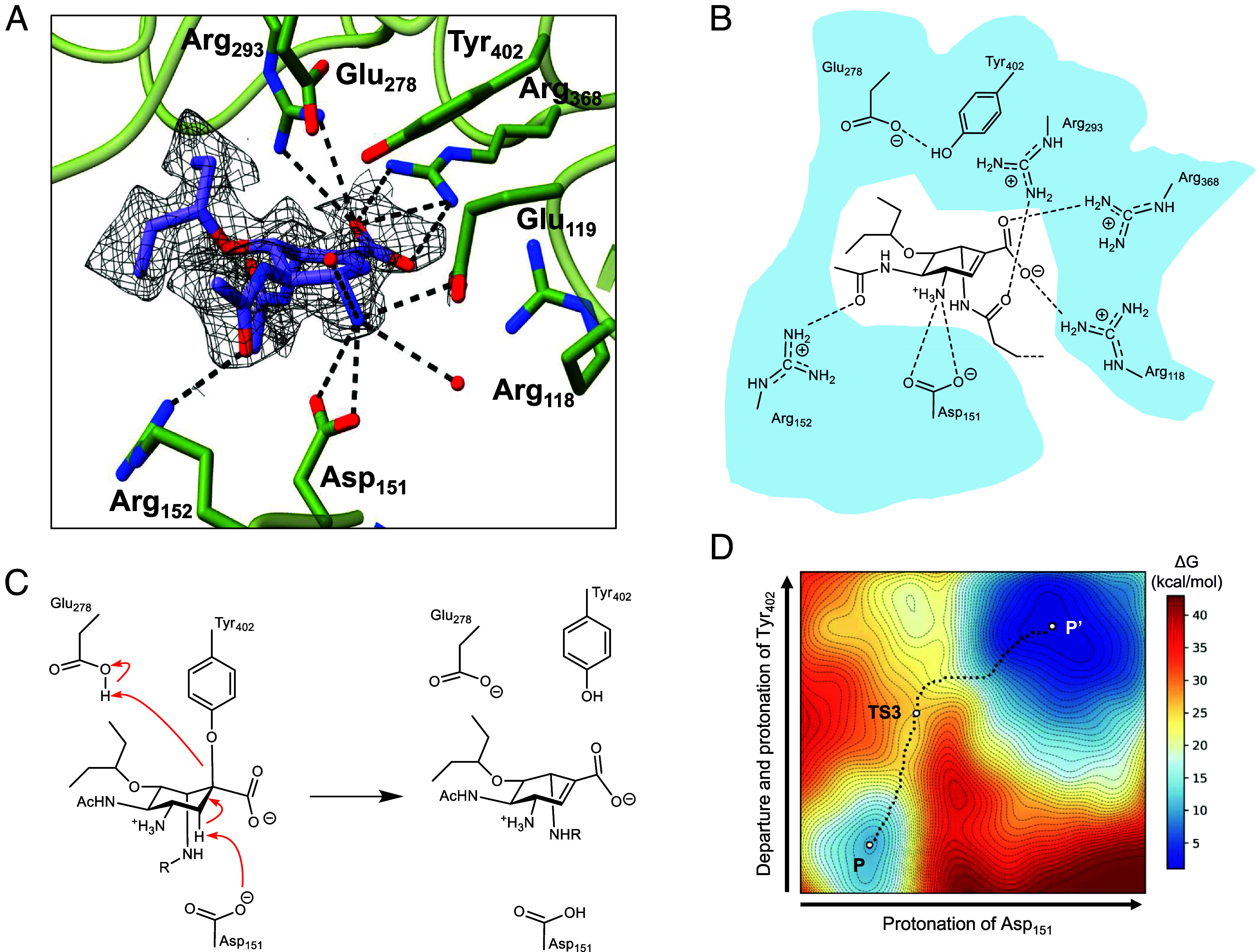
Structural analysis of Oseltamivir aziridine binding and inhibition. (*A*) CryoEM structure of 8 bound in the N1 active site, shown at a density threshold of 0.029, and determined to 2.0 Å. (*B*) Interactions of the Oseltamivir aziridine-derived ligand with N1 catalytic residues. (*C*) Elimination reaction of Oseltamivir aziridine 8 with influenza neuraminidase catalytic nucleophile (Y402) and general acid/base (D151). (*D*) Free energy surface of the main states observed in the elimination reaction.

We used cryoelectron microscopy (cryoEM) to visualize how the aziridine analogues bind in the neuraminidase active site. Our goal was to test whether the binding pose matches our design and to look for evidence of covalent capture. For this, we took advantage of previous work in which a viral N1 had been engineered to aid structure determination by cryoEM (27). We adapted the published system for use in a baculovirus/*Trichoplusia ni* expression system. Data were collected for compounds **8**, **9,** and **13** with the engineered N1 and data collected initially on a 200 kV Glacios electron microscope “in house,” and, subsequently, on a 300 kV Titan Krios electron microscope (Fig. 4*A* and *SI Appendix*, Table S3 and Fig. S7) with resolutions to 2.0, 2.3, and 2.0 Å respectively (28–30).

As expected, N1 assembles into a homotetramer with C4 symmetry; and each monomer adopts a six-bladed beta-propeller fold stabilized by disulfide bonds (27–32). All ligands **8**, **9,** and **13** bound in the active center sialic acid binding pocket, Fig. 4*B*, the −1 subsite [nomenclature according to (33)] of the enzyme including the classical coordination of the carboxylate with Arg368 and with the nucleophile, Tyr402, and the proposed acid/base Asp151, in the correct location for catalysis. Structural observation dovetails with both classical MD and QM/MM modeling in showing the consistent location of the acyl aziridines and the catalytic apparatus.

What was immediately apparent is that none of the three compounds is observed as the covalent adduct expected from the diverse solution experiments and simulations. Instead, the maps show a noncovalent species, with a nucleophile to “C2” distance of 3Å. The 300 kV analyses suggest elimination of the covalent adduct to form an unsaturated compound. This is a well-known feature of neuraminidases both with their natural substrate (to form their own inhibitor DANA [2,3-dehydro-2-deoxy-*N*-acetylneuraminic acid (34)] and a common feature of several X-ray analyses. Indeed, the previous trapped 3-fluoro adducts were also observed as majority elimination products despite being 100% covalent in solution (2). We suspect that electron beam damage in cryoEM enhances the elimination reaction rate in the same way as X-ray exposure.

In light of the ubiquity of the elimination, across many systems and substrates, we adapted our QM/MM approach to quantify the reaction and place these observed findings on a sound theoretical footing. As discussed previously, elimination was a side reaction of the original simulations. We therefore modeled the elimination reaction for the adduct of *N*-acyl aziridine **8**. Two additional CVs were defined: one for the abstraction of a hydrogen atom from C3 of ligand **8** by the carboxylate group of Asp151, and another for the departure and subsequent reprotonation of Tyr402 assisted by Glu278. Our calculations indicate that this process can indeed proceed, in this case through a single transition state with a barrier of ∼15 kcal/mol relative to the covalent adduct (Fig. 4 *C* and *D*).

Despite elimination preventing observation of the covalent adduct in cryoEM, the combined biochemical and MS evidence demonstrates covalent capture by the *N*-acyl aziridines alongside strong potency, motivating their application to activity-based NA quantification and live-virus assays.

### Application of Oseltamivir Aziridines to Enzyme Imaging and Antiviral Action.

**Fig. 5.**
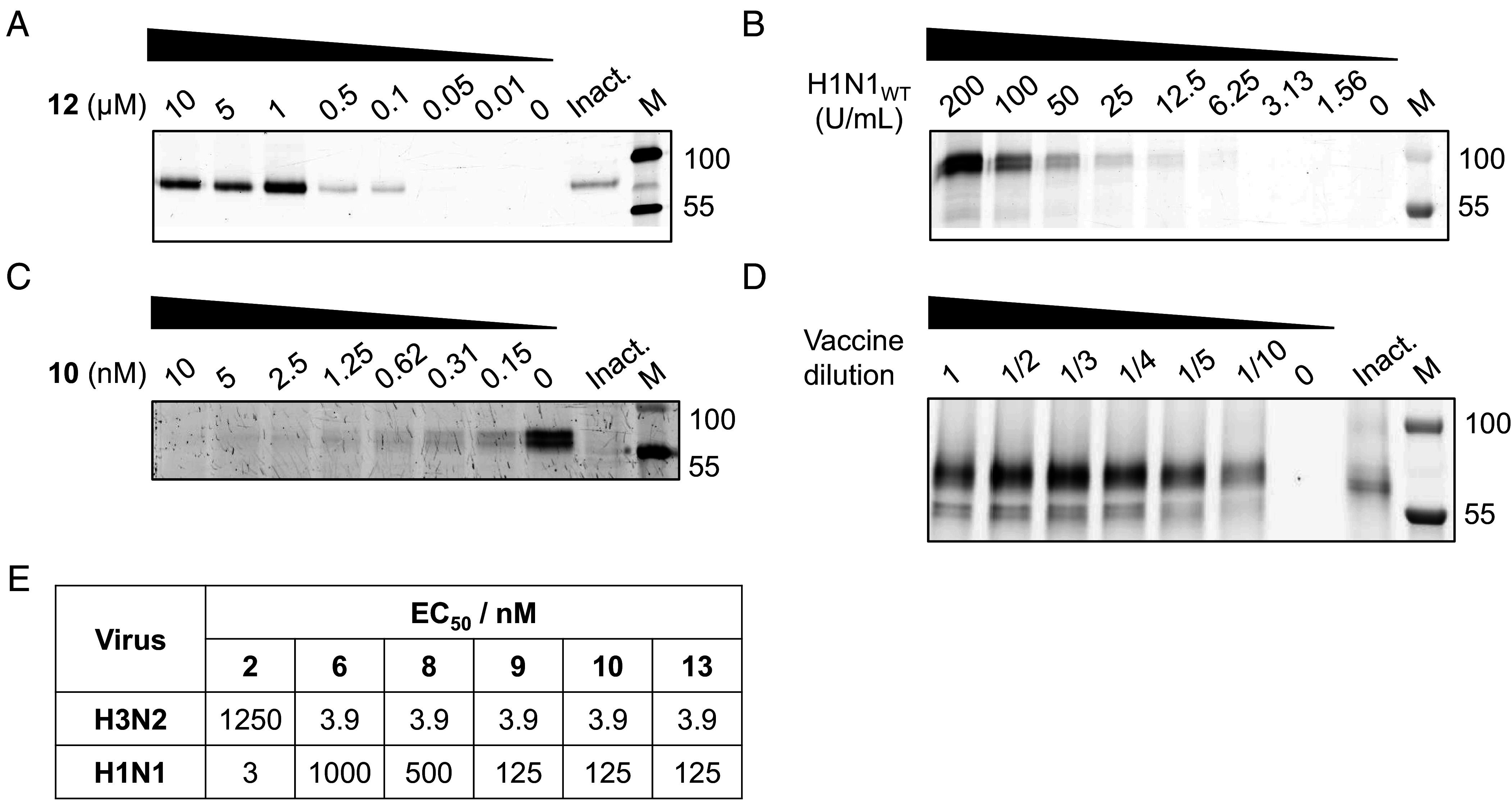
Application of Oseltamivir aziridines to enzyme imaging and antiviral action. (*A*) Labeling of purified N1 using probe **12** at different concentrations. (*B*) Labeling in H1N1 overexpression lysate using probe **12** at different lysate concentrations. (*C*) Competition of labeling by **10**. (*D*) Labeling in a commercial quadrivalent influenza vaccine preparation using probe **12** (1 µM). (*E*) EC_50_ values for microneutralization assays in MDCK-SIAT1 or MDCK cells following a serial dilution of 100 to 0.8 nM for compounds **6**, **8** to **10**, and **13**. Viral plaques were assessed after 22 h by immune-staining and approximate EC_50_s determined by the single inhibitor concentration closest to a 50% reduction in viral plaques. Full fluorescence and Coomassie stained total protein gels are shown in *SI Appendix*, Fig. S9.

The covalent and irreversible nature of the *N*-acyl aziridines enables activity-based protein profiling (ABPP) to report active neuraminidase in biological samples. Using ABP **12**, we label recombinant H1N1 and H5N1 neuraminidases, including their H275Y mutants (Fig. 5 *A* and *B* and *SI Appendix*, Fig. S9 *A*–*C*). Labeling is competed by Zanamivir (*SI Appendix*, Fig. S9*E*) and by Oseltamivir carboxylate (*SI Appendix*, Fig. S9*D*), demonstrating active-site specificity. As expected, labeling is also competed in a concentration-dependent manner by the aziridines themselves (**8, 9, 10, 13**; Fig. 5*C* and *SI Appendix*, Fig. S9 *F*–*I*), consistent with the probes’ mode of action.

We next sought to determine ABP **12** can detect viral N1 within complex mixtures such as seasonal influenza vaccines. Although it is counterintuitive that enzyme activity is used to assess vaccine quality given that the protein may be treated with formaldehyde, global NA level determination is currently used and indeed the NAction! Group concluded that “*NA activity is representative of the native structure and is an excellent measure of the ability of NA to induce NI antibodies …”* (14). Indeed, elegant ultrasensitive enzymatic assays have been proposed for this purpose (35, 36). The drawback with activity measurements is that they are global, reflective of the combined NA pool and not individual enzymes. We hypothesized that ABP methods would overcome this drawback by allowing individual enzyme identification and quantification and in that light, we analyzed a tetravalent vaccine preparation using ABPs. The inactivated tetravalent vaccine Fluarix® Tetra (season 2024–2025) shows multiple labeled bands after incubation with ABP **12** (Fig. 5*D*). Pre-incubation with **9** abolishes most of these signals (*SI Appendix*, Fig. S9*K*), confirming on-target engagement of active neuraminidases in the preparation. For quantification, we labeled vaccine alongside a dilution series of recombinant N1 to generate a standard curve of normalized fluorescence (three repeats; *SI Appendix*, Fig. S9 *L* and *M*). The vaccine bands at ~75 kDa correspond to 0.62 μM ± 0.08, and those at ~60 kDa to 0.11 μM ± 0.01, consistent with multiple NA species in the tetravalent formulation. This labeling can be completely abolished by pre-inactivating the sample (heating the vaccine to 95 °C for 5 min), prior to incubation with **12**.

Ultimately, given the specificity, covalency, and tight binding observed in vitro, arising from our dual transition-state mimicry and covalent inhibition strategy, we next assessed whether these compounds prevent viral replication in live virus assays. We therefore evaluated oseltamivir (2), oseltamivir aziridine (6), and the *N*-acyl aziridines (8–10, 13) in a WHO-compliant viral microneutralization assay. For H1N1 influenza A, MDCK cells were infected and then incubated with compounds to inhibit neuraminidase-dependent release at the cell surface. As shown in Fig. 5*E*, *N*-acyl aziridines **9**, **10**, and **13** were the most active in this format, giving EC_50_ values of 125 nM, albeit higher EC_50_s than control oseltamivir **2** in this assay. We extended these studies to H3N2 using MDCK-SIAT1 cells, where aziridine **6** and the *N*-acyl aziridines (8–10, 13) achieved extremely potent antiviral activity (EC_50_ approximately 3.9 nM under our assay conditions, Fig. 5*E*). Importantly, in this microneutralization format the aziridines show markedly greater potency than oseltamivir against H3N2 (Fig. 5*E*); reported oseltamivir EC_50_ values in similar assays are in the submicromolar range [for example, ~420 nM against H3N2 (37)]. Notably, the relative strain ranking in the cellular assay differs from that in the purified-enzyme kinetic measurements: while the compounds inhibit recombinant H1N1 neuraminidase very tightly (reflected in IC_50_) in vitro, they show higher EC_50_ values against H1N1 than H3N2 in the microneutralization format whereas oseltamivir displays the opposite trend (Fig. 5*E*). Such differences between enzyme IC50 and cellular EC50 might reflect strain- and host-cell–dependent determinants of viral spread (e.g., HA–NA balance and receptor context), beyond inhibition of isolated NA in vitro. These findings align with the design logic: tight binding from transition-state mimicry and durable inactivation from tyrosine-directed covalency (*N*-acyl aziridines, exemplified by **9**), supporting both activity-based quantification and antiviral efficacy.

## Discussion

The development of cyclophellitol-based covalent inhibitors represents a paradigm shift in influenza antiviral therapy, addressing two critical challenges: the need for potent, broad-spectrum inhibitors and tools for precise diagnostic applications. Unlike current commercialized therapies, which rely on reversible, noncovalent inhibition, our compounds achieve irreversible enzyme inactivation by targeting the catalytic tyrosine nucleophile. The compounds provide strong inhibition and antiviral action against all flu strains tested, including the H5N1 of current interest as well as enabling imaging and vaccine assessment.

Although influenza vaccine potency is conventionally standardized by HA content, NA can also be immunogenic and may contribute substantially to protective immunity (38, 39). Indeed, NA determination in vaccines is mandated in several jurisdictions. By establishing a method to quantify individual active NA within vaccines, our approach moves closer to resolving how NA content shapes the overall immune response. Previous methods proposed rely on burst-phase kinetics with fluorogenic 3-fluorosialic acids (36), or determination of free galactose from disaccharide substrates, provided total activity measurements, but our aziridine-based probes go further, simultaneously resolving and quantifying distinct NA isoforms within complex vaccine mixtures. This dual functional and molecular resolution expands opportunities for vaccine standardization and mechanistic understanding of immune correlates.

The broader emergence of covalent inhibitors in drug discovery highlights the therapeutic promise of mechanism-based enzyme inactivation (40). Such irreversible engagement confers pharmacokinetic advantages, particularly for viral enzymes. Beyond influenza, the sialic-acid-based cyclophellitol scaffold is adaptable for viral or bacterial pathogens that exploit similar enzymatic mechanisms. This versatility, coupled with the potential of structural modification at the aziridine moiety, paves the way for a new class of covalent probes that can be tailored to diverse therapeutic and diagnostic needs.

Our first-in-class aziridines already match or exceed the potency of approved neuraminidase inhibitors while offering clear potential for further optimization. The aziridine nitrogen provides a chemically accessible handle for derivatization at a position unavailable in Oseltamivir, Zanamivir, or the fluoroneuraminic acids. Indeed, Oseltamivir-derived aziridines retain an α-carboxylate group that modulates the p*K*a of the free aziridine, influencing its electrophilicity and susceptibility to nucleophilic attack. It is to accommodate these reactivity differences that we prepared a suite of aziridines spanning free and *N*-acyl variants, the latter being intrinsically more electrophilic. This chemical flexibility underpins both mechanistic insight and avenues for structure-guided design. Although our aziridines are highly potent and tightly binding, their relatively slow covalent reactivity leaves scope—especially when informed by QM/MM simulations—for tuning reaction kinetics to optimize pharmacodynamics and minimize off-target effects.

In contrast to previously reported cyclophellitol and cyclophellitol-aziridine systems (21) the present inhibitors operate in a distinct electronic environment: the aziridine must engage a phenolic tyrosine nucleophile adjacent to an electron-withdrawing carboxylate. This difference, which may attenuate aziridine basicity and protonation propensity, endows an additional layer of chemical control that could be harnessed for selectivity. Further work is underway to extend potency toward inhibitor-resistant influenza strains, while retroengineering of the Oseltamivir scaffold toward closer neuraminic acid analogues may yield potent ABPs for human neuraminidases and other pathogen sialidases. Together, these first-in-class inhibitors establish a foundation for next-generation anti-pathogen agents and molecular diagnostics, defining a new chemical and mechanistic space for targeting sialidase enzymes across biology.

## Materials and Methods

Detailed methods for organic syntheses, computational studies, and biochemical experiments, including gene expression and protein purification, structure determination, kinetic assays, in-gel ABPP and microneutralization assays, are given in the *SI Appendix*.

### Computational Methods.

Initial coordinates were taken from PDB ID 3CL2, with the N294S mutation reversed. Protonation states were assigned using the H + + webserver. The system was prepared for classical MD simulations using AmberTools, placing the protein–inhibitor complex in a water box. FF14SB, TIP3P, and GAFF force fields were applied, and RESP charges were derived from Gaussian09. Energy minimization and stepwise heating to 300 K were performed, followed by equilibration in NVT and NPT ensembles. A 100 ns production run was conducted with unrestrained dynamics, using AMBER20 for simulations and VMD/cpptraj for analysis.

A representative snapshot from MD was used for QM/MM MD simulations with CP2K v9.1. The QM region included the inhibitor and catalytic residues (D151, E278, Y402). DFT-level calculations were performed using the PBE functional and a TZV2P basis set, with the remaining system treated classically. The structure was optimized, followed by a 5 ps QM/MM MD simulation at 300 K.

Ligand conformational dynamics were explored using QM/MM metadynamics with CP2K and Plumed. Three CVs defined puckering motions of the six-membered ring. Gaussian potentials were added iteratively until FEL convergence was achieved, represented in a Mercator plot.

Reaction mechanisms, including covalent adduct formation and elimination, were studied using OPES Explore in CP2K with PLUMED. Collective variables modeled proton transfer and nucleophilic attack. FEL were obtained from STATE files.

### Neuraminidase Expression and Purification.

Recombinant neuraminidase (N1) was expressed in a baculovirus system using a construct optimized for high-yield expression (27). Bacmid transformation in DH10Bac cells was verified via PCR, and recombinant baculovirus was generated in Sf9 cells. High Five cells were infected for protein production, and purification involved His-tag affinity chromatography, size-exclusion chromatography, and ion exchange. Final protein preparations were assessed for purity and concentrated for structural and functional studies.

### CryoEM Structure Determination.

Enzyme–inhibitor complexes were prepared with N1 and inhibitors (compounds **8**, **9**, and **13**) before cryoEM grid preparation. Data were collected using a Titan Krios microscope with a GATAN K3 detector. Image processing was performed in RELION3 (41), and structures were refined with Bayesian polishing, yielding resolutions of 2.0 to 2.3 Å.

### Biochemical Assays.

Neuraminidase activity was assessed using MUNANA-based fluorescence assays to determine apparent IC_50_ values, *K_M_*, and kinetic parameters (*K*_I_ and *k*_inact_). Assays were conducted at 37 °C in MES buffer at pH 6.5. Data were analyzed using nonlinear regression in GraphPad Prism.

Recombinant neuraminidase and influenza vaccines were incubated with ABP **12** to assess enzyme activity and inhibitor binding. Samples were resolved by SDS-PAGE and analyzed via fluorescence imaging. Vaccine labeling intensities were quantified and normalized against standard enzyme concentrations.

MDCK-SIAT1 and MDCK cells were inoculated with Influenza A(H3N2) and A(H1N1) respectively. Following a three-h incubation, the inoculum was removed and inhibitors were added in an overlay and incubated for 19 h, before immunostaining for viral nucleoprotein. EC_50_ values were determined as the inhibitor concentration closest to reducing viral replication by 50%.

For further methodological details, refer to the *SI Appendix*.

## Supplementary Material

Appendix 01 (PDF)

## Data Availability

CryoEM maps have been deposited to the EMDB with accession codes: EMD-52254 (42), EMD-52255 (43) and EMD-52253 (44). Coordinates fit to the maps have been deposited in the PDB with accession codes: 9HLG (28), 9HLH (29) and 9HLI (30).
