# Supplementary material for: Oseltamivir aziridines are potent influenza neuraminidase inhibitors and imaging agents
Source: Proc Natl Acad Sci U S A. 2026 Mar 23;123(13):e2504045123. doi: 10.1073/pnas.2504045123 (PMC13038069; doi:10.1073/pnas.2504045123)
Supplement: Supplementary file 1 — Appendix 01 (PDF) [file pnas.2504045123.sapp.pdf]

Supplementary Information

Oseltamivir aziridines are potent influenza neuraminidase inhibitors and imaging agents.

Merijn B. L. Vriends[a,1], Elisha Moran[b,1], Martín Calvelo[c,1], Thomas Hansen[c], Isabelle B. Pickles[b], Xincheng Xin[a], Marieke Biezeno[a], Zachary W. B. Armstrong[a], Maria J. G. D. L. Ferraz[d], Lei Li[a], Alice Lilley[e], Ruth Harvey[e], Dmitri V. Filippov[a], Qinghua Liao[c], Sybrin P. Schröder[a], Gijsbert A. van der Marel[a], Marta Artola[d], Johannes M. F. G. Aerts[d], James N. Blaza[b], Jeroen D. C. Codée[a], Carme Rovira[c,*], Herman S. Overkleeft[a,*], Gideon J. Davies[b,*]

[a] Department of Bio-organic Synthesis and [d] Department of Medical Biochemistry, Leiden Institute of Chemistry, Leiden University; P.O. Box 9502, 2300 RA Leiden, The Netherlands

[b] Department of Chemistry, University of York; Heslington, York, YO10 5DD, U.K.

[c] Departament de Química Inorgaǹica i Orgaǹica (Secciόde Química Orgaǹica) and Institut de Química Teoȓica I Computacional (IQTCUB), Universitat de Barcelona; Martí i Franqueś 1, 08028 and Fundació Catalana de Recerca i Estudis Avançats (ICREA); Passeig Lluís Companys 23,08010 Barcelona, Spain

[e] Worldwide Influenza Centre, The Francis Crick Institute; 1 Midland Road, NW1 1AT, London UK

[1] M.B.L.V., E.M. and M.C. contributed equally to this paper.

* Correspondence should be addressed to Carme Rovira, Herman S. Overkleeft or Gideon J. Davies

Email: c.rovira@ub.edu, h.s.overkleeft@lic.leidenuniv.nl or gideon.davies@york.ac.uk

Table of Contents

Supplementary Figures 2

Computational Methods 15

Biochemical Methods 17

Synthetic Methods 24

NMR Spectra 33

Supplementary References 62

Supplementary Figures

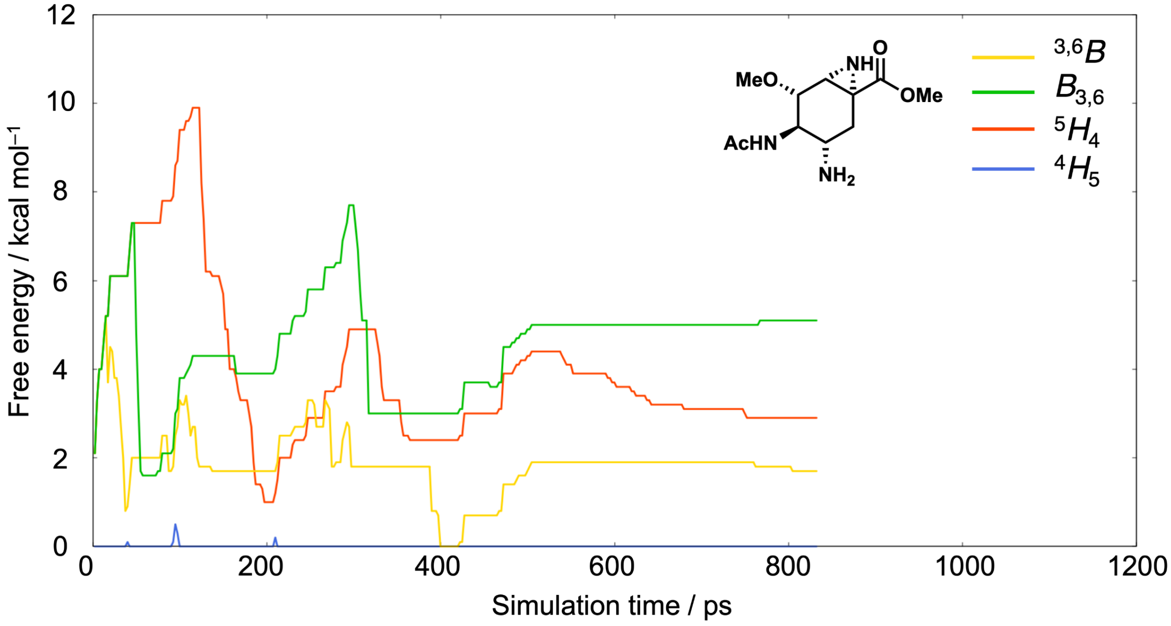

Figure S1. The energy variation of the important conformations of the reconstructed free energy surface along the QM metadynamics simulations of Oseltamivir aziridine 14.

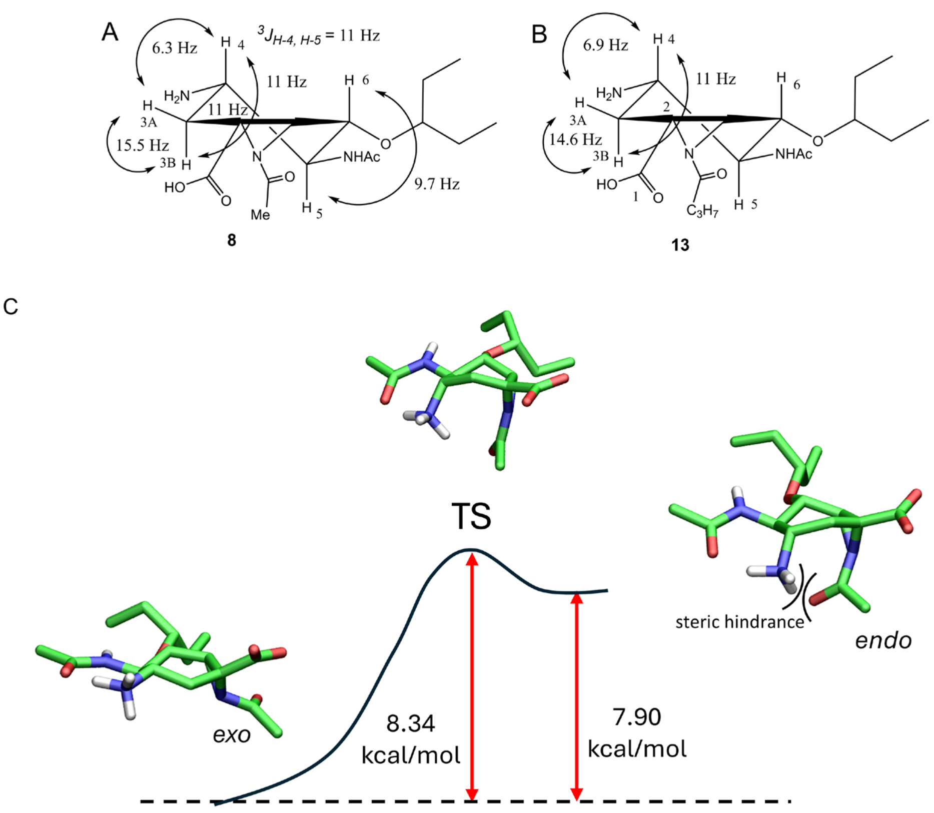

Figure S2. Oseltamivir aziridines adopt a $^4H_5$ 'exo' conformation. (**A**+**B**) The NMR-derived $^4H_5$ conformer of compounds **8** (A) and **13** (B) with the relevant $^3J_{HH}$-couplings indicated. (**C**) Calculated relative energies of exo and endo-aziridines and the associated transition state (TS).

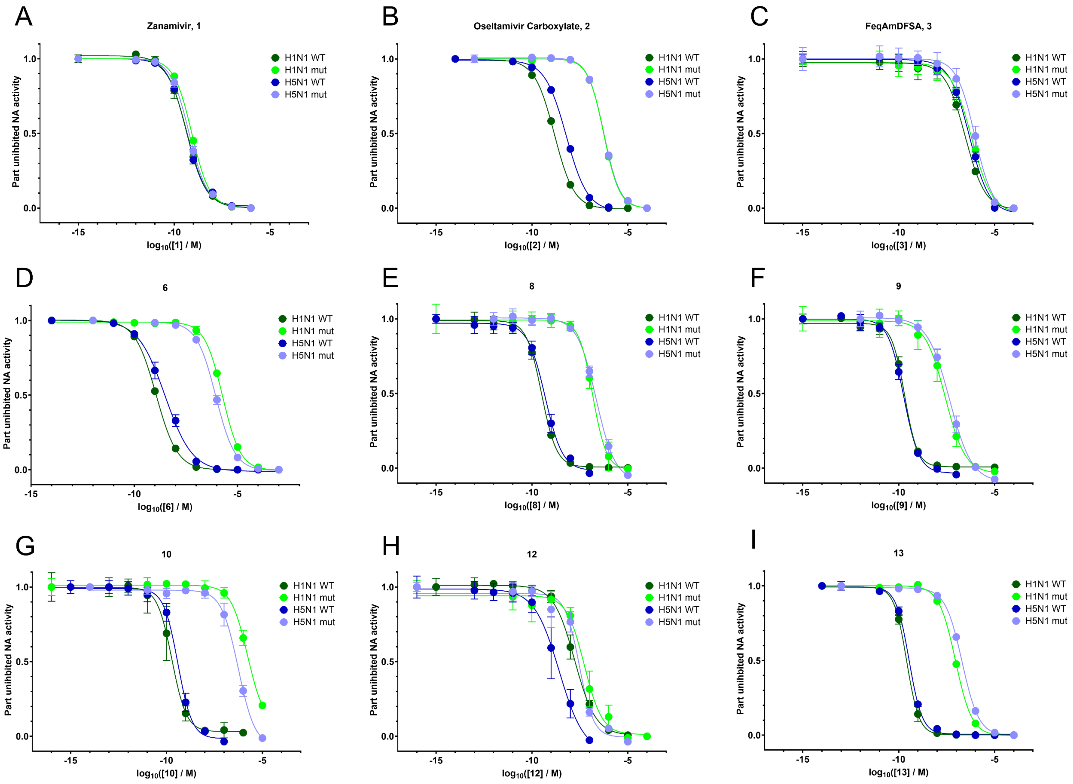

Figure S3. Kinetic studies of neuraminidase inhibitors and probes. (**A**-**I**) IC_{50} curves for inhibition of neuraminidase activity by compounds **1** (**A**), **2** (**B**), **3** (**C**), **6** (**D**), **8** (**E**), **9** (**F**), **10** (**G**), **12** (**H**) and **13** (**I**) in H1N1 wild type (dark green), H1N1 H275Y mutant (lime green), H5N1 wild type (dark blue) or H5N1 H274Y mutant (light blue) overexpression lysates, determined using the fluorogenic substrate MUNANA.

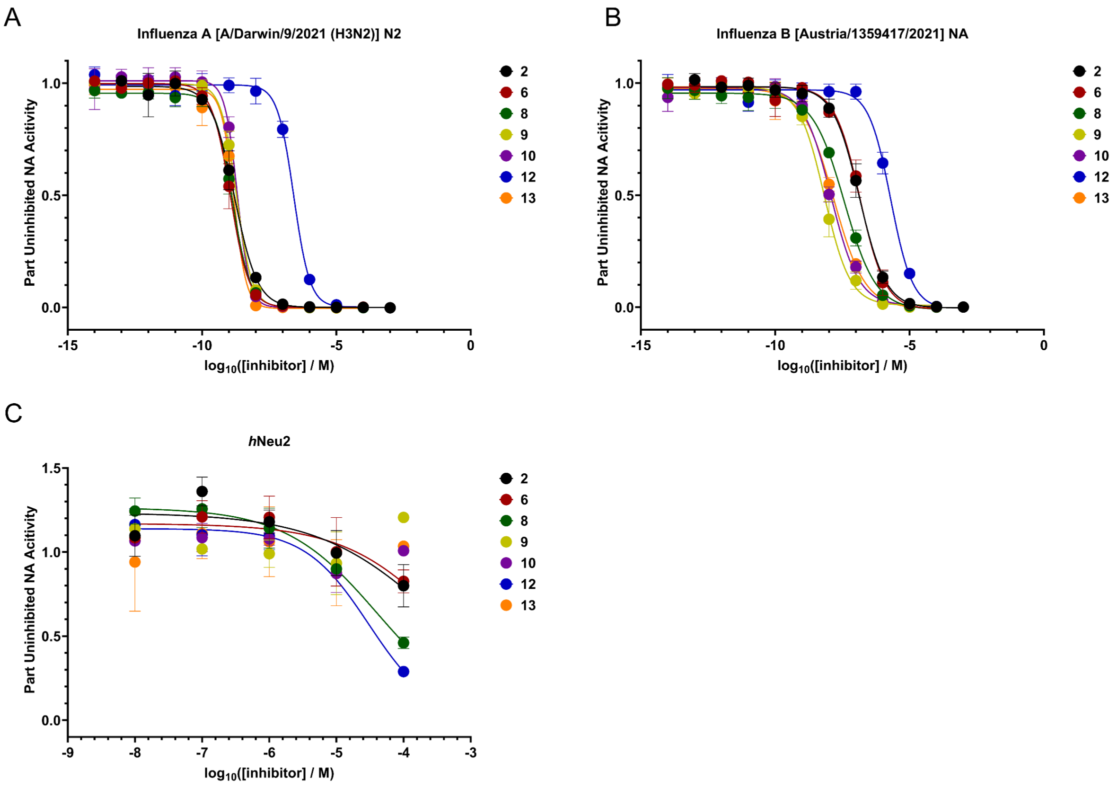

Figure S4. Kinetic studies of neuraminidase inhibitors and probes. IC_{50} curves for inhibition by compounds **2**, **6**, **8**, **9**, **10**, **12** and **13** of influenza A (H3N2) neuraminidase (**A**), influenza B neuraminidase (**B**) and human Neu2 (**C**), determined using the fluorogenic substrate MUNANA.

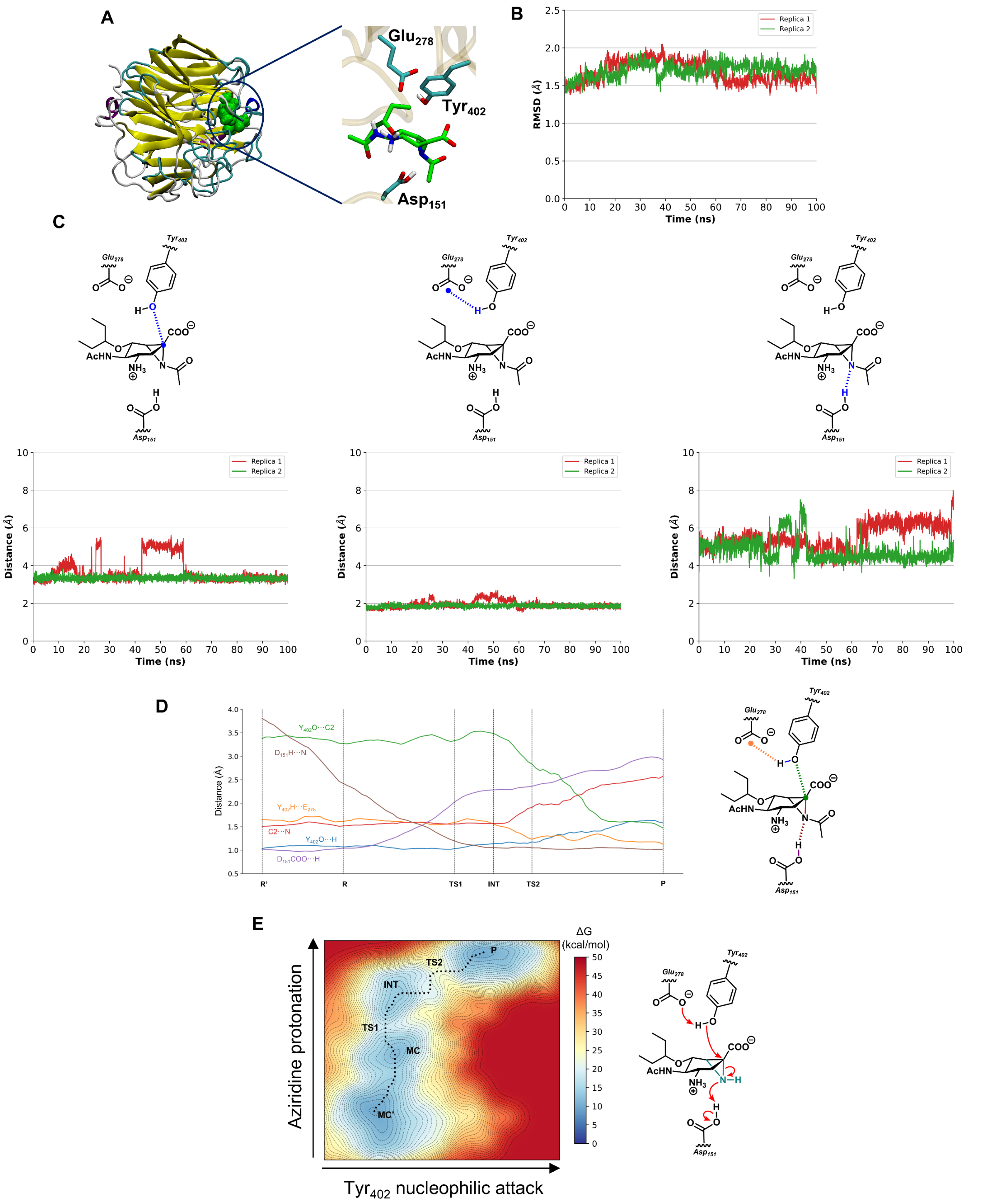

Figure S5. Computational studies on the covalency of the acyl aziridines and free aziridine 6. (**A**) Representation of the initial structure employed for the classical MD simulation. (**B**) Evolution of RMSD over the simulation time. (**C**) Evolution of the three catalytic distances over classical MD simulation time. (**D**) Evolution of the catalytic distances along the minimum path energy for the formation of the covalent adduct. (**E**) Free energy surface of the main states observed in the covalent reaction of free aziridine **6**.

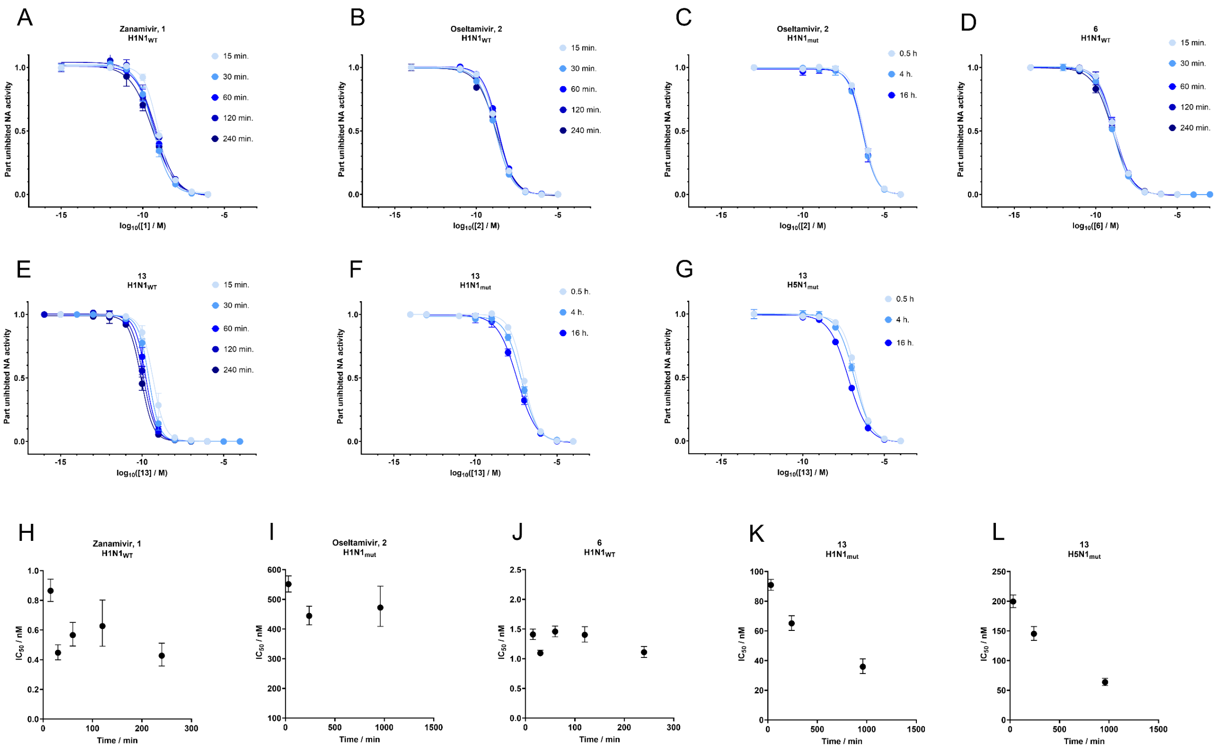

Figure S6. Time-dependency of inhibition. (**A-G**) Time dependent IC_{50} curves for inhibition of neuraminidase activity by compound **1** in H1N1 wild type overexpression lysate (**A**), **2** in H1N1 wild type (**B**) and H275Y mutant (**C**) lysate, **6** in H1N1 wild type lysate (**D**), and **13** in H1N1 wild type (**E**) and H275Y mutant (**F**) and H5N1 H274Y (**G**) mutant lysates. (**H-L**) Time dependent IC_{50} values (nM) for inhibition of neuraminidase activity by compound **1** in H1N1 wild type overexpression lysate (**H**), **2** in H1N1 H275Y mutant (**I**) lysate, **6** in H1N1 wild type lysate (**J**), and **13** in H1N1 H275Y mutant (**K**) and H5N1 H274Y (**L**) mutant lysates. IC_{50} curves are plotted as the average of 2 biological repeats (each the average of 3 technical replicates) ± SD. Calculated IC_{50} values were plotted as the mean of 2 biological repeats ± SD.

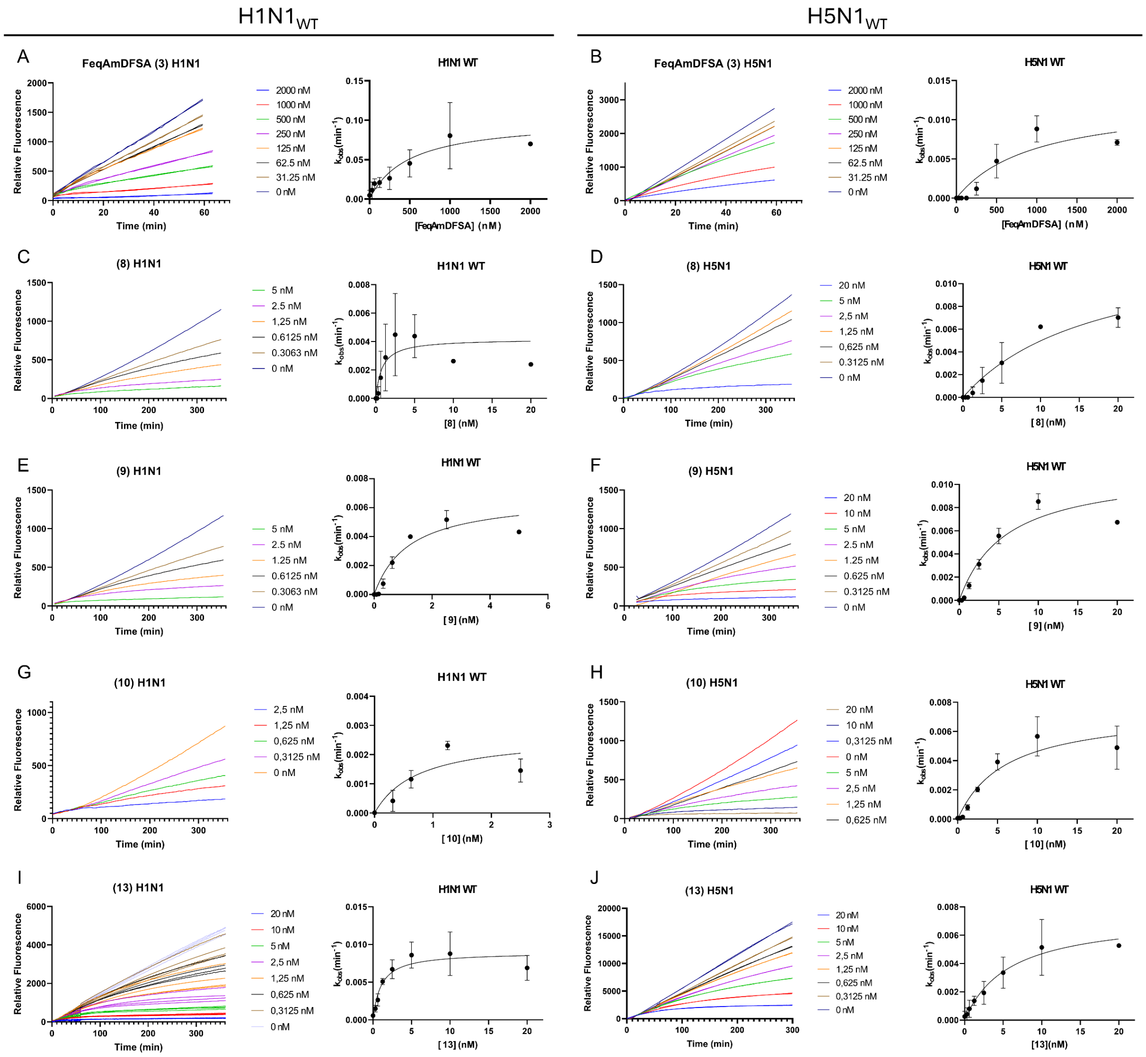

Figure S7. Inactivation kinetics of neuraminidase inhibitors. Determination of K_{obs} (left), K_I and k_{inact} (right) for **3** (**A+B**), **8** (**C+D**), **9** (**E+F**), **10** (**G+H**) and **13** (**I+J**) against H1N1 and H5N1 wild type overexpression lysate, determined using the fluorogenic substrate MUNANA. Relative fluorescence was plotted versus time (representative example) to calculate K_{obs}. K_{obs} was plotted as the average of 2 biological repeats ± SD.

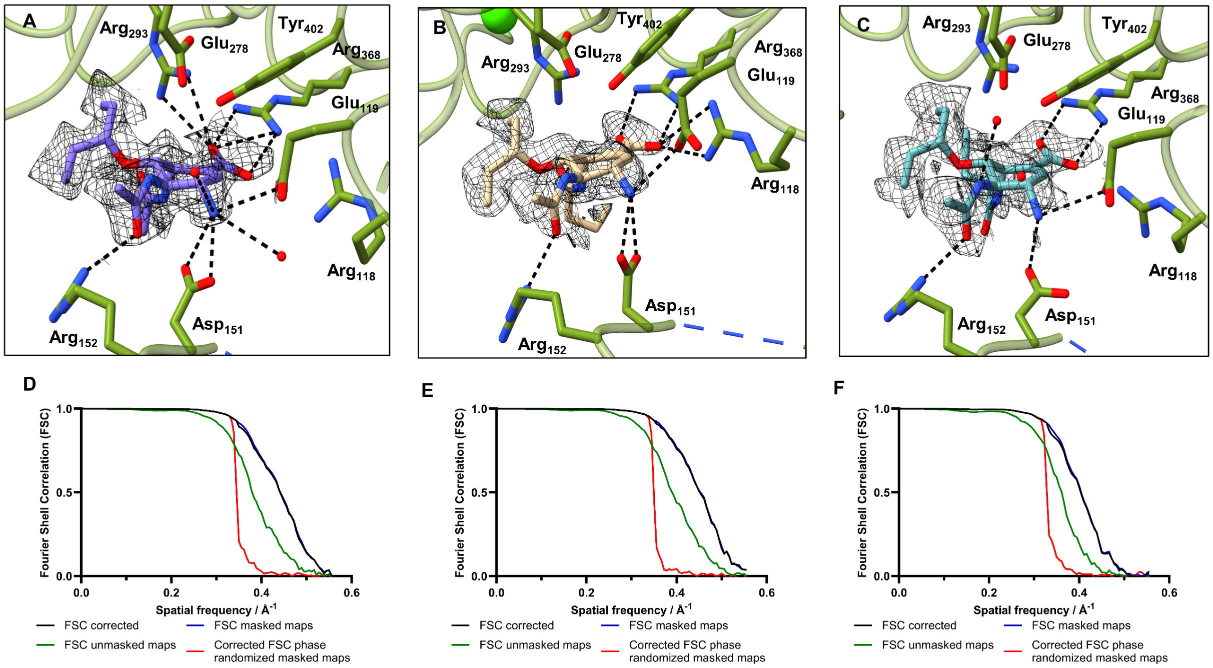

Figure S8. Structural studies of the covalent reactivity of Oseltamivir acyl-aziridines using cryo-EM and mass spectrometry. Cryo-EM reconstructions of neuraminidase with compounds **8** (**A**), **13** (**B**) and **9** (**C**) solved to 2.0, 2.0 and 2.3 Å respectively. Compounds **8**, **13**, and **9** are shown with density at a threshold of 0.029, 0.0358, and 0.0278, respectively. (**D**-**F**) Fourier shell correlation plot: Corrected FSC (Black), unmasked FSC (green), Masked FSC (Blue), and phase-randomised mask FSC (Red) for the cryo-EM reconstruction of neuraminidase with compounds **8** (**D**), **13** (**E**) and **9** (**F**).

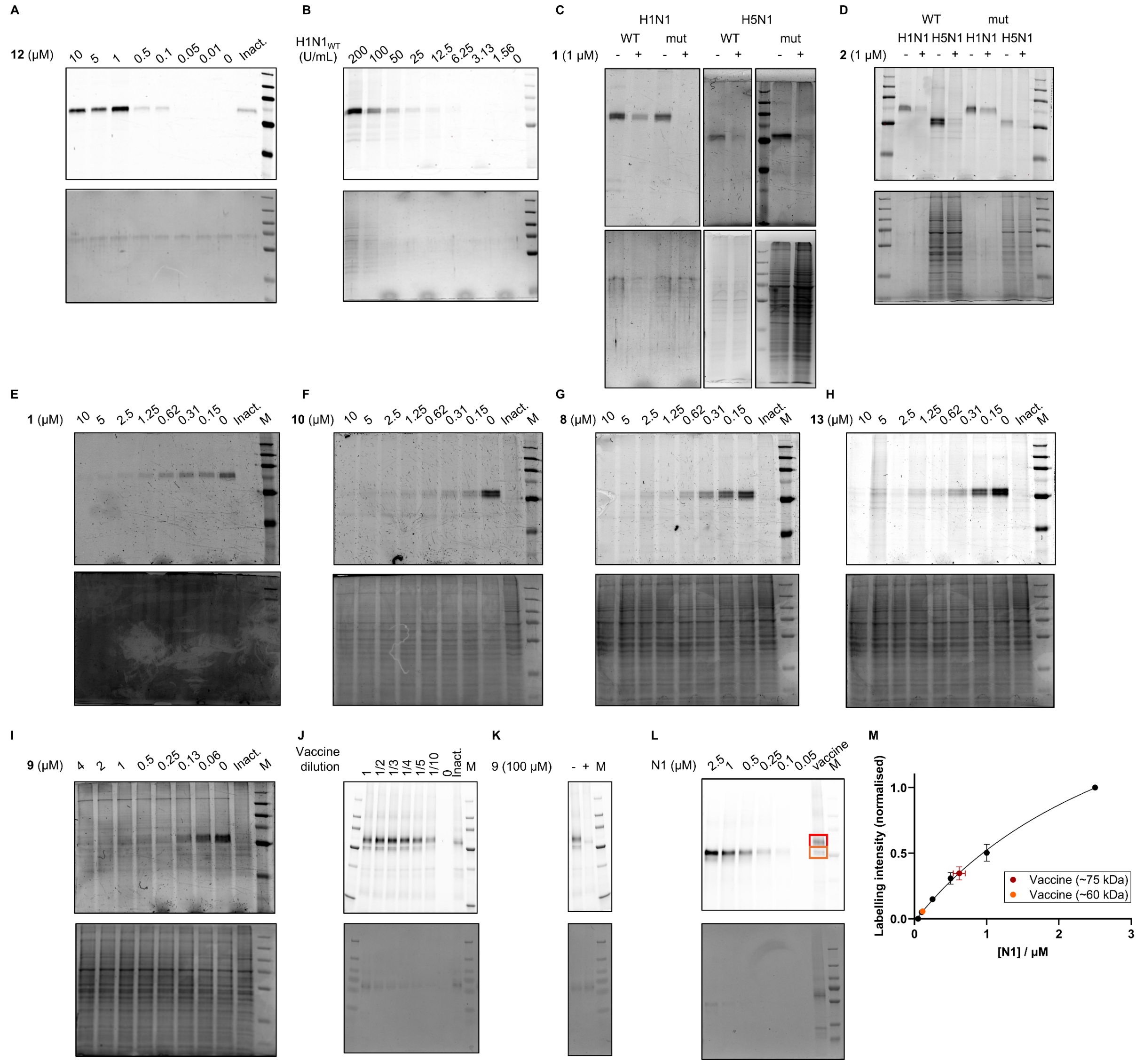

Figure S9. Full fluorescence (top) and Coomassie stained (bottom) ABP-labeling SDS-PAGE gels. (**A**) Concentration dependent labeling of neuraminidase in H1N1 wild type pure protein by ABP **12**. (**B**) Labeling of decreasing amounts of neuraminidase in H1N1 overexpression lysates by ABP **12**. (**C**+**D**) Inhibition by Zanamivir **1** (**C**) or Oseltamivir **2** (**D**) of labeling of neuraminidase by ABP **12** in H1N1 and H5N1 wild type and mutant overexpression lysates. (**E**-**I**) Inhibition of labeling by **12** with different concentrations of **1** (**E**), **10** (**F**), **8** (**G**), **13** (**H**) or **9** (**I**). (**J**) Labeling of neuraminidases in diluted samples of influenza vaccine preparation by **12**. (**K**) Inhibition of labeling by **12** in vaccine preparation after pre-incubation with a 10X excess of compound **9** or water. (**L**-**M**) Quantification of neuraminidase content in an influenza vaccine preparation as compared to labeling of purified neuraminidase by ABP **12** (representative example). Competition of labeling of vaccine neuraminidases by **9**. (M) Average fluorescence intensity of bands in (L) plotted versus neuraminidase concentration after three repeats ± SD. A standard curve was constructed using these points. Intensity of the bands at ~75 kDa (red) and ~60 kDa (orange) labeled in the

vaccine preparation in (K) were plotted ± SEM versus the neuraminidase concentration calculated from the standard curve ± SEM.

Table S1. Apparent IC_{50} values (nM) for *in vitro* inhibition of H3N2 influenza A (A/Darwin/9/2021) and influenza B (B/Austria/1359417/2021) neuraminidases. Reported values are mean ± standard deviation (SD) from 2 technical triplicates. IC_{50} values were determined using MUNANA as substrate following a 30-minute preincubation of enzyme (1 nM) with varying concentrations of inhibitor. IC_{50} graphs are shown in **Figure S3**.

	Inhibitor						
Virus / Strain	**2**<br>oseltamivir carboxylate	**6**	**8**	**9**	**10**	**12**	**13**
A/H3N2	1.62 ± 0.23	1.15 ± 0.48	1.36 ± 0.25	1.92 ±0.32	2.12 ± 0.58	262 ± 46	1.54 ± 0.13
B NA	140 ± 49	150 ± 56	31.5 ± 7.6	7.24 ± 3.17	12.1 ± 2.0	2992 ± 375	14.8 ± 0.4

Table S2. Apparent second order inactivation kinetics (K_I (nM), k_{inact} (min^{-1}) and k_{inact}/K_I (min^{-1} mM^{-1})) for *in vitro* inhibition of H1N1 and H5N1 influenza neuraminidases, as well as oseltamivir resistant mutants (H1N1-H275Y and H5N1-H274Y). Values were determined using MUNANA as substrate and 1 U/mL (H1N1 wildtype), 0.6 U/mL (H5N1 wildtype) or 2 U/mL (mutants) of enzyme with varying concentrations of inhibitor at 37 °C. Measurements were taken every 5 minutes for the acylaziridines and every 30 minutes for FeqAmDFSA **3**.

**Virus / Strain**	**Inhibitor**							
	**1**<br>Zanamivir	**2**<br>Oseltamivir carboxylate	**3**<br>Withers' FeqAm DFSA	**6**	**8**	**9**	**10**	**13**
H1N1 wildtype	K_I = 0.0761	K_I = 0. 217	K_I = 79.7<br>k_{inact} = 0.101<br>k_{inact}/K_I = 1268	K_I = 0.164	K_I = 0.239<br>k_{inact} = 0.011<br>k_{inact}/K_I = 29890	K_I = 0.379<br>k_{inact} = 0.011<br>k_{inact}/K_I = 29896	K_I = 0.113<br>k_{inact} = 0.002<br>k_{inact}/K_I = 23657	K_I = 0.229<br>k_{inact} = 0.011<br>k_{inact}/K_I = 46466
H5N1 wildtype	K_I = 0.0369	K_I = 0.462	K_I = 80.9<br>k_{inact} = 0.013<br>k_{inact}/K_I = 155	K_I = 0.257	K_I = 0.855<br>k_{inact} = 0.012<br>k_{inact}/K_I = 21731	K_I = 0.503<br>k_{inact} = 0.011<br>k_{inact}/K_I = 21731	K_I = 0.051<br>k_{inact} = 0.007<br>k_{inact}/K_I = 14733	K_I = 0.484<br>k_{inact} = 0.0074<br>k_{inact}/K_I = 15304
H1N1-H275Y	K_I = 0.0174	K_I = 115		K_I = 398				
H5N1-H274Y	K_I = 0.0730	K_I = 67.7		K_I = 108				

Table S3. Cryo-EM processing and refinement statistics for N1 complexed with 8, 9 and 13.

	N1-13	N1-9	N1-8
**Data collection and processing**			
**Microscope**	Krios 1	Krios 1	Krios 1
**Voltage (kV)**	300	300	300
**Electron fluence (e^-/Å^2)**	44	44	44
**Nominal defocus values (μm)**	-1.4, -1.2, -1.0, -0.8,-0.6	-1.4, -1.2, -1.0, -0.8,-0.6	-1.4, -1.2, -1.0, -0.8,-0.6
**Calibrated pixel size (Å)**	0.635	0.635	0.635
**Symmetry imposed**	C4	C4	C4
**Initial particles**	4763583	4261747	5464419
**Final particle images**	216383	229896	235807
**Map resolution (Å)**	2.0	2.3	2.0
**FSC threshold**	0.143	0.143	0.143
**Refinement**			
**Map sharpening *B* factor (Å^2)**	-50	-62	-52
**Model composition**			
**Non-H atoms**	11870	11864	11845
**Residues**	1476	1476	1476
**Ligands**	12	12	12
**Waters**	382	392	377
**Mean *B* factors (Å^2)**			
**Protein**	12.8	18.3	13.1
**Ligands**	26.1	36.5	26.3
**Water**	16.7	20.0	15.3
**RMS deviations**			
**Bond length (Å)**	0.003	0.004	0.005
**Bond angles (°)**	0.678	0.681	0.786
**Validation**			
**MolProbaility score**	1.23	1.36	1.25
**Clashscore**	2.66	2.31	1.31
**Poor rotamers %**	0.8	1.52	1.36
**Ramachandran plot**			
**Outliers %**	0.28	0.28	0.28
**Allowed %**	2.75	3.03	3.93
**Favoured %**	97.0	96.7	95.8
**PDB code**	9HLI	9HLH	9HLG

Computational Methods

System preparation

The initial coordinates of the protein were taken from the structure of PDB ID 3CL2. This corresponds to an X-Ray structure containing a mutant of the N1 enzyme (N294S) in complex with Oseltamivir(1). After reversing the N294S mutation, the Oseltamivir *N*-acetylaziridine ligand **8** was constructed using the coordinates of Oseltamivir as template. The protonation states of histidine, glutamic and aspartic amino acids were assigned employing the H++ webserver(2) and visual analysis of the environment of each residue, considering the optimal pH for the enzymatic activity (≈ 6.5). The catalytic base (E278) and catalytic acid (D151) were considered as deprotonated and protonated, respectively, in homology with the protonation states of the classical reaction mechanism with the natural substrate.

Classical MD simulations

After constructing the initial model, the system was prepared for a classical molecular dynamics (MD) simulation using the LeaP code, available in the AmberTools code(3). The enzyme with the inhibitor at the active centre was placed in a cubic box containing 20612 molecules of water. No ions were needed to neutralize system. The force fields FF14SB(4) and TIP3P(5) were used for the protein and water solvent molecules, respectively, whereas the GAFF(6) force-field was employed for the ligand molecule, using the Antechamber tool(7) and RESP charges calculated with Gaussian09(8) at HF/6-31G* level for its parametrization.

The MD simulation was carried out in several stages. First, an energy minimization using the steepest descent and conjugate gradients methods was performed in five steps: 1) relaxation of the solvent molecules holding the protein and ligand fixed; 2) relaxation of the protein-ligand complex, holding the solvent molecules; 3) relaxation of the ligand, holding the protein and solvent molecules; 4) relaxation of the whole system holding the ligand and 5) relaxation of the whole system without any restraints. After the minimization, the system was heated to 300 K by increasing the temperature in intervals of 50K over 50 ps runs. In the first run, the protein and the ligand were fixed, whereas only the backbone atoms were kept fixes in the subsequent heating runs. Subsequently, a simulation in the NPT ensemble for 500 ps was carried out for converging the water density at 300 K. In this step, the restrains in the protein backbone were kept and the interaction between the inhibitor and the catalytic residues was ensured by restricting the following pair distances to values shorter than 3.5 Å: 1) the distance between the proton of the carboxylic group of D151 and the N of the aziridine group; 2) between the carboxylate group of E278 and the hydrogen of the hydroxyl group of Y402 and 3) between the O of the hydroxyl group of Y402 and the C2 of the inhibitor. Afterwards, the system was equilibrated in the NVT ensemble during 50 ns, with distance restraints between the catalytic residues and the ligand only. The simulation was continued with no restraints for 100 ns in the NVT ensemble. Two replicas assigning different initial velocities were performed. AMBER20 software was employed for all simulations,(9) and analyses were carried out using VMD(10) and cpptraj(11).

QM/MM MD simulations

A representative snapshot of the previous MD simulation was selected as initial point for a QM/MM MD simulation, combining DFT-based Born-Oppenheimer MD with force-field MD, using the software CP2K v9.1.(12) The selected QM region includes the inhibitor and the side chains of the three catalytic amino acids (D151, E278 and Y402), resulting in a total of 82 atoms enclosed in a 16.4 x 19.0 x 18.0 Å3 cell. This QM region was treated at DFT level using the PBE functional,(13) using the dual basis set of Gaussians and plane-waves (GPW) formalism. The Gaussian triple-ζ valence polarized (TZV2P) basis set was used to expand the wave function, employing an auxiliary plane-wave basis set with a density cut-off of 300 Ry,

together with GTH pseudopotentials,(14) in order to converge the electron density. The remaining atoms of the system were treated at the molecular mechanics (MM) level, whereas the dangling bonds between the QM and the MM region were capped with hydrogen atoms. The structure was optimized by a simulated annealing, followed by an unbiased QM/MM MD simulation at 300 K in the NVT ensemble for 5 ps, using a time step of 0.5 fs.

QM/MM OPES Explore simulation of the formation of the covalent adduct

The last frame of the previous unbiased QM/MM MD simulation was chosen as starting point for the study of the reaction mechanism (formation of the covalent adduct with Y406) using the CP2K v9.1 software, coupled to PLUMED v2.8.(15) The OPES Explore(16) method was used to enhance the sampling of the reaction, selecting an initial barrier a 20 kcal/mol, a pace of 200 MD steps, an adaptive sigma of 1000 MD steps, and a temperature of 300K. Two collective variables (CVs) were employed for modelling the reaction. The first CV (CV1) accounts for the deprotonation of Y402 by E278 and the nucleophilic attack of Y402 on the C2 of the ligand ($d_{O \cdots H\text{-}Tyr402} - d_{H\text{-}Tyr402 \cdots COO\text{-}Glu278} - d_{O\text{-}Tyr402 \cdots C2}$). The second CV (CV2) describes the protonation and ring opening of the aziridine group ($d_{N \cdots C2} + d_{COO \cdots H\text{-}Asp151} - d_{H\text{-}Asp151 \cdots N}$). Wall restraints were used in CV1 and CV2 in the region of the products state to ensure convergence of the simulation. Because of this, the energy of P is likely to be overestimated. The simulation was stopped after two crossings over the TS. The corresponding Free Energy Landscape (FEL) was obtained from the STATE file.

QM/MM OPES Explore simulation of the elimination reaction

A frame of the former QM/MM MD simulation in which the product P was formed was chosen as initial structure for the study of the elimination reaction. First, we carried out an unbiased QM/MM MD simulation of the P state. Afterwards, we modelled the reaction employing the OPES Explore method, using the same parameters previously used for the formation of the covalent adduct. Two collective variables were employed. The first CV describes the abstraction of one of the hydrogens of C3 of ligand 6 by D151 ($d_{C3 \cdots H} - d_{C3\text{-}H \cdots COO\text{-}Asp151}$) whereas the second one accounts for the departure and reprotonation of Y402 assisted by E278 ($d_{O\text{-}Tyr402 \cdots C2} - d_{O \cdots H\text{-}Tyr402}$). The simulation was stopped after two crossings over the TS, obtaining the corresponding FEL from the STATE file. Data of all simulations is provided in the Zenodo repository (https://doi.org/10.5281/zenodo.14226578).

QM metadynamics simulations of the ligand conformational free energy landscape

The conformation FEL of the ligand was conducted by QM/MM metadynamics, employing the PBE functional in combination with the TZV2P basis set. This approach aligns with previous studies, which demonstrate its good performance in describing six-membered conformations. The side chains of the C atoms were omitted to avoid spurious intramolecular interactions that cannot take place in the enzyme. A time step of 0.5 fs was selected for the simulations(17). The ligand was first equilibrated without any constraint for at least 5 ps. Next, the metadynamics algorithm was activated to explore the conformation FEL of the ligand using CP2K align with the Plumed 2.5.4 plugin(15). Three puckering collective variables (CVs) were used, *qx/Q*, *qy/Q*, and *qz/Q*, which define all possible conformations of a 6-membered ring. The width of the Gaussian-shaped potential hills was set at 0.035, 0.030, 0.020 rad for *qx/Q*, *qy/Q*, and *qz/Q*, respectively. The Gaussian height was set to 0.6 kcal mol^{-1}, while the time deposition interval between two consecutive Gaussians was set to 25 fs. For a better convergence and accuracy in a system with multiple minima, the Gaussian height was lowered to 0.1 kcal mol^{-1} upon the complete exploration of the FEL (after approx. 500 ps). The simulations were stopped after having added around 33000 Gaussians (approx. 825 ps). Convergence was established according to the invariance of the energy differences between the principal wells of the reconstructed free energy surface along the simulation (standard deviation < 1 kcal mol^{-1}, considering the last 50 ps of simulation). The collective variables were reweighted to obtain the Cremer-Pople puckering coordinates(18) theta and phi (θ, ϕ) by the use of the Plumed driver 2.7.2. The free energy was represented in a Mercator plot, which is an equidistant cylindrical projection that results in a rectangular map with respect to θ and ϕ. This diagram provides the conformational relationship among

all conformations of a six-membered ring(19, 20). **Figure S1** illustrates the evolution of the free energy over time for the most relevant conformations of the six-membered ring in Oseltamivir aziridine **14**.

DFT calculations on the exo/endo configuration of compound 8

Density functional theory (DFT) (21) calculations were performed to determine the free energy of the conversion between the exo and endo configurations of the Oseltamivir *N*-acetylaziridine ligand **8** using ORCA 6.0.1 (22). Both exo and endo configurations of ligand **8** were optimized using functional PBE0 (23) with basis set def2-TZVP (24) and D3 dispersion correction (25, 26). Frequency calculations were carried out subsequently to determine thermodynamic properties. Afterwards, Nudged Elastic Band with Transition State optimization (NEB-TS) (27, 28) was run using the exo configuration as reactant state and the endo configuration as product state. Ten images were used for the NEB-TS calculation with the functional PBE0 with basis set def2-SVP (24) and D3 dispersion correction. Once the NEB-TS calculation was converged, the corresponding TS configuration was subject to further optimization and frequency calculation at the PBE0/D3/def2-TZVP level of theory. The Gibbs free energies including electronic energy, zero-point energy and entropy contribution were used to compare the properties of each conformation (endo, exo and the TS).

Biochemical Methods

Materials

Purified H1N1 neuraminidase ((A/California/04/2009) Neuraminidase / NA) for IC_{50} determination and *in vitro* labeling was obtained from Thermo Scientific. Overexpression lysates for influenza H1N1 ((A/California/04/2009) neuraminidase / NA, influenza A H1N1 neuraminidase / NA (H275Y) and influenza A H5N1 (A/Anhui/1/2005) neuraminidase / NA) were obtained from Sino Biological. Pure Influenza A/H3N2 (A/Darwin/9/2021) and B (B/Austria/1359417/2021) neuraminidases for IC_{50} determination were obtained from 2BScientific. 4-(Methylumbelliferyl)-*N*-acetylneuraminic acid (MUNANA) and zanamivir were obtained from Carbosynth. Oseltamivir carboxylate was synthesised following the procedure described by Mooney *et al.*(29). Commercial influenza vaccine Fluarix Tetra (GSK), influenza season 2024-2025 was purchased as a commercially available preparation in Spain.

Neuraminidase expression and purification for cryo-EM structure determination

Construct Design of Viral Neuraminidase Constructs

A construct was designed using the sequence of N1 from influenza A/California/04/2009 based on Ellis *et al.*.(30) The N-terminus contained a melittin signal sequence followed by a hexahistidine tag, tetramerisation domain and thrombin cleavage site into a pFastBac™ vector, which was produced, and codon optimised by GenScript.

N1 construct sequence

**MKFLVNVALVFMVVYISYIYADPGHHHHHHSSSDYSDLQRVKQELLEEVKKELQKVKEEIIEAFVQELRKRGGTAEN
LYFQGVKLAGNSSLCPVSGWAPLSKDNSVRIGSKGDVFVIREPFISCSPLECRTFFLTQGALLNDKHSNGTIKDRSP
YRTLMSVPIGSVPSPYNARFESIAWSASACHDGINWLTIGITGPDNGAVAILKYNGIITDTIKSWRNNILRTQESEC
ACVNGSCFTVMTDGPSNGQASYKIFRIEKGKIVKSVEMNAPNYHYEECSCYPDSSEITCVCRDNWHGSNRPWVSFNQ
NLEYQIGYICSGIFGDNPRPNDKTGSCGPVSSNGANGVKGFSFKYGNGVWIGRTKSISSRNGFEMIWDPNGWTGTDN
NFSIKQDIVGINEWSGYSGSFVMHPELTGLDCIVPCFWVELIRGRPKENTIWTSGSSISFCGVNSDTVGWSWPDGAE
LPFTIDK**

Generation of N1 Baculovirus

DH10Bac™ competent cells were used to produce the recombinant bacmid, containing the tetracycline-resistant helper plasmid (bMON7142) and kanamycin-resistant baculovirus shuttle vector (bMON14272). N1 construct was transformed into DH10Bac™ competent cells using electroporation at 1.8 kV briefly before adding SOC medium and incubation of the cells for 4 hours at 37 °C. Blue-white screening was carried out on IPTG (1 mM), Kanamycin (50 µg/ml), tetracycline (15 µg/ml) and gentamycin (15 µg/ml) containing LB agar plates and incubated for 2 days at 37 °C and the resulting white colonies restreaked. Selected restreaked colonies were incubated overnight in 10 mL of LB media at 37 °C, and the plasmid DNA was extracted using the PureLink™ genomic DNA mini kit (Thermo Scientific). Verification of the bacmid transformation was carried out using colony PCR. PCR reactions were prepared for each restreaked colony to 20 µL containing DNA (1 µL), p55 (5'- CCCAGTCACGACGTTGTAAAACG -3') (0.5 µM), p56 (5'-AGCGGATAACAATTTCACACAGG-3') (0.5 µM), dNTPs (0.2 mM), Phusion enzyme (2 U) and Phusion HF buffer (1x) (Thermo scientific F530S). 1% agarose gel with SYBR-safe DNA gel stain (0.1x) (Thermo Scientific) was used to verify the formation of the bacmid.

Generation of Recombinant N1 Viral Neuraminidase Baculovirus in Sf9

A 60 mL Sf9 (derived from *Spodopetre frugiperda,* IPLB-Sf-21-AE) suspension culture was grown for 2 days at 28 °C in Insect-XPRESS protein-free insect cell medium (Lonza Bioscience) before splitting back to 0.45 x10^6 cells/mL. An adherent cell culture was produced by transferring 2 mL of the suspended cells into each well of the 6-well culture plate and left to incubate in a humidifier chamber at 28 °C until the cells became adherent. A mixture of 1.05 mL Insect-XPRESS media, 50 µL N1 DNA (~100 ng/µl) and 31.5 µL of FuGENE HD transfection reagent (Promega) was created for transfection, and 180 µL added dropwise to each well before placing in the humidified chamber for 3 days. Once the *Sf*9 cells were 95% fluorescent and 90 % viable, the culture was collected and centrifuged at 200 x*g* for 5 min to produce a clarified supernatant, to which 2% fetal bovine serum was added (this is referred to as V1). V1 was amplified into a 50 mL *Sf*9 suspension culture (1 x10^6 cells/mL) with 1 mL of V1 added and incubated at 28 °C and 87 rpm until 95% fluorescence was achieved. The culture was again harvested for centrifugation, and 2% fetal bovine serum was added (this is referred to as V2).

N1 Gene Expression and Purification

The suspended culture of High Five™ (*Trichoplusia ni*, BTI-TN-5BI-4) cells was expanded using Express Five™ SFM supplemented with 20 mM media until the cells reached 3.6 L at 1.2 x10^6 cells/mL using 6 x 600 mL Corning Erlenmeyer shaker flasks. 1.5 mL of the previously prepared V2 was added to each 600 mL and left to incubate at 28 °C and 87 rpm until 95% fluorescence was achieved. Cells were then pelleted at 200 x*g* for 20 min at 4 °C and the clarified media was then further centrifuged for 20 min, 5000 x*g* at 4 °C and then supplemented with cOmplete™ EDTA-free protease inhibitor (Sigma).

Purification of N1

Clarified media was pH adjusted using 150 mL of 50 mM Tris pH 8, 500 mM NaCl, and 20 mM imidazole to a final pH of 7.12. Conditioned media loaded onto a 5 mL HisTrap excel column (GE Healthcare) equilibrated with binding buffer (50 mM Tris pH8, 500 mM NaCl, 20 mM imidazole) and eluted using a buffer gradient 0-100% of elution buffer (50 mM Tris pH 8, 500 mM NaCl, 500 mM imidazole) over 20 CVs at a flow rate of 5 mL/min eluting 1.8 mL fractions. Fractions containing protein were pooled and concentrated using a 30 kDa Vivaspin concentrator to 1 mL. Concentrated protein was loaded onto a pre-equilibrated (25 mM Tris pH 7.5, 150 mM NaCl, 5% glycerol, 2 mM $CaCl_2$) superdex 200 increase 10/300 column (Cytiva). Protein elutions were pooled and concentrated. Concentrated protein was loaded onto an equilibrated 5 mL Mono Q (Cytiva) with 25 mM Tris pH 8, 5% glycerol and 50 mM NaCl, flow rate 1 ml/min. Protein-containing fractions were eluted with 25 mM Tris pH 8, 5% glycerol and 1 M NaCl. Protein elutions were

pooled and concentrated using an Amicron 30K concentrator to 1 ml and a 10 kDa concentrator to 2.7 mg/mL.

<u>*Cryo-EM Grid Preparation and Data Collection*</u>

For enzyme-inhibitor complexes with **8**, **13** and **9**, reaction mixtures were prepared with 1.5 mg/mL of N1 to a final inhibitor concentration of 300 μM in 25 mM Tris, 2 mM $CaCl_2$ pH 7.0 buffer. The mixture was incubated overnight at 37 °C prior to grid preparation. For each reaction mixture, 3 μL was applied to glow-discharged UltraAuFoil (Quantifioil) 300 mesh R1.2/1.3 grids and subsequently blotted for 2 sec with a blot force of -10 using the Mark IV Vitrobot (Thermo Fisher Scientific) at 100 % humidity and 4 °C. Following grid screening on a Glacios, data was collected remotely on a Titan Krios (ThermoFisher Scientific) housed at eBIC Diamond Light Source UK. The microscope operated at 300 kV with a GATAN K3 detector. Each dataset was acquired with a fluence of 18.3 e^-/pixel/s, nominal pixel size of 0.635 Å, and defocus values of -1.4, -1.2, -1.0, -0.8, -0.6 μm; a total fluence of 44 $e^-/Å^2$ was used.

<u>*Image Processing and 3D Reconstruction*</u>

Movie frames for each inhibitor dataset were motion-corrected and damage-weighted using the Relion implementation of MotionCor2(31) to produce micrographs. CTF parameters were estimated with CTFFIND-4.1(32). Using RELION 3(33), automated particle picking was carried out using the Laplacian of Gaussian (LoG) function, and the particles were extracted. These were run into 2D classification and 2D classes with high-resolution features were selected to generate an initial 3D model without symmetry constraints. 3D classification followed. Finally a 3D refinement with C4 symmetry imposed was performed, at which point the projects were imported into RELION 5,(34). Here, CTF-refinement was performed using per-particle estimation, except for astigmatism, which was estimated on a per micrograph basis(33). Finally, Bayesian polishing(35) gave rise to maps at 2.0 Å for compound **8**, 2.0 Å for compound **13**, and 2.3 Å for compound **9**.

<u>*Model Building and Refinement*</u>

UCSF Chimera(36) was used to fit the models [PDB:3TI3](37) into cryo-EM maps and to carefully calibrate pixel size. There were slight differences between 3TI3 and our maps in addition to the introduced point mutations; to account for these, model building was carried out in Coot(38). Models were refined and validated with Phenix(39) using real-space refinement with secondary structure restraints. Structural figures were rendered in Chimera(36).

hNeu2 expression and purification

<u>*h*Neu2 construct sequence</u>

MGSSHHHHHHGTAENLYFQGSMASLPVLQKESVFQSGAHAYRIPALLYLPGQQSLLAFAEQRASKKDEHAELIVLRR
GDYDAPTHQVQWQAQEVVAQARLDGHRSMNPCPLYDAQTGTLFLFFIAIPGQVTEQQQLQTRANVTRLCQVTSTDHG
RTWSSPRDLTDAAIGPAYREWSTFAVGPGHCLQLNDRARSLVVPAYAYRKLHPIQRPIPSAFCFLSHDHGRTWARGH
FVAQDTLECQVAEVETGEQRVVTLNARSHLRARVQAQSTNDGLDFQESQLVKKLVEPPPQGCQGSVISFPSPRSGPG
SPAQWLLYTHPTHSWQRADLGAYLNPRPPAPEAWSEPVLLAKGSCAYSDLQSMGTGPDGSPLFGCLYEANDYEEIVF
LMFTLKQAFPAEYLPQ

A plasmid encoding human neuraminidase 2 was synthesised in a pET-28a vector, which was transformed into BL21 (DE3) Gold cells and grown on Lysogeny Broth (LB) agar plates containing 50 μg mL-1 kanamycin for 16 h at 37 °C. A single colony was picked and used to inoculate LB containing 50 μg mL^{-1} kanamycin. This pre-culture was incubated at 37 °C for 16 h with shaking at 250 rpm, and then used to inoculate 6 L of LBE-5052 auto-induction media containing 50 μg mL^{-1} kanamycin. The culture was incubated at 37 °C, with shaking at 250 rpm, for 18 h. The cells were harvested by centrifugation at 5000 x g for 20 min at 4 °C and the supernatant discarded. The pellet was re-suspended in 50 mL buffer A (20 mM

HEPES pH 7.4, 100 mM NaCl, 30 mM imidazole) containing EDTA-free protease inhibitor cocktail (Roche cOmplete), 40 µg mL^{-1} lysozyme and 250 U benzonase nuclease (Sigma Aldrich). The cells were lysed by sonication for 30 min (30 s on / 30 s off) and soluble protein was isolated by centrifugation at 18000 x g for 40 min at 4 °C. The supernatant was loaded onto a pre-equilibrated HisTrap FF Crude 5 mL column (Cytiva) using an Äkta chromatography system (Cytiva). The column was washed with 5 column volumes (CV) of buffer A, before elution with a gradient of buffer B (20 mM HEPES pH 7.4, 100 mM NaCl, 500 mM imidazole) in buffer A (0-100% B over 20 CV). Neu2 containing fractions were identified by SDS-PAGE, pooled and concentrated to a volume of 4 mL. The pooled protein was cleaved using TEV protease using a 1:50 ratio of TEV to Neu2. The reaction was incubated at 4 °C overnight before loading onto an equilibrated HiTrap Q HP anion exchange column (Cytiva) and elution with a 0-100% gradient of buffer D (20 mM Tris-HCl pH 7.3, 1 M NaCl) in C (20 mM Tris-HCl pH 7.3). Eluted protein was then loaded on an equilibrated HiTrap SP HP cation exchange column (Cytiva) and eluted with a 0-100% gradient of buffer D (50 mM MES pH 6.7, 1 M NaCl) in C (50 mM Tris-HCl pH 6.7). Neu2 containing fractions were identified by SDS-PAGE, pooled and concentrated.

Intact mass spectrometry

40 µM N1 used for cryo-EM structure determination was incubated with 300 µM compound **13** or water overnight at 37 °C in 25 mM Tris, 2 mM $CaCl_2$ pH 7.0. 4x volume of ice-cold acetone was added to the mixture and incubated at -20 °C overnight. The sample was centrifuged for 10 min at 12000 x*g* at 4 °C before removal of the acetone and resuspension in ice-cold methanol with sonication. The sample was spun again then washed once more with methanol. The protein was resuspended in ammonium acetate before loading onto a MALDI-ToF UltrafleXtreme mass spectrometer.

Neuraminidase peptide mapping

To 9 µL of 1 mg/mL N1 (used for cryo-EM structure determination) in 33.3 mM MES pH 6.5, 4 mM $CaCl_2$, 1 µL of 10 mM compound **13** was added and the reaction incubated overnight at 37 °C. 1 µL of 2 M NH_4HCO_3 and 1.2 µL of 10X denaturing reagent (40 mM DTT, 2% SDS) were added and the reaction heated to 80 °C for 5 min and then cooled to room temperature. 5 µL of 0.5 M IAA was added and the reaction incubated at room temperature in the dark for 30 min. The reaction was split between 2 tubes (5 µL each), 0.5 µL of 0.5 µg/µL sequencing grade trypsin or trypsin and chymotrypsin was added and the solution incubated at 37 °C overnight. To the tryptic peptides, 1.5 µL of 1% TFA was added and then 5 µL added to 15 µL 0.1% TFA. 20 µL of the diluted sample was loaded onto EvoTip pure for introduction onto an 8 cm column. A 100SPD pre-set gradient on an EvoSep One UPLC system was used and DDA data were acquired using a Bruker timsToF HT. Data were searched against the sequence of the recombinant N1 construct and common contaminants using the Byonic search engine (Protein Metrics). Variable modifications of oxidation and N terminal acetylation and fixed alkylation of cysteine residues were allowed. An additional variable modification of 370.23 Da (± 1 Da) was allowed as the mass of compound **13**. Data were viewed using the Byonic viewer.

Apparent IC_{50} determination

Apparent half-maximal inhibitory concentration (IC_{50}) values were determined by pre-incubation of inhibitors (25 µL of 4X final concentration) for 30 min (or indicated pre-incubation time) with 25 µL of the enzyme (1 U/mL H1N1 wildtype, 0.6 U/mL H5N1 wildtype, 2 U/mL H1N1 H275Y-mutant, 0.3 U/mL H5N1 H274Y-mutant, or 4 nM H3N2 and B neuraminidases and *h*Neu2 [final concentration 1 nM]) in 33.3 mM 2-(*N*-morpholino)ethanesulfonic acid (MES) buffer containing 4 mM $CaCl_2$, pH 6.5 (or 100 mM MES pH 5 for *h*Neu2) at 37 °C. 50 µL of 200 µM MUNANA was added to each well. The plate was shaken for 10 s and was subsequently incubated at 37 °C. After 2 h, incubation was stopped by addition of Na_2CO_3 (0.2 M aq. soln., 100 µL). Fluorescence of liberated 4-methylumbelliferone was measured at λ_{EX} 366 nm and λ_{EM} 445 nm using an LS-55 fluorimeter (Perkin Elmer). Non-linear regression analysis of normalised blank-corrected data points was used to determine apparent IC_{50} values.

K_M determination

100 µL of MUNANA (500, 250, 125, 62.5, 31.3, 15.6, and 7.8 µM) in assay buffer (33.3 mM MES containing 4 mM $CaCl_2$, pH 6.5) or 100 µL assay buffer (as a blank) was added in triplicate to a black 96 well flat bottom plate. This plate was preincubated for 15 min at 37 °C. At the same time, neuraminidase overexpression lysates (0.5 U/mL H1N1 wildtype, 0.3 U/mL H5N1 wildtype, 1 U/mL H1N1 H275Y-mutant, or 0.15 U/mL H5N1 H274Y-mutant) were incubated at 37 °C. After preincubation, 100 µL of the lysate was added to each well. Directly after adding the enzyme solution, fluorescence product was measured every 60 sec for 30 min using a Clariostar 430-1177 fluorimeter (at 37°C, λ_{EX} = 366 nm, λ_{EM} = 445 nm, shaking at 500 rpm for 8 sec before each measurement). From the triplicate values, the mean value was calculated at each point in time. These triplicate means were duplicated in biological replicate for statistical analysis. Specific activity was obtained from the linear parts of curves (7 to 30 min). Nonlinear regression (Michaelis Menten kinetic analysis) was performed on these rates using GraphPad Prism 8.0 to obtain the K_M values.

Determination of kinetic parameters K_I and k_{inact}

To a black 96 well flat bottom plate was added 150 µL 200 µM MUNANA and 75 µL of solution of inhibitor at different concentrations in 33.3 mM MES buffer, containing 4 mM $CaCl_2$, pH 6.5. This mixture was preincubated at 37 °C for 15 min. At the same time, overexpression lysate containing (active) neuraminidase (1 U/mL H1N1 wildtype, 0.6 U/mL H5N1 wildtype, or 2 U/mL H1N1 H275Y-mutant) was preincubated at 37 °C for 15 min. Inactivated lysate was used as blank (inactivated by incubation at 95 °C for 30 min, using the same concentrations as the active lysate). 75 µL of lysate was added to each well. Directly after of addition of enzyme, the fluorescence was measured every 5 min for a total of 6 h for acylaziridines or measured every min for 30 min for fluorosialoside **3** using a Clariostar 430-1177 fluorescence spectrophotometer (at 37 °C, λ_{EX} = 366 nm, λ_{EM} = 445 nm, shaken at 500 rpm for 8 sec before each measurement). Background was corrected for each timepoint by subtracting output of inactivated enzyme blanks. Curves were fitted (exponential one phase association) using GraphPad Prism 8.0, solving for

$$[P] = \frac{V_0}{k_{obs}}(1 - e^{-k_{obs}t})$$

to derive observed rate constant K_{obs}. K_{obs} values were then plotted against inhibitor concentration and analysed using Michaelis-Menten

$$k_{obs} = \frac{k_{inact}[\mathrm{I}]}{K_I{}^{app} + [\mathrm{I}]}$$

analysis to derive kinact and K_I^{app}. K_I was derived from K_I^{app} by correcting for competition by the substrate using the function:

$$K_I = \frac{K_I{}^{app}}{(1+\frac{[S]}{K_M})}.$$

ConA immobolisation assay

Concanvalin A (Con A) beads were washed three times with washing buffer (0.1 M sodium acetate, 0.1 M NaCl, 1 mM $MgCl_2$, pH 6.0), centrifuged at 2,000 rcf for 2 min after each wash. 25 µL of resuspended beads were incubated with 25 µL of enzyme-inhibitor complex (9 U/mL H5N1 (wild type) lysate with 10 µM inhibitor in 33.3 mM MES buffer with 4 mM $CaCl_2$, pH 6.5) and the samples were incubated at 4 °C for 4 hours with shaking. The samples were centrifuged at 16,000 rcf for 10 min, and the supernatant was discarded. The beads were washed three times with washing buffer. The final wash was either removed immediately, or left shaking for 4 h or overnight at 4 °C. Supernatants were removed after centrifugation at 16000 rcf for 10 minutes, and 100 µL of 100 mM MUNANA were added. The beads further incubated at 37 °C for 2 h with

the fluorogenic substrate and the reaction was stopped by addition of 100 µL 0.2 M Na_2CO_3, and the mixture transferred to a black flat-bottomed 96-well plate. Fluorescence of liberated 4-methylumbelliferone was measured at λ_{EX} 366 nm and λ_{EM} 445 nm using an LS-55 fluorimeter (Perkin Elmer).

***In vitro* ABP labeling**

In vitro labeling on recombinant neuraminidases was performed by incubating 10 µL recombinant neuraminidase (50 U/mL for H1N1WT, 100 U/mL for H5N1WT, 100 U/mL for H1N1Mut or 15 U/mL for H5N1Mut, equivalent to 1-10 pmol of purified active H1N1 enzyme) with or without prior incubation with inhibitors (at indicated concentrations and incubation times) in 33.3 mM MES buffer containing 4 mM $CaCl_2$ (pH 6.5) at 37 °C. Heat inactivated enzyme samples (boiling at 95 °C for 5 min) were used as negative controls. ABP **12** (10 µL, 2 µM) was added to obtain a 1 µM probe concentration, which was incubated overnight at 37 °C. After ABP incubation, samples were denatured with 5x Laemmli buffer (50 % (v/v) 1.0 M Tris−HCl, pH 6.8, 50% (v/v) 100 % glycerol, 10 % (w/v) DTT, 10 % (w/v) SDS, 0.01 % (w/v) bromophenol blue) by boiling for 5 min at 98 °C. Denatured samples were separated by SDS-PAGE using 10% polyacrylamide gels, at 90 V for 30 min and 200 V for 50–70 min. Wet gel slabs were scanned for ABP-emitted fluorescence using a Bio-Rad ChemiDoc MP imager (Bio-Rad, Hercules, CA, USA) using the Cy5 (λ_{EX} = 625 nm, bandpass 30 nm; λ_{EM} = 695 nm, bandpass 55) channel.

Vaccine ABP labeling

Vaccine preparation was diluted into 150 mM McIlvaine buffer pH 6.5, 2 mM $CaCl_2$ to the indicated concentration for a final reaction volume of 10 µL. One sample of undiluted vaccine was boiled for 5 min at 95 °C to inactivate the proteins. To 10 µL of undiluted or diluted vaccine preparation, 1 µL of 100 µM ABP **12** was added and the reaction incubated at 37 °C overnight. 3 µL of 4X Laemmli loading buffer (BioRad) was added to the reaction and the samples were boiled for 5 min at 95 °C. 10 µL of each sample was loaded onto a 12 or 15-well 4-20% gradient polyacrylamide gel (BioRad) and the gel ran for 40 min at 200 V. The gel was then extracted into de-ionised water and then transferred onto a Typhoon 5 laser-scanner (Cytiva) and the Cy5 fluorescence imaged (λ_{EX} 635 nm; λ_{EM} > 665 nm). The gel was placed in Coomassie Brilliant Blue solution and microwaved for 1 min to fix the proteins. After staining, the gel was de-stained in de-ionised water and the Coomassie stained proteins was imaged.

For competition gels, 10 µL of undiluted vaccine preparation was incubated with 1 µL of 1 mM compound **9** at 37 °C for 4 hours. To the reaction, 1 µL of ABP **12** was added and the reactions incubated at 37 °C overnight and then treated as above.

Vaccine quantification gel

1 µL of 10X concentrations of N1 enzyme (from cryo-EM preparation) were diluted into 10 µL of 150 mM McIlvaine buffer pH 6.5, 2 mM $CaCl_2$. To these enzyme dilutions and an undiluted 10 µL vaccine sample, 1 µL of 100 µM ABP **12** was added and the reactions incubated at 37 °C overnight and the samples treated as above. Labeling intensities were measured using imageJ. Each bands intensity was normalised to the intensity of the 2.5 µM band, separately for the three repeats. The mean normalised intensity ± SEM was plotted vs the N1 concentration and a curve fitted using the Padé (1,1) approximant (non-linear regression) module in GraphPad Prism. The equation for this curve was:

$$Y = \frac{A_0 + A_1 \cdot X}{B_1 \cdot X + 1}$$

The normalised intensity for the two bands labeled in the vaccine preparation were plugged into the equation as Y values, with the values for A_0, A_1 and B_1 given by the curve fitting program in GraphPad Prism. The mean ± SEM for X from the equation over the three repeats was plotted on the curve vs the mean of the normalised intensity ± SEM.

Microneutralisation assays

This assay was carried out using a WHO compliant methodology(40, 41). Briefly, MDCK-SIAT1 (H3N2) or MDCK (H1N1) cells were seeded into a 96-well plate (200µL/well) using a 1:20 dilution factor and left to reach confluency at 37 °C, 5% CO_2 over three days. Influenza A (H3N2 or H1N1) virus was added to the washed cells and left at 37°C for 3 hours. 2-fold serial dilutions (100-0.8 nM) of the five chosen inhibitors (**2**, **6**, **8**, **9**, **10**, **13**) were added to the cells, which were then incubated for 4 hours. The inoculum was removed and a 0.6% Avicel overlay containing 2 µg/mL trypsin was added, and cells were incubated overnight at 37°C. The overlay was aspirated from the wells, and the cells were fixed with ice-cold 4% paraformaldehyde in PBS (200 µL/well) for 45 min before washing with PBS. Cells were permeabilised using 0.2% Triton X-100 in PBS for 30 min before washing with PBS. A monoclonal antibody for influenza A nucleoprotein was added to the fixed cells for 1 hour, followed by a 1-hour incubation with a secondary horseradish-peroxidase antibody. True Blue^{TM} was added as a peroxidase substrate, and the reaction was allowed to proceed until blue plaques were observed; the plates were then washed with distilled water to stop the reaction from proceeding before being dried. EC_{50} values were calculated using a custom software which quantifies the percentage of stained cells in a well and compares duplicate dilution average values to the average of the viral control. The EC_{50} value is the closest concentration at which a 50% reduction in stained cells compared to the viral control was observed.

Synthetic Methods

General:

All moisture sensitive reactions were carried out in oven dried glassware under a nitrogen atmosphere. Chemicals were obtained from commercial sources and were used as received. Solvents used in reactions were dried on molecular sieves 4Å or 3Å. Reaction progress was monitored by thin layer chromatography (TLC) on Merck F254 silica TLC-plates visualised by 254 nm UV light and/or spraying with Hanessian's Stain (prepared by dissolving $(NH_4)_6Mo_7O_{24}\bullet 4H_2O$ (25 g/L) and $(NH_4)Ce(SO_4)_4\bullet 2H_2O$ (10 g/L) in 10% aq. H_2SO_4), $KMnO_4$ spray (20 g/L $KMnO_4$ and 10 g/L K_2CO_3 in water) or Ninhydrin (2% ninhydrin in EtOH (w/v)), followed by heating. Column chromatography was carried out on silica gel (60 Å, 40-63 µm), obtained from Screening devices BV. Neutralised silica gel was prepared by portion wise suspending of silica gel (500 g) in H_2O (1.7 L) containing NH_4OH (25% solution, 100 mL) stirring for 30 min, filtering off and drying the residue at 150 °C. NMR spectra were recorded on a Bruker AV-400 or a Bruker AV-500 NMR spectrometer. Chemical shifts (∂) are reported in ppm, relative to tetramethylsilane (TMS) or residual solvent signals. Peaks were assigned using correlation spectroscopy (COSY) and heteronuclear single quantum coherence spectroscopy (HSQC). HRMS spectra were measured on a Thermo Finnigan LTQ Orbitrap mass spectrometer equipped with an electrospray ion source in positive mode (source voltage 3.5 kV, sheath gas flow 10, capillary temperature 275 °C) with resolution R=60.000 at m/z=400 (mass range = 150-4000).

Compound S1

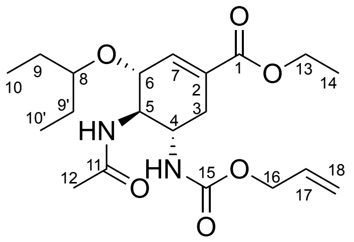

To a solution of Oseltamivir phosphate **2** (4.10 g, 10.0 mmol) in THF (50 mL) and H_2O (50 mL) was added AllocCl (1.59 mL, 15.0 mmol,1.5 eq.) and the reaction mixture was stirred overnight, diluted with H_2O and extracted with DCM (3x). Combined organics were dried over Na_2SO_4 and concentrated *in vacuo.* Column chromatography (0-50% EtOAc in pentane afforded compound **S1** as a white solid (3.86 g, 9.73 mmol, 97%). **^{1}H NMR** (400 MHz, $CDCl_3$) δ 6.78 (dd, J = 3.0, 1.8 Hz, 1H, H-7), 5.97 – 5.81 (m, 2H, H-17, NH), 5.66 (d, J = 8.9 Hz, 1H, NH), 5.33 – 5.14 (m, 2H, H-18), 4.63 – 4.44 (m, 2H, H-16), 4.20 (AB, 2H, H-13), 4.09 (m, 1H, H-5), 4.01 (m, 1H, H-6), 3.84 (m, 1H, H-4), 3.37 (quint , J = 5.7 Hz, 1H, H-8), 2.75 (dd, J = 18, 5.3 Hz, 1H, H-3A), 2.35 (ddt, J = 18.0, 9.2, 2.8 Hz, 1H, H-3B), 1.96 (s, 3H, H-12), 1.57 – 1.43 (m, 4H, H-9, H-9'), 1.28 (t, J = 7.2 Hz, 4H, H-14), 0.88 (m, 6H, H-10, H-10'). **^{13}C NMR** (101 MHz, CDCl3) δ 171.2 (C=O), 166.1 (C=O), 156.7 (C=0), 137.5 (C-7), 132.8 (C-17), 129.4 (C-2), 117.6 (C-18), 82.3 (C-8), 75.5 (C-6), 65.6 (C-16), 61.0 (C-13), 54.1 (C-5), 50.1 (C-4), 30.7 (C-3), 26.3 (C-9), 25.8 (C-9'), 23.3 (C-12), 14.3 (C-14), 9.6 (C-10), 9.4 (C-10'). HRMS (ESI) m/z: calculated for C20H32N2O6 [M+H]+: 397.23331; found 397.23315. **HRMS** (ESI) m/z: calculated for $C_{20}H_{32}N_2O_6$ $[M+H]^+$: 397.23331; found 397.23315.

Compound 4

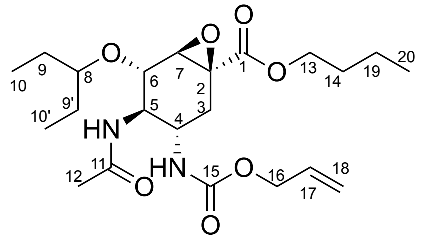

A solution of *t*BuOOH (5.5M solution in decane, 6.0 mL 33 mmol, 16.5 eq.) in THF (20 mL) was cooled to -78 °C. *n*-BuLi (2.5M solution in hexane, 12 mL, 30 mmol, 15 eq.) was added dropwise, causing vigorous gas formation. The reaction mixture was stirred for 10 min at the same temperature. A solution of **S1** (792 mg, 2.0 mmol) in THF (20 mL) was added and reaction mixture was stirred for 6 h while it was allowed to warm up to rt. AcOH (2 mL) was added to quench and the mixture was diluted with DCM, washed with a 2:1 mixture of sat. aq. $Na_2S_2O_3$ and sat. aq. $NaHCO_3$. Combined organic layers were dried over Na_2SO_4 and concentrated *in vacuo*. Column chromatography (0-50% EtOAc in pentane afforded compound **4** as a white solid (332 mg, 0.75 mmol, 38% brsm). **^{1}H NMR** (400 MHz, $CDCl_3$) δ 6.16 – 6.00 (br. d, 1H, NH), 5.94 – 5.79 (m, 1H, H-17), 5.55 (d, J = 9.1 Hz, 1H, NH), 5.31 – 5.14 (m, 2H, H-18), 4.60 – 4.44 (AB, 2H, H-16), 4.21 – 4.05 (m, 2H, H-13), 3.99 – 3.88 (m, 1H, H-5), 3.61 (m, 2H, H-4, H-6), 3.41 (s, 1H, H-7), 3.37 (quint, , J = 5.5 Hz, 1H, H-8), 2.56 – 2.39 (m, 2H, H-3), 1.93 (s, 3H, H-12), 1.64 – 1.58 (m, 2H, H-19), 1.52 (m, 4H, H-9, 9'), 1.36 (m, 2H, H-14), 0.94 – 0.84 (m, 9H, H-10, 10', 20). **^{13}C NMR** (101 MHz, CDCl3) δ 171.0 (C=O), 168.9 (C=O), 156.7 (C=O), 132.8 (C-17), 117.7 (C-18), 82.9 (C-8), 75.1 (C-6), 66.0 (C-16), 65.7 (C-13), 60.4 (C-7), 57.8 (C-2), 53.51 (C-4), 46.5 (C-4), 30.5 (C-3, C-14), 26.1, 25.4 (C-9, C-9'), 23.3 (C-12), 19.2 (C-19), 13.8 (C-20), 9.8, 9.0 (C-10, C-10'). **HRMS** (ESI) m/z: calculated for C20H32N2O6 [M+H]+: 441.25953; found 441.25933. **HRMS** (ESI) m/z: calculated for $C_{20}H_{32}N_2O_6$ $[M+H]^+$: 441.25953; found 441.25933.

Compound 5

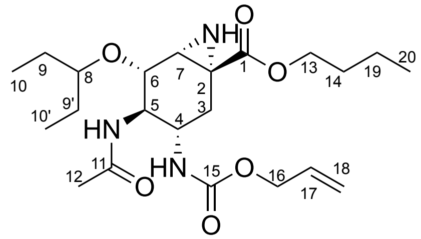

To a solution of epoxide **4** (330 mg, 0.75 mmol) in EtOH (16 mL) and H_2O (4 mL) was added NaN_3 (237 mg, 3.64 mmol, 5 eq.) and NH_4Cl (195 mg, 3.64 mmol, 5 eq.). The reaction mixture was stirred at reflux overnight. Sat aq. $NaHCO_3$ was added and the mixture was extracted with DCM (3x), combined organics were dried over Na_2SO_4 and concentrated *in vacuo*. Column chromatography (20-50% EtOAc in pentane) afforded a mixture of azidoalcohols. To an ice-cooled solution of this mixture of azidoalcohols thus obtained in DCM (10 mL) was added Et_3N (1 mL, 7.2 mmol, 9.6 eq.) and MsCl (0.28 mL, 3.6 mmol, 4.8 eq.). The mixture was stirred for 5 h, diluted with H_2O and extracted three times with DCM. Combined organics were dried over Na_2SO_4 and concentrated *in vacuo.* To an ice-cooled solution of the crude mesylates thus obtained in THF (15 mL) was added PBu_3 (0.27 mL, 1.08 mmol, 1.44 eq.). Following stirring for 2h, H_2O (1.5 mL) and Et_3N (1 mL, 7.16 mmol, 10 eq.) were added and the reaction mixture was stirred overnight and concentrated *in vacuo*. Purification by column chromatography (0-60% EtOAc in pentane) afforded compound **5** as a white solid (85 mg, 0.19 mmol, 26%). **^{1}H NMR** (400 MHz, $CDCl_3$) δ 5.85 (ddd, J = 16.1, 10.5, 5.4 Hz, 1H, H-17), 5.62 (br. d, 1H, NH), 5.43 (d, J = 8.8 Hz 1H, NH), 5.25 (m, 2H, H-18), 4.60 – 4.44 (AB, 1H, H-16), 4.13 (m, 2H, H-13), 4.00 – 3.88 (m, 1H, H-5), 3.70 – 3.53 (m, 2H, H-6, H-4), 3.37 (quint, J = 5.6 Hz, 1H, H-8), 2.98 (dd, J = 14.4, 6.5 Hz,1H, H-3A), 2.75 (br. d, J = 5.9 Hz, 1H, H-7), 1.93 (s, 3H, H-12), 1.87 (dd, J = 14.2, 10.4 Hz, 1H, H-3B), 1.74 (m, 2H, H-14), 1.70 – 1.30 (m, 6H, H-9', H-9, H-19), 0.98 – 0.83 (m, 9H, H-10, H-10', H-20). **^{13}C NMR** (101 MHz, $CDCl_3$) δ 172.5 (C=O), 171.3 (C=O), 156.8 (C=O), 132.9 (C-17), 117.7 (C-18), 82.0 (C-8), 75.8 (C-6), 66.5 (C-16), 65.7 (C-13), 52.7 (C-5), 50.8 (C-4), 43.3 (C-7), 30.6 (C-3, C-14), 26.7, 26.1 (C-9, C-9'), 19.2 (C-19), 13.8 (C-20), 9.8, 9.5 (C-10, C-10'). **HRMS (ESI)** m/z: calculated for $C_{22}H_{37}N_3O_6$ $[M+H]^+$: 440.27551; found 440.27546.

Compound S2

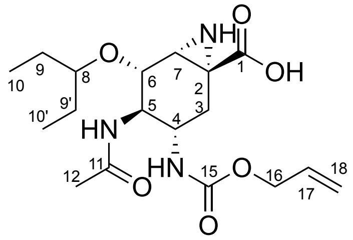

Compound **5** (297 mg, 0.676 mmol) was dissolved in NaOH (0.1M solution in 30:9:1 dioxane:MeOH:H_2O, 20 mL). The reaction mixture was stirred overnight, then a saturated solution of NH_4COOH in MeOH was added until pH 7 and the mixture was concentrated *in vacuo.* Column chromatography (0-20% MeOH in DCM) afforded carboxylic acid **S2** as a white solid (146 mg, 0.381 mmol, 56%). **^{1}H NMR** (400 MHz, D_2O) δ 5.96 – 5.82 (m, 1H, H-17), 5.28 – 5.14 (m, 2H, H-18), 4.54 (ABX, 2H, H-16), 3.90 (m, 2H, H-6), 3.65 (m, 1H, H-5), 3.55 – 3.45 (m, 2H, H-4, H-8), 2.95 (dd, J = 15, 6.2 Hz, 1H, H-3A), 2.82 (d, J = 3.5 Hz, 1H, H-7), 1.93 (s, 3H, H-12), 1.73 – 1.62 (dd, J = 15, 11.6 Hz, 1H, H-3B), 1.57 – 1.45 (m, 4H, H-9', H-9), 0.89 (t, J = 7.2, 3H, H-10), 0.86 (t, J = 7.3 Hz, 3H, H-10'). **^{13}C NMR** (101 MHz, D_2O) δ 177.0 (C=O), 174.3 (C=O), 157.8 (C=O), 132.7 (C-17), 116.9 (C-18), 83.4 (C-8), 75.5 (C-6), 65.5 (C-16), 53.0 (C-5), 49.8 (C-4), 41.7 (C-7), 41.2 (C-2), 29.9 (C-3), 25.8, 25.4 (C-9, C-9'), 22.1 (C-12), 8.7, 8.5 (C-10, C-10'). **HRMS** (ESI) m/z: calculated for $C_{18}H_{29}N_3O_6$ $[M+H]^+$: 384.21291; found 384.21291.

Compound 6

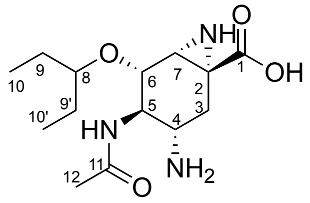

From **S2**:

To a solution of **S2** (78 mg, 0.203 mmol) in DCM (4 mL) and MeOH (4 mL) was added 1,3-dimethylbarbituric acid (95 mg, 0.610 mmol, 3 eq.) and $Pd(PPh_3)_4$ (34 mg, 29 µmol, 0.15 eq.). The reaction mixture was stirred for 2 h and concentrated *in vacuo*. Purification by column chromatography on neutralised silica gel (20-50% MeOH in DCM) afforded amino acid **6** as a white solid (47 mg, 0.16 mmol, 77%).

From **S3**:

A solution of carboxylic acid **S3** (25 mg, 58 µmol) in MeOH (5 mL) was purged with N_2 and a catalytic amount of Pd/C (10% Pd loading) was added. The reaction mixture was then purged with H_2 and left under a flow of H_2 for 5 min and was subsequently stirred under H_2 atmosphere for 5 h, filtered over Celite and the filtrate was concentrated *in vacuo.* Purification by column chromatography using neutralised silica gel (20-50% MeOH in DCM) afforded compound **6** as its ammonium formate salt as a white solid (5.7 mg, 16 mmol, 33%).

1H NMR (400 MHz, D_2O) δ 3.88 (dd, J = 9.5, 3.8 Hz, 1H, H-6), 3.84 (dd, J = 10.5, 9.5 Hz, 1H, H-5), 3.51 (quint, J = 6.1Hz, 1H, H-8), 3.24 (apparent. dt, J = 11.2, 6.8 Hz, 1H, H-4), 3.10 (dd, J = 14.4, 6.6 Hz, 1H, H-3A), 2.77 (d, J = 3.2 Hz, 1H, H-7), 2.01 (s, 3H, H-12), 1.82 (dd, J = 14.4, 11.2 Hz, 1H, H-3B), 1.63 – 1.38 (m, 4H, H-9, H-9'), 0.88 (t, J = 7.5 Hz, 3H, H-10), 0.81 (t, J =7.4 Hz, 3H, H-10'). **13C NMR** (101 MHz, D_2O) δ 177.2 (C=O), 175.1 (C=O), 83.4 (C-8), 75.1 (C-6), 50.7 (C-5), 49.7 (C-4), 41.2 (C-7), 39.9 (C-2), 28.4 (C-3), 25.4, 25.7 (C-9, C-9'), 22.3 (C-12), 8.7, 8.5 (C-10, C-10'). **HRMS** (ESI) m/z: calculated for $C_{14}H_{25}N_3O_4$ $[M+H]^+$: 300.19178; found 300.19158.

Compound 7

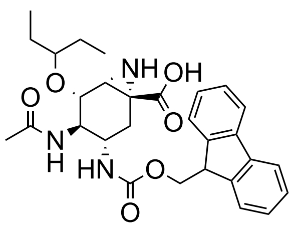

Aziridine **6** (37 mg, 124 µmol) was dissolved in THF (4 mL) and H_2O (2 mL). $NaHCO_3$ (31 mg, 0.37 mmol) and Fmoc-OSu (63 mg, 186 µmol, 1.5 eq.) were added and the reaction mixture was stirred for 2.5 h and concentrated *in vacuo.* Column chromatography on neutralised silica gel (0-20% MeOH in DCM) afforded compound **7** as a white solid (31 mg, 59 µmol, 48%). **HRMS** (ESI) m/z: calculated for $C_{29}H_{35}N_3O_6$ $[M+H]^+$: 522.25986; found 522.25966.

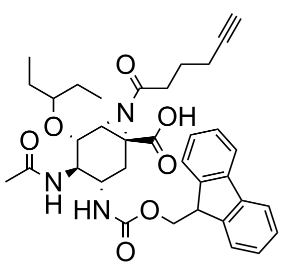

Compound S4

Aziridine **7** (31 mg, 59 µmol) was dissolved in pyridine (1 mL) and cooled to 0 °C. A solution of 1-hexynoic anhydride (37 mg, 178 µmol, 3 eq.) in DCM (1 mL) was added and the reaction mixture was stirred for 2 h. MeOH (2 mL) was added and the reaction mixture was concentrated *in vacuo.* Column chromatography on neutralised silica gel (0-5% MeOH in DCM) afforded compound **S4** as a white solid (27 mg, 44 µmol, 75%). Compound S4 was used without further purification and characterisation for the synthesis of 9. **^{1}H NMR** (500 MHz, DMSO-d_6) δ 7.99 – 7.86 (m, 2H), 7.66 (dd, *J* = 7.8, 5.1 Hz, 2H), 7.56 (d, *J* = 9.4 Hz, 1H), 7.45 – 7.37 (m, 2H), 7.37 – 7.27 (m, 2H), 7.03 (d, *J* = 9.3 Hz, 1H), 4.37 – 4.26 (m, 1H), 4.23 – 4.13 (m, 2H), 4.09 (dd, *J* = 10.2, 7.5 Hz, 1H), 3.67 (q, *J* = 9.9 Hz, 1H), 3.60 (dd, *J* = 9.7, 3.3 Hz, 1H), 3.48 (t, *J* = 5.5 Hz, 1H), 3.05 (dd, *J* = 14.2, 6.8 Hz, 1H), 2.95 (s, 1H), 2.76 (t, *J* = 2.6 Hz, 1H), 2.32 – 2.21 (m, 2H), 2.21 – 2.13 (m, 2H), 1.73 – 1.68 (m, 2H), 1.66 (s, 3H), 1.58 – 1.27 (m, 6H), 0.93 – 0.84 (m, 3H), 0.76 (t, *J* = 7.4 Hz, 3H). **^{13}C NMR** (126 MHz, DMSO-d_6) δ 169.3, 155.9, 143.9, 143.8, 140.6, 127.6, 127.1, 127.0, 125.4, 125.2, 120.1, 120.1, 80.8, 71.4, 65.6, 46.7, 25.8, 24.7, 23.7, 17.6, 9.0, 8.9. **HRMS** (ESI) m/z: calculated for $C_{29}H_{35}N_3O_6[M+H]^+$: 616.30173, found 616.30227.

Compound 9

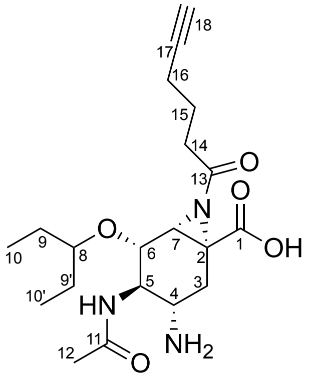

Alkyne **S4** (16 mg, 31 µmol) was dissolved in DMF (0.7 ml), a solution of DBU (0.1 M in DMF, 0.3 mL, 30 µmol, 1 eq.) was added and the reaction mixture was stirred for 30 min and directly submitted to column chromatography on neutralised silica gel. Elution (0-50% MeOH in DCM) afforded compound **9** as a white solid (10 mg, 25 µmol, 83%). **^{1}H NMR** (500 MHz, D_2O) δ 3.83 (dd, J = 9.7, 3.3 Hz, 1H, H-6), 3.68 (t, J = 10.5 Hz, 1H, H-5), 3.57 (quint, J = 5.7 Hz, 1H, H-8), 3.28 (d, J = 3.5 Hz, 1H, H-7), 3.08 (dd, J = 14.5, 6.6 Hz, 1H, H-3A), 2.86 (dt, J = 11.1, 5.6 Hz, 1H, H-4), 2.56 (m, 1H, H-14), 2.34 (m, 1H, H-14), 2.26 (t, J = 7.0 Hz, 2H, H-16), 2.02 (s, 2H, H-12), 1.90 – 1.72 (m, 3H, H-3B, H-15), 1.65-1.42 (m, 4H, H-9, H-9'), 0.90 (t, J = 7.4 Hz, 3H, H-10), 0.81(t, J = 7.4 Hz, 3H, H-10'). **^{13}C NMR** (126 MHz, D_2O) δ 187.16 (C=O), 174.9 (C=O), 173.5 (C=O), 84.1 (C-17), 83.9 (C-8), 75.2 (C-6), 69.8 (C-18), 53.7 (C-5), 49.4 (C-4), 48.3 (C-2), 45.6 (C-7), 35.9 (C-14), 31.4 (C-3), 25.6 (C-9), 24.4 (C-9'), 23.4 (C-15), 22.4 (C-12), 17.2 (C-16), 8.4 (C-10), 8.3 (C-10'). **HRMS** (ESI) m/z: calculated for $C_{35}H_{41}N_3O_7$ $[M+H]^+$: 394.23365, found 394.23374.

Compound 8

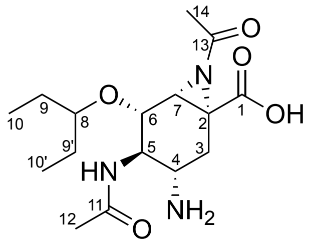

To a solution of aziridine **7** (17 mg, 58 µmol in MeOH (1 mL) and pyridine (1 mL) was added Fmoc-OSu (20 mg, 59 µmol, 1.04 eq.). The reaction mixture was stirred for 2 h and concentrated *in vacuo.* The residue was dissolved in pyridine (1 mL) and Ac_2O (0.015 mL, 136 µmol, 2.4 eq.) was added. The reaction mixture was stirred for 5 h and evaporated *in vacuo.* Column chromatography on neutralised silica gel (0-20% MeOH in DCM) afforded the corresponding acylaziridine. To a solution of the acylaziridine thus obtained in DMF (1.5 mL) was added a solution of DBU (0.1 M in DMF, 0.32 mL, mmol, 1 eq.) and the reaction mixture was stirred for 30 min and directly submitted to column chromatography on neutralised silica gel. Elution (0-50% MeOH in DCM) afforded compound **8** as a white solid (4.05 mg, 12 µmol, 21% over 3 steps). **^{1}H NMR** (500 MHz, D_2O) δ 4.03 (dd, J = 9.7, 3.3 Hz, 1H, H-6), 3.85 (apparent dt, J = 11.8, 6.3 Hz, 1H, H-4), 3.72 (m, 1H, H-5), 3.53 (quint, J = 5.5 Hz, 1H, H-8), 3.22 (d, J = 3.3 Hz, 1H, H-7), 2.98 (dd, J = 15.5, 6.3 Hz, 1H, H-3A), 1.94 (s, 3H, H-14), 1.91 (s, 3H, H-12), 1.82 (dd, J = 15.3, 11 Hz, 1H, H-3B), 1.57 – 1.40 (m, 4H, H-9', H-9), 0.88 (t, J = 7.5 Hz, 3H, H-10), 0.79 (t, J = 7.4 Hz, 3H, H-10'). **^{13}C NMR** (126 MHz, D_2O) δ 74.9 (C=O), 173. (C=O), 173.2 (C=O), 83.4 (C-8), 74.1 (C-6), 52.4 (C-5), 47.67 (C-4), 43.6 (C-2), 42.8 (C-7), 28.3 (C-3), 25.8, 25.3 (C-9, 9'), 21.9 (C-14), 21.8 (C-12), 8.9, 8.3 (C-10, 10'). **HRMS** (ESI) m/z: calculated for $C_{35}H_{41}N_3O_7$ $[M+H]^+$: 342.20235; found 342.20235.

Compound 10

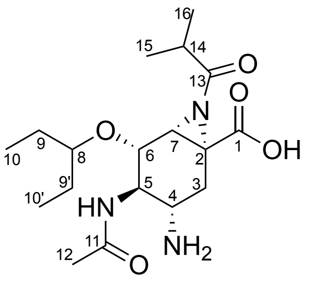

To a solution of aziridine **8** (45 mg, 150 µmol) in MeOH (3 mL) and pyridine (3 mL) was added FmocOSu (82 mg, 240 µmol, 1.6 eq.). The reaction mixture was stirred for 3 h and evaporated *in vacuo.* The residue was dissolved in pyridine and *iso*butyryl anhydride (0.068 mL, 0.451 mmol, 3 eq.) was added. The reaction mixture was stirred for 3.5 h and evaporated *in vacuo.* Column chromatography on neutralised silica gel (0-20% MeOH in DCM) afforded the corresponding acylaziridine. To a solution of this acylaziridine thus obtained in DMF (1 mL) was added DBU (0.1M in DMF, 0.95 mL, 95 µmol, 1 eq.) and the reaction mixture was stirred for 30 min and directly submitted to column

chromatography on neutralised silica gel. Elution (0-50% MeOH in DCM) afforded compound **10** as a white solid (8 mg, 22 µmol, 14% over 3 steps). **^{1}H NMR** (500 MHz, D2O) δ 3.92 – 3.82 (m, 1H, H-6), 3.80 (m, 1H, H-5), 3.61 (t, *J* = 5.6 Hz, 1H, H-8), 3.31 (d, *J* = 3.2 Hz, 1H, H-7), 3.16 (dd, *J* = 14.4, 6.7 Hz, 1H, H-3), 3.11 – 3.02 (m, 1H, H-4), 2.65 – 2.53 (m, 1H, H-14), 2.04 (s, 3H, H-12), 2.03 – 1.91 (m, 1H, H-3'), 1.69 – 1.37 (m, 4H, H-9, 9'), 1.20 (d, *J* = 7.1 Hz, 2H, H-16), 1.07 (d, *J* = 6.8 Hz, 2H, H-15), 0.96 – 0.75 (m, 6H, H-10, H-10'). **^{13}C NMR** (126 MHz, D_2O) δ 192.30 (C=O), 175.01 (C=O), 173.48 (C=O), 83.66 (C-8), 74.90 (C-6), 52.48 (C-5), 49.47 (C-4), 48.02 (C-2), 44.74 (C-7), 36.65 (C-14), 30.39 (C-3), 25.53, 24.34 (C-9, C-9'), 22.43 (C-12), 19.98 (C-16), 17.59 (C-15), 8.32, 8.28 (C-10, C-10'). **^{1}H NMR** (500 MHz, D_2O) δ 3.88 (dd, J = 9.6, 3.3 Hz, 1H, H-6), 3.80 (d, J = 11, 9.5 Hz 1H, H-5), 3.61 (quint, J = 5.6 Hz, 1H, H-8), 3.31 (d, J = 3.2 Hz, 1H, H-7), 3.16 (dd, J = 14.4, 6.7 Hz, 1H, H-3A), 3.07 (m, 1H, H-4), 2.59 (sept, J = 6.7 Hz, 1H, H-14), 2.04 (s, 3H, H-12), 1.95 (dd, J = 14.0, 11.0 Hz, 1H, H-3B), 1.69 – 1.37 (m, 4H, H-9, 9'), 1.20 (d, J = 7.1 Hz, 2H, H-16), 1.07 (d, J = 6.7 Hz, 2H, H-15), 0.93 (t, J = 7.4 Hz 3H, H-10t), 0.83 (t, J = 7.3 Hz, 3H, H-10'). **^{13}C NMR** (126 MHz, D_2O) δ 192.3 (C=O), 175.0 (C=O), 173.5 (C=O), 83.7 (C-8), 74.9 (C-6), 52.5 (C-5), 49.5 (C-4), 48.0 (C-2), 44.7 (C-7), 36.7 (C-14), 30.4 (C-3), 25.5, 24.3 (C-9, C-9'), 22.4 (C-12), 20.0,17.6 (C-15, C-16), 8.3, 8.3 (C-10, C-10'). **HRMS** (ESI) m/z: calculated for $C_{18}H_{31}N_3O_5$ $[M+H]^+$: 370.23365; found 370.23337.

Compound 12

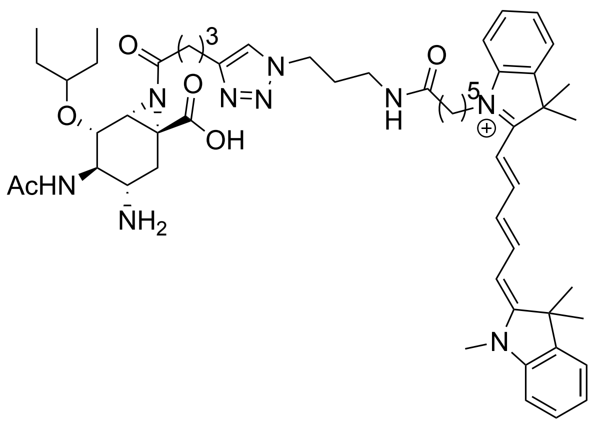

To a solution of alkyne **9** (1.1 mg, 2.8 µmol) and Cy5 azide **11** (1.7 mg, 2.8 µmol, 1 eq.) in DMF (0.6 mL) were added $CuSO_4$ (0.01 M aq. solution, 1 mL, 10 µmol, 3.6 eq.) sodium ascorbate (2.0 mg, 10 µmol 3.6 eq.) and the reaction mixture was stirred overnight. Upon analysis by LCMS, conversion was showed to be incomplete, and a mixture of sodium ascorbate (1.0 mg, 5.0 µmol, 1.8 eq.) and $CuSO_4$ (1.0 mg, 4.0 µmol, 1.4 eq.) was added. The reaction mixture was stirred for an additional 2h and concentrated *in vacuo.* Column chromatography on neutralised silica gel (0-100% MeOH in DCM) afforded compound **12** as a blue solid (2.04 mg, 2.05 µmol, 73%). **HRMS** (ESI) m/z: calculated for $C_{55}H_{76}N_9O_6$ $[M]^+$: 958.59131, found 958.13013.

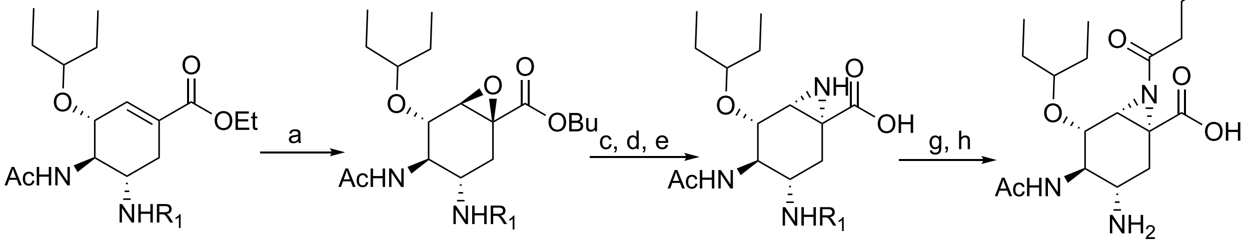

b — **2** R_1=H → **S5** R_1=Cbz

S6

f — **2** R_1=H → **S3** R_1=Cbz

13

Scheme S1. **Reagents and conditions**: (a) CbzCl, $NaHCO_3$, THF, H_2O, 3h, 96%; (b) *t*BuOOH, *n*-BuLi, 6h, 26%; (c) NaN_3, NH_4Cl, EtOH; (d) MsCl, Et_3N, o.n.; (e) PPh_3, THF, then Et_3N H_2O, o.n. 32%; (f) NaOH, MeOH, Dioxane, H2O, 2h, 60%; (g) butyryl anhydride, pyridine, DCM 1h; (h) Pd/C, H_2, MeOH 17% over 2 steps.

Compound S5

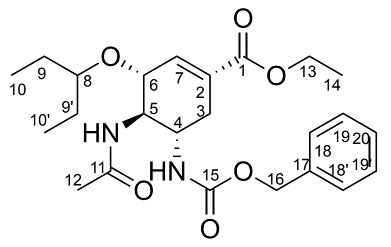

To an ice-cooled solution of Oseltamivir phosphate (**2**, 1.24 g, 3.00 mmol) in THF (50 mL) and H_2O (50 mL) were added $NaHCO_3$ (1.26 g, 15 mmol, 5 eq.) CbzCl (0.57 mL, 4.00 mmol, 1.3 eq.) and the reaction mixture was stirred for 3 h, diluted with H_2O and extracted with DCM (3x). Combined organics were dried over Na_2SO_4 and concentrated *in vacuo.* Column chromatography (0-50% EtOAc in pentane) afforded compound **S5** as a white solid (1.29 g, 2.89 mmol, 96%). **^{1}H NMR** (400 MHz, $CDCl_3$) δ 7.37 – 7.24 (m, 5H, H-18, 18', 19, 19', 20), 6.78 (br. s, 1H, H-7), 5.98 (br. m, 1H, NH), 5.72 (br. m, 1H, NH), 5.06 (AB, J = 12.6 Hz , 2H, H-16), 4.20 (m, 2H, H-13), 4.16 – 3.94 (m, 2H, H-5, H-6), 3.86 (dq, J = 9.5, 5.3 Hz, 1H, H-4), 3.36 (quint, J = 5.7 Hz, 1H, H-8), 2.76 (dd, J = 17.7, 5.2 Hz, 1H, H-3A), 2.35 (ddt, J = 17.9, 9.3, 2.8 Hz, 1H, H-3B), 1.84 (s, 3H, H-12), 1.48 (m, 4H, H-9, H-9'), 1.29 (t, J = 7.1 Hz, 3H, H-14), 0.88 (m, 6H, H-10, H-10'). **^{13}C NMR** (101 MHz, $CDCl_3$) δ 171.2 (C=O), 166.1 (C=O), 156.8 (C=O), 137.4 (C-7), 136.6 (C-17), 129.5 (C-2), 128.6 (C-20), 128.6, 128.2, 128.0 (C-18, C-18', C-19, C-19'), 82.3 (C-8), 75.5 (C-6), 66.8 (C-16), 61.1 (C-13), 54.1 (C-5), 50.1 (C-4), 30.8 (C-3), 26.3, 25.9 (C-9, C-9'), 23.2 (C-12), 14.3 (C-14), 9.6, 9.4 (C-10, C-10'). **HRMS** (ESI) m/z: calculated for $C_{24}H_{34}N_2O_6$ $[M+H]^+$: 447.24896; found 447.24878.

Compound S6

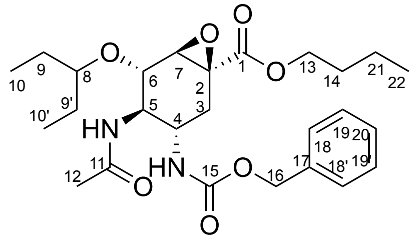

A solution of *t*BuOOH (5.5M solution in decane, 6 mL, 33 mmol, 16.5 eq.) in THF (20 mL) was cooled to -78 °C. *n*-BuLi (2.5 M solution in hexane, 12 mL, 30 mmol, 15 eq.) was added dropwise, causing vigorous gas formation. The reaction mixture was stirred for 10 min at the same temperature and a solution of **S5** (893 mg, 2.00 mmol) in THF (10 mL) was added and the reaction mixture was stirred for 6 h while it was allowed to warm up to rt. AcOH (2 mL) was added to quench and the mixture was diluted with DCM and washed with 2:1 mixture of sat. aq. $Na_2S_2O_3$ and sat. aq. $NaHCO_3$. The combined organic layers were dried over Na_2SO_4 and concentrated *in vacuo*. Column chromatography (0-50% EtOAc in pentane afforded compounds **S6** as a white solid (332 mg, 0.75 mmol, 26%). **^{1}H NMR** (400 MHz, $CDCl_3$) δ 7.54 – 7.27 (m, 5H, H-18,18',19,19', 20), 5.95 (br. m, 1H, NH), 5.53 (br. m, 1H, NH), 5.27 – 4.72 (AB, J = 12.4, 2H, H-16), 4.31 – 4.05 (m, 2H, H-13), 3.99 – 3.84 (m, 1H, H-5), 3.66 (m, 1H, H-4), 3.6 (m, 1H, H-6), 3.42 (br. s, 1H, H-7), 3.36 (quint, J = 5.7 Hz, 1H, H-8), 2.47 (m, 2H, H-3), 1.81 (s, 3H, H-12), 1.67 – 1.45 (m, 8H, H-21, H-14, H-9, H-9'), 0.99 – 0.79 (m, 9H, H-10, H-10', H-22). **^{13}C NMR** (101 MHz, $CDCl_3$) δ 171.1 (C=O), 169.0 (C=O), 156.89 (C=O), 136.7 (C-17), 128.7, 128.3, 128.2 (C-18, 18',19,19', 20), 83.1 (C-8), 75.2 (C-6), 66.9 (C-16), 66.2 (C-13), 60.6 (C-5), 57.9 (C-2), 53.7 (C-4), 46.7 (C-7), 30.6 (C-21), 30.2 (C-3), 26.2 (C-9), 25.5 (C-9'), 23.3 (C-12), 19.3 (C-14), 13.9 (C-22), 10.0, 9.2 (C-10, C-10'). **HRMS** (ESI) m/z: calculated for $C_{26}H_{38}N_2O_7$ $[M+H]^+$: 491.27518; found 491.27488.

Compound S7

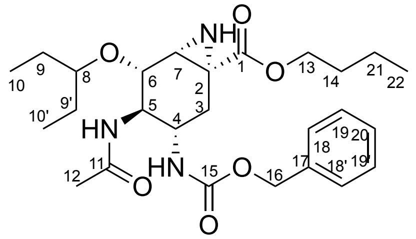

To a solution of epoxide **S6** (117 mg, 0.262 mmol) in EtOH (8 mL) and H_2O (2 mL) was added NaN_3 (85 mg, 1.31 mmol, 5 eq.) and NH_4Cl (70 mg, 1.31 mmol, 5 eq). The reaction mixture was stirred at reflux overnight. Sat aq. $NaHCO_3$ was added and the mixture was extracted with DCM (3x), combined organics were dried over Na_2SO_4 and concentrated *in vacuo*. Column chromatography (20-50% EtOAc in pentane) afforded a mixture of azidoalcohols. To an ice-cooled solution of the mixture of azidoalcohols thus obtained (111 mg, 0.220 mmol) in DCM (5 mL) were added Et_3N (0.192 mL, 0.660 mmol, 3 eq.) and MsCl (68 µL, 0.44 mmol, 2 eq.). The mixture was stirred overnight, diluted with H_2O and extracted three times with DCM, combined organics were dried over Na_2SO_4 and concentrated *in vacuo.* The crude mesylates were used in the next step without further purification. To an ice-cooled solution of these mesylates in THF (5 mL) was added PPh_3 (115 mg, 0.439 mmol, 2 eq.). After stirring for 2h, H_2O (0.5 mL) and Et_3N (0.31 mL, 2.22 mmol, 10 eq) were added and the reaction mixture was stirred overnight and concentrated *in vacuo.* Purification by column chromatography (0-60% EtOAc in pentane) afforded compound **S7** as a white solid (38 mg, 82 µmol, 32%). **^{1}H NMR** (400 MHz, $CDCl_3$) δ 7.39-7.23 (m, 5H, H-18, H-18', H-19, H-19', H-20), 5.08 (br. m, 1H, NH), 5.04 – 4.97 (br. m, 1H, NH), 5.04 (AB, J = 12.7 Hz, 2H, H-16), 4.18 (m, 2H, H-13), 3.94 (m, J = 18.4, 9.7 Hz, 1H, H-5), 3.65 - 3.54 (m, 2H, H-4, H-6), 3.40(br. s, 1H, H-7), 3.35 (quint, J = 5.5 Hz, 1H, H-8), 2.48 (m, 2H, H-3), 1.79 (s, 3H, H-12),1.69 – 1.15 (m, 8H, H-21, H-14, H-9, H-9'), 0.98 – 0.78 (m, 9H, H-10, H-10', H-22). **^{13}C NMR** (101 MHz, $CDCl_3$) δ 171.0 (C=O), 168.9 (C=O), 156.8 (C=O), 136.6 (C-17), 128.6, 128.2, 128.1 (C-18, C-18', C-19, C-19', C-20), 83.0 (C-8), 75.1 (C-6), 66.8 (C-16), 66.0 (C-13), 60.4 (C-7), 57.8 (C-2), 53.6 (C-5), 46.6 (C-4), 30.5 (C-3), 30.1 (21), 26.1 (C-9), 25.4 (C-9'), 23.2 (C-12), 19.2 (C-14), 13.8 (C-22), 9.8, 9.0 (C-10, C-10'). **HRMS** (ESI) m/z: calculated for $C_{26}H_{39}N_3O_6$ $[M+H]^+$ 490.29116, found 490.29129.

Compound S3

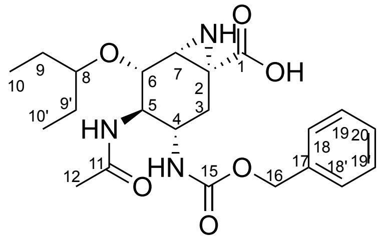

Butyl ester **S7** (81 mg, 0.165 mmol) was dissolved in a NaOH (0.1 M solution in 30:9:1 dioxane: MeOH: H_2O, 5 mL). The reaction mixture was stirred for 2 h, then a saturated solution of NH_4COOH in MeOH was added until pH 7 and the mixture was concentrated *in vacuo.* Column chromatography (0-20% MeOH in DCM) afforded carboxylic acid **S3** as a white solid (43 mg, 99 µmol, 60%). **^{1}H NMR** (400 MHz, MeOD) δ 7.36 – 7.26 (m, 5H, H-18, H-18', H-19, H-19', H-20), 5.1 (AB, J = 12.4 Hz, 2H, H-16), 3.86 – 3.70 (m, 2H, H-5, H-6), 3.55 (m, 1H, H-4), 3.43 (quint, J = 5.7 Hz, 1H, H-8), 3.07 (dd, J = 14.3, 6.2 Hz, 1H, H-3A), 2.74 (d, J = 3.4 1H, H-7), 1.76 (s, 3H, H-12), 1.64 (dd, J = 14.4, 11.8 Hz, 1H, H-3B), 1.57 – 1.50 (m, 4H, H-9, H-9'), 0.95 (t, J = 7.2 Hz, 3H, H-10), 0.86 (t, J = 7.0 Hz, 3H, H-10'). **^{13}C NMR** (101 MHz, MeOD) δ 182.2 (C=O, small peak), 173.7 (C=O), 158.4 (C=O), 138.6 (C-20), 129.5 (C-18, C-18'), 128.9 (C-19, C-19'), 128.7 (C-17), 83.1 (C-8), 77.2 (C-6), 67.2 (C-16), 54.0 (C-5), 51.6 (C-4), 43.3 (C-7), 31.6 (C-2), 27.7 (C-3), 27.0 (C-9, C-9'), 22.9 (C-12), 10.0, 9.7 (C-10, C-10'). **HRMS** (ESI) m/z: calculated for $C_{22}H_{31}N_3O_6$ $[M+H]^+$ 433.21258, found 433.21245.

Compound 13

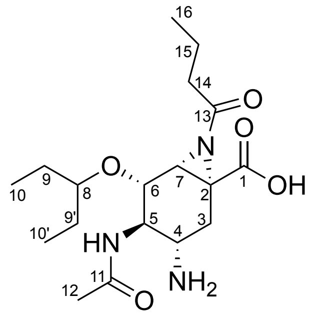

Carboxylic acid **S3** (34 mg, 78 µmol) was dissolved in pyridine (1.5 mL) and cooled to 0 °C. Butyryl anhydride (64 µL, 0.39 mmol, 5 eq.) was added and the reaction mixture was stirred for 1h. MeOH was added and the reaction mixture was concentrated *in vacuo*. Column chromatography on neutralised silica gel (0-10% MeOH in DCM) afforded the crude butyryl aziridine. This crude aziridine in MeOH (3 mL) was purged with N_2 and a catalytic amount of Pd/C (10% Pd loading) was added. The reaction mixture was then purged with H_2 and left under a flow of H_2 for 5 min and was subsequently stirred under H_2 atmosphere for 2.5 h. Solids were filtered off over Celite and the filtrate was concentrated *in vacuo* and purified by column chromatography to afford aziridine **13** (5 mg, 14 µmol, 17%) as a white solid**.**

1H NMR (850 MHz, D_2O) δ 4.01 – 3.96 (m, 2H, H-5, H-6), 3.64 (quint, J = 4.9 Hz, 1H, H-8), 3.39 (d, J=2.6 ,1H, H-7), 3.37 (m , 1H, H-4), 3.26 (dd, J = 14.6, 6.9 Hz, 1H, H-3A), 2.42 (m, 1H, H-14), 2.36 (m, 1H, H-14'), 2.10 (dd, 1H, J=11.0, 14.6, H-3B), 2.06 (s, 3H, H-12), 1.70 – 1.49 (m, 6H, H-15, H-9, H-9'), 0.97 – 0.91 (m, 6H, H-10, H-10'), 0.86 (t, J = 7.4 Hz, 3H, H-16). **13C NMR** (214 MHz, D_2O) δ 187 .8 (C-13), 175.1 (C-11), 172.5 (C-1), 83.9 (C-8), 74.3 (C-6), 50.5 (C-5), 49.3 (C-4), 47.3 (C-2), 45.2 (C-7), 38.9 (C-14), 28.5 (C-3), 25.5 (C-9), 24.3 (C-9'), 22.4 (C-12), 18.0 (C-15), 13.0 (C-16), 8.3, 8.2 (C-10', C-10). **HRMS** (ESI) m/z: calculated for $C_{18}H_{29}N_3O_6$ $[M+H]^+$: 370.23365; found 370.2365.

NMR Spectra

Compound **S1**

^{1}H-NMR $CDCl_3$, 400 MHz

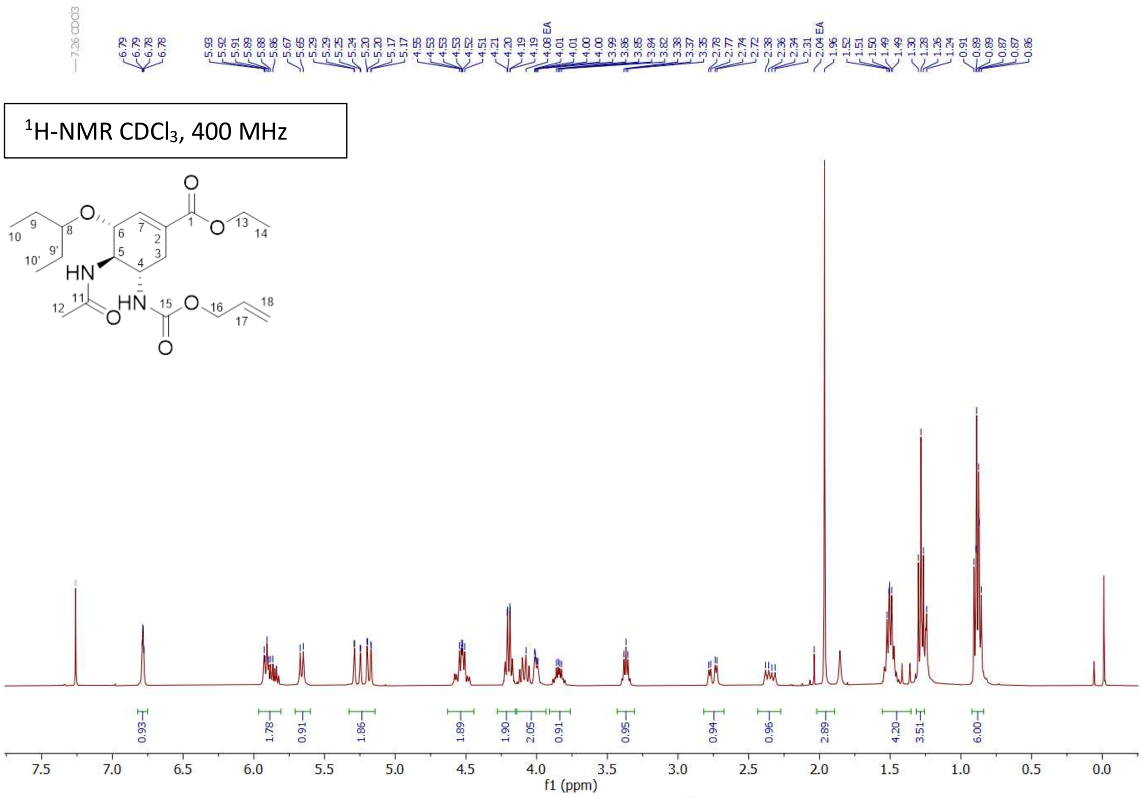

^{13}C-NMR $CDCl_3$, 101 MHz

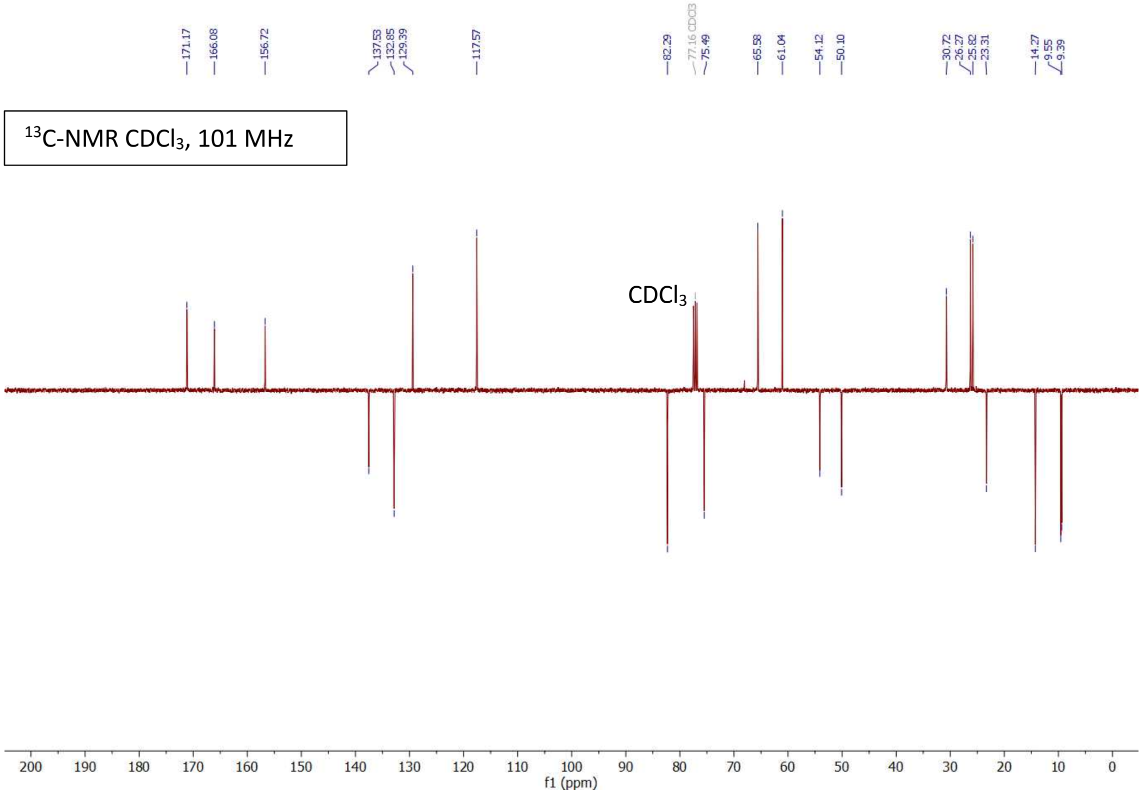

H-H COSY-NMR $CDCl_3$, 101

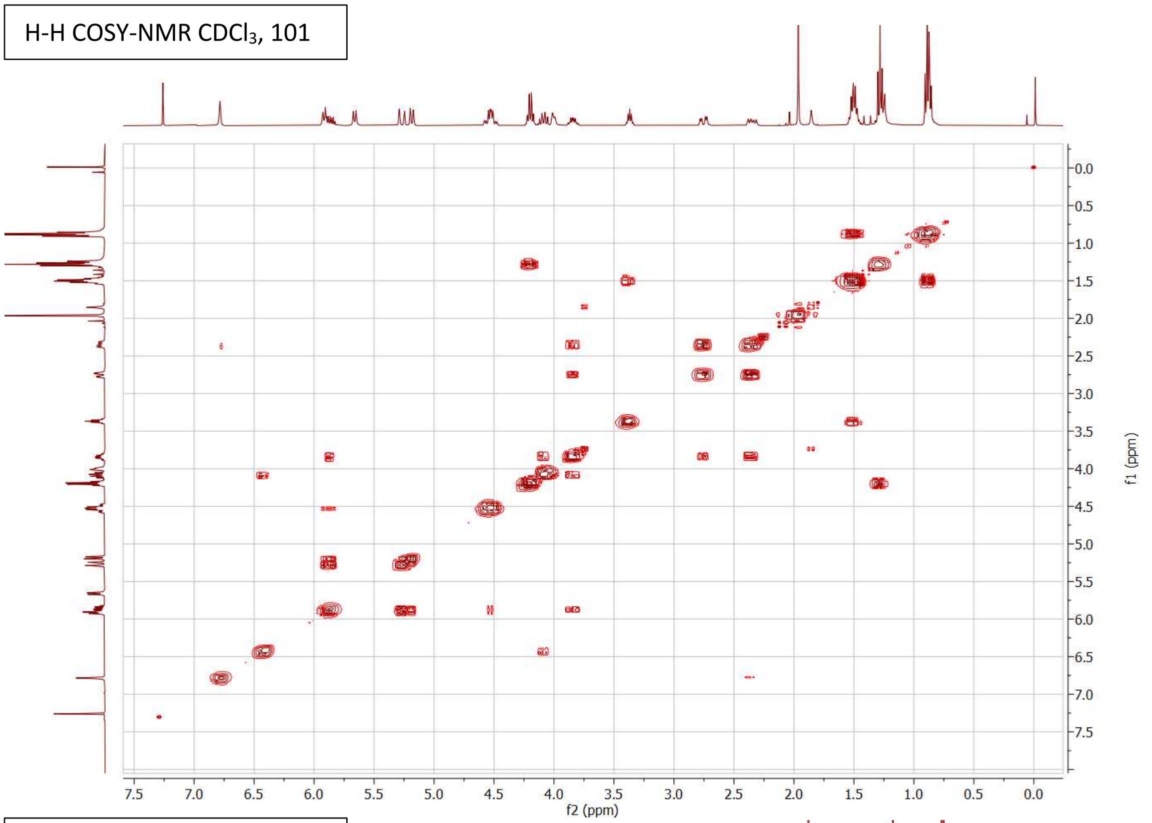

H-C HSQC-NMR $CDCl_3$, 101

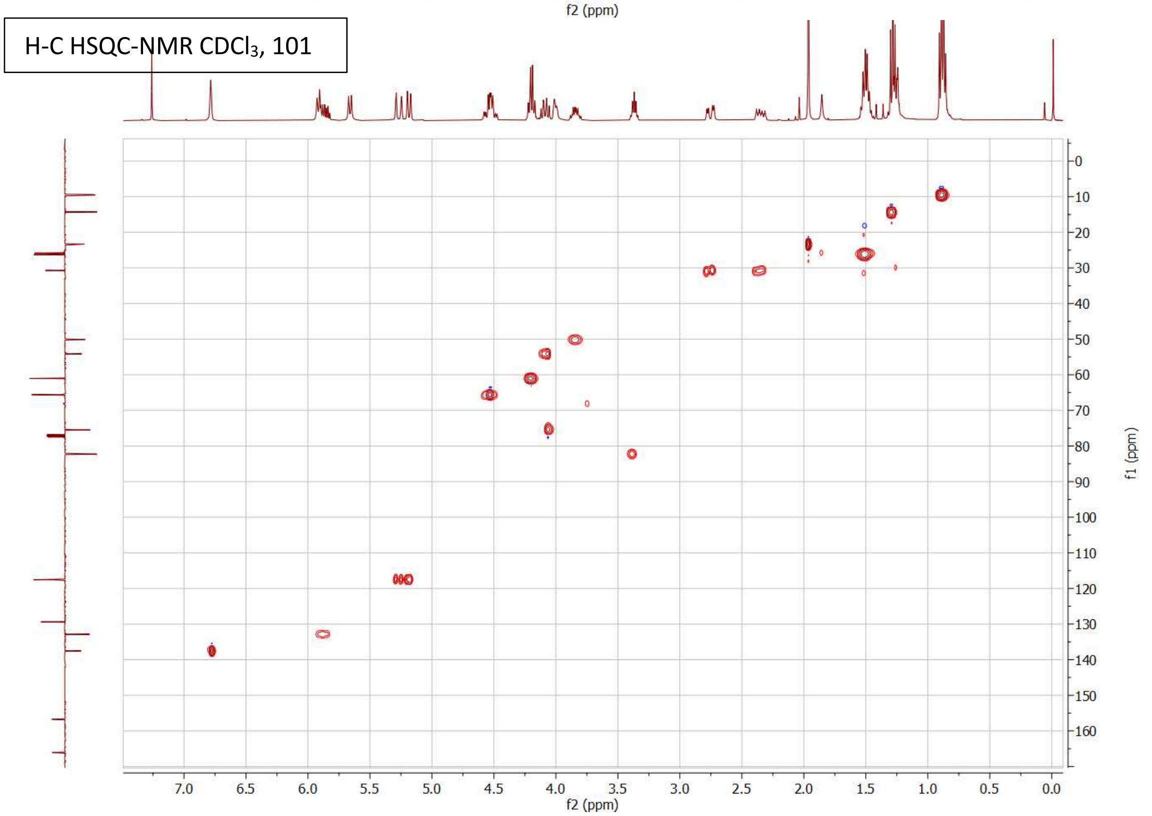

Compound **4**

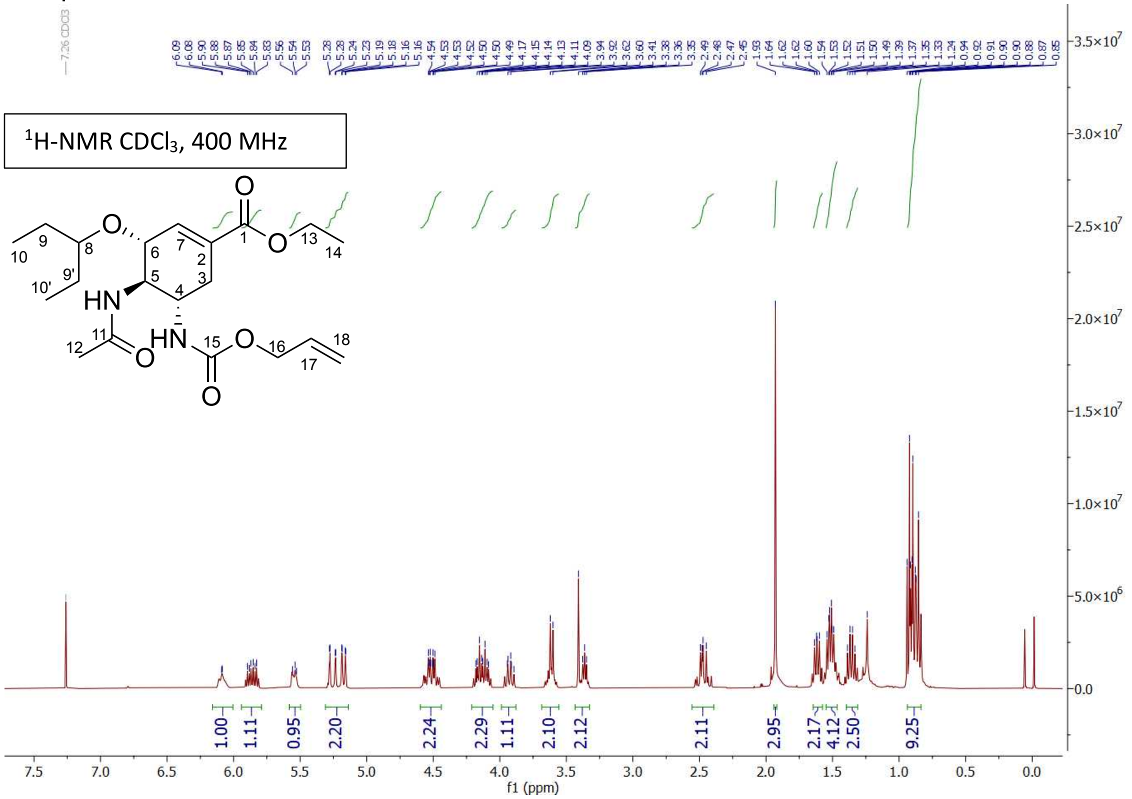

¹H-NMR CDCl₃, 400 MHz
1.00
1.11
0.95
2.20
2.24
2.29
1.11
2.10
2.12
2.11
2.95
2.17
4.12
2.50
9.25
f1 (ppm)

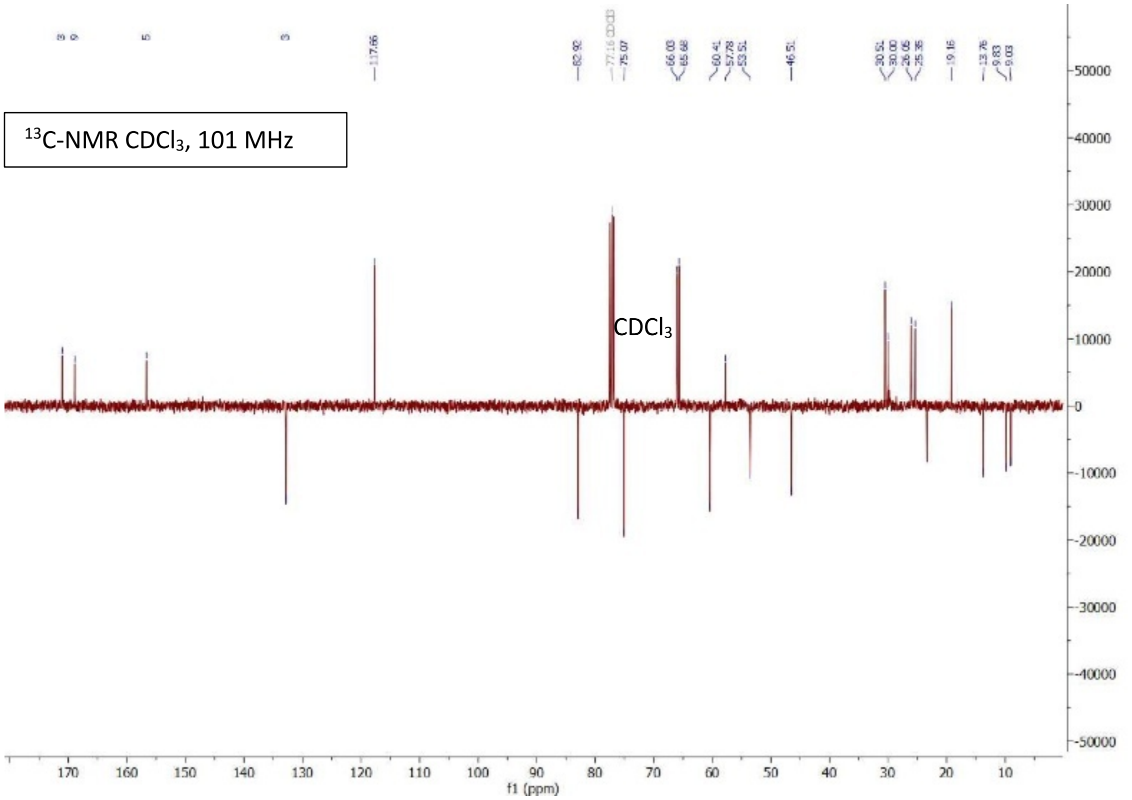

¹³C-NMR CDCl₃, 101 MHz
CDCl₃
f1 (ppm)

H-H COSY-NMR $CDCl_3$, 101

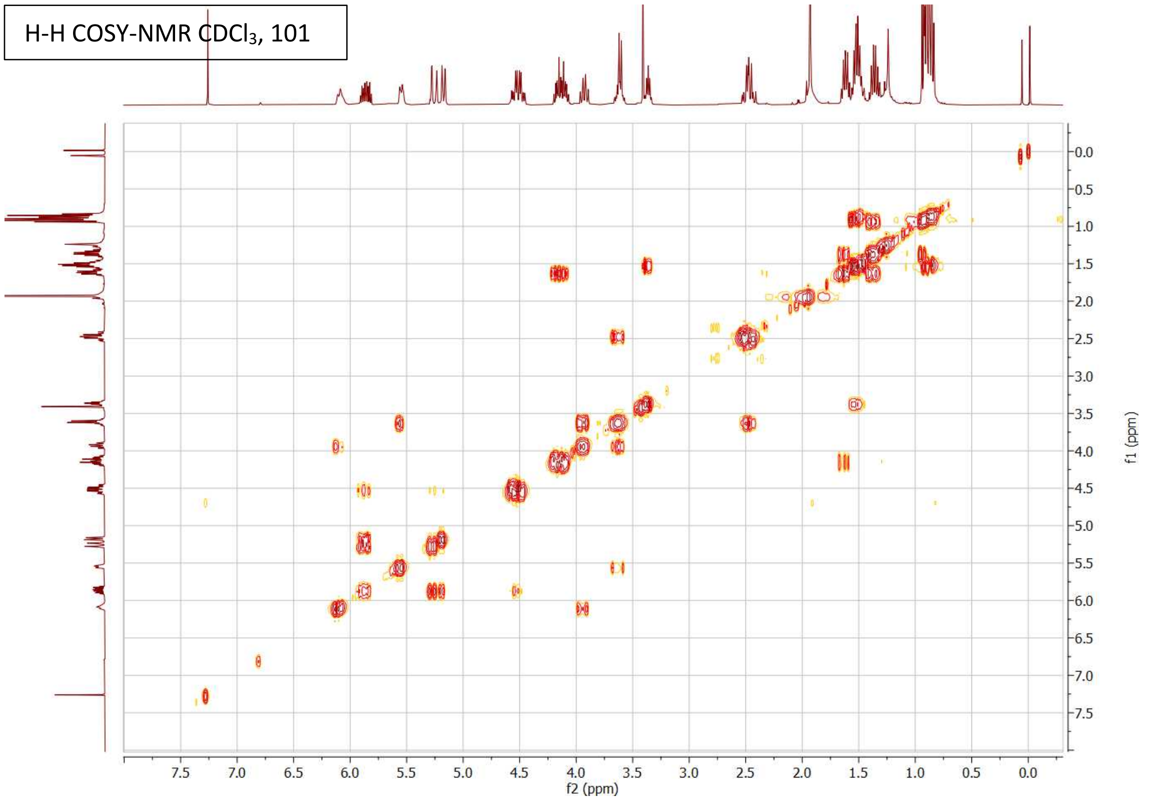

H-C HSQC-NMR $CDCl_3$, 101

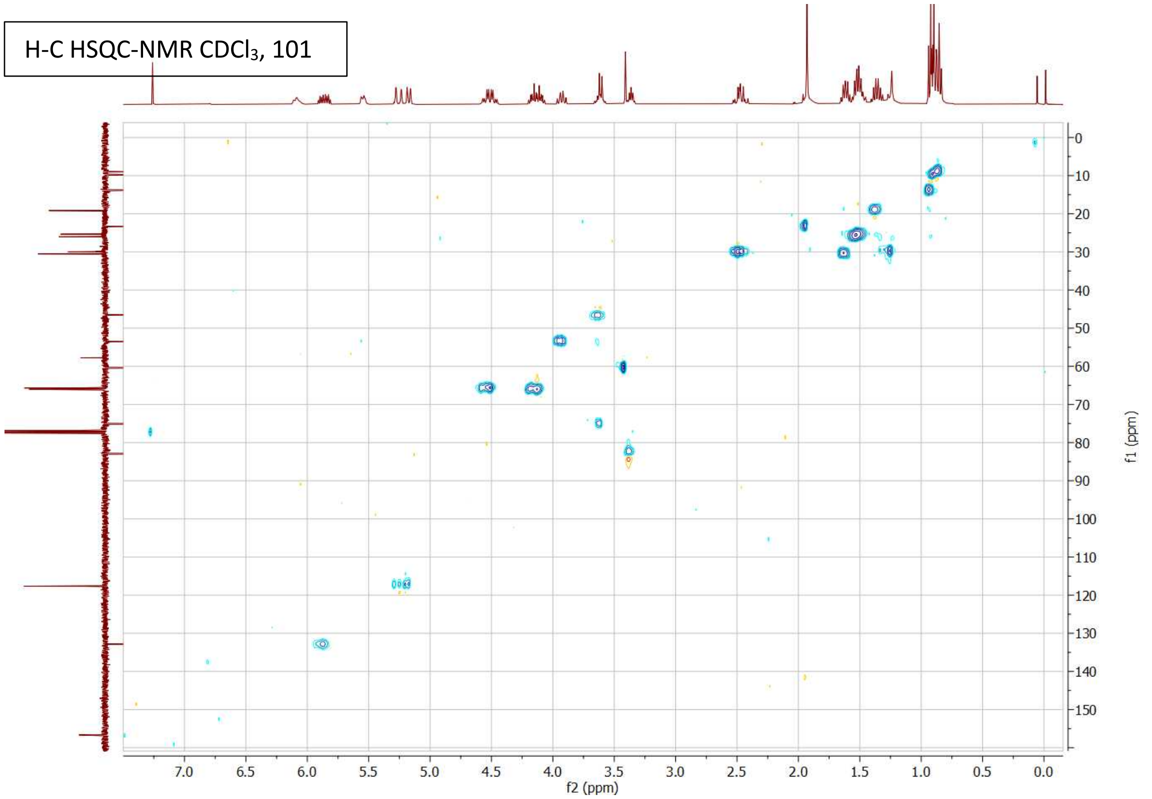

Compound **5**

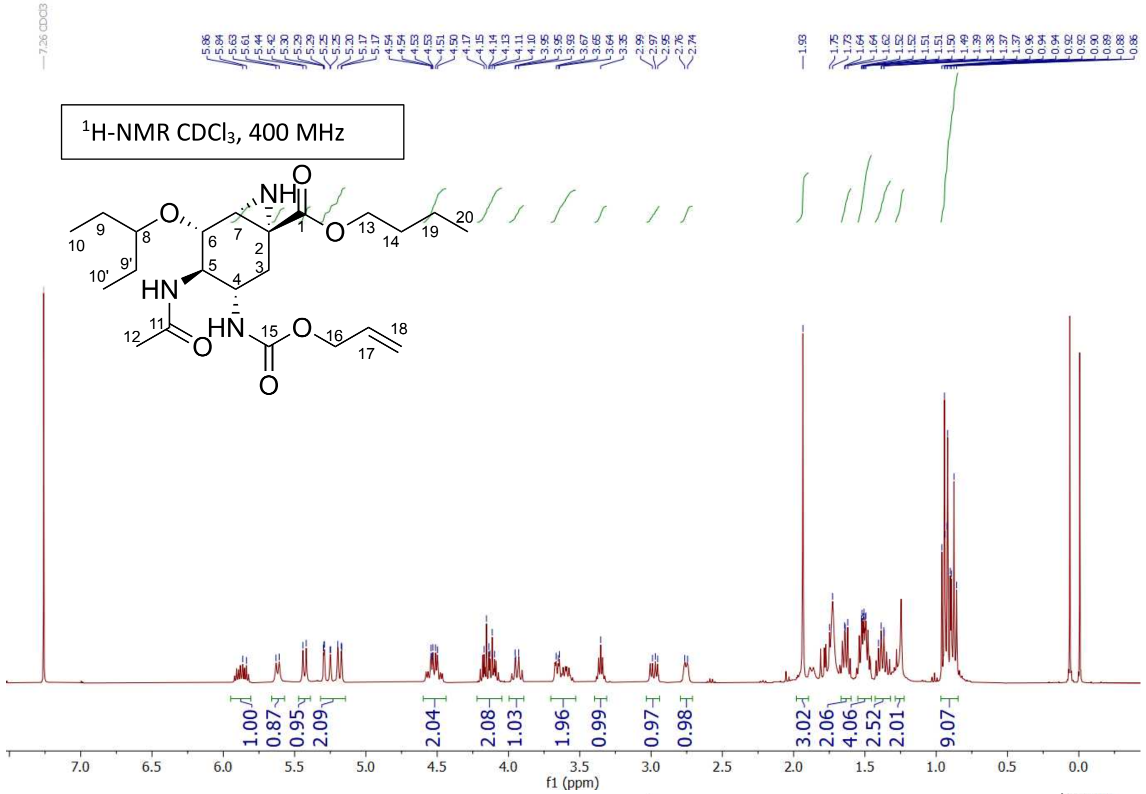

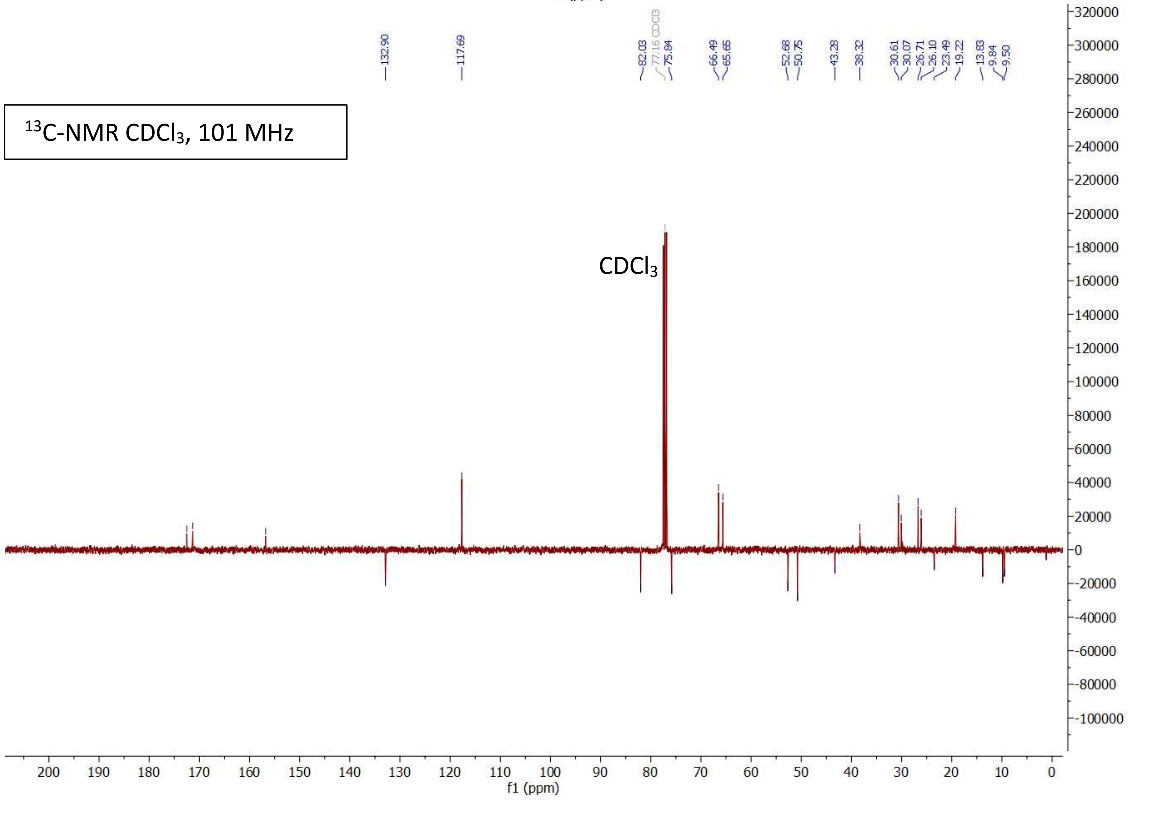

H-H COSY-NMR $CDCl_3$, 101

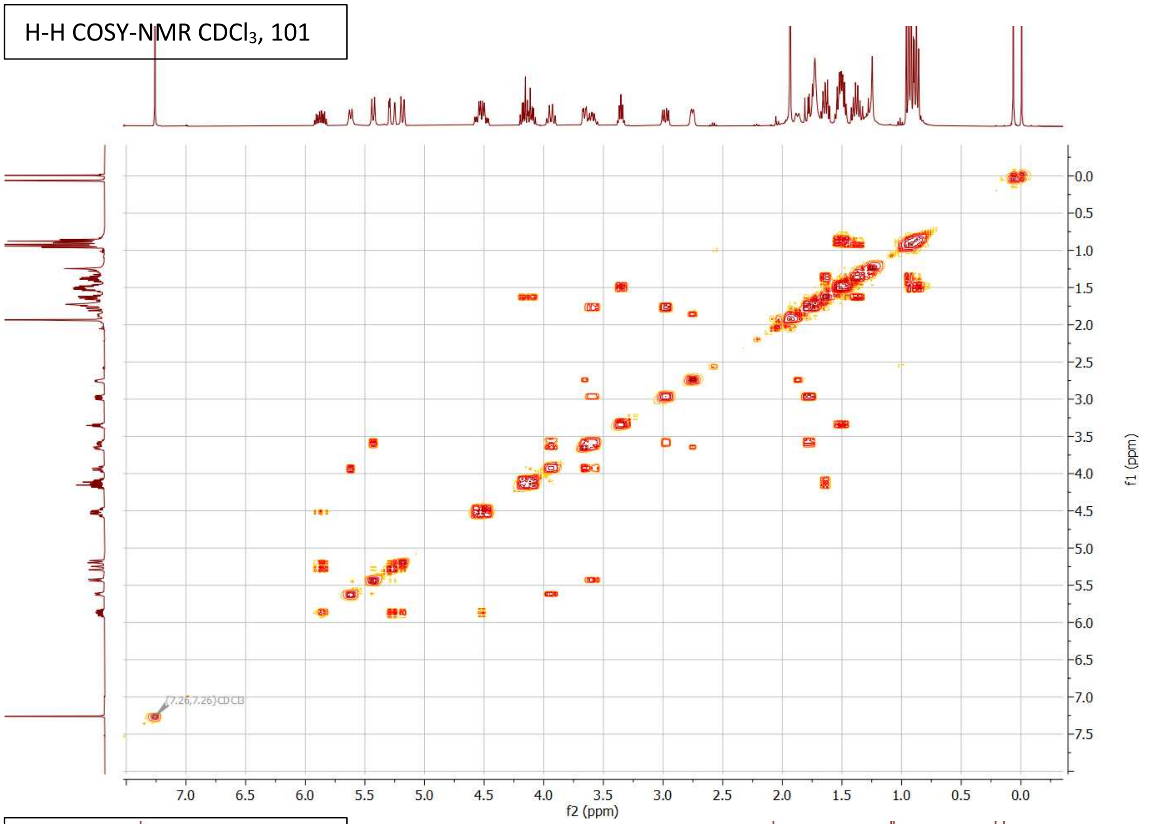

H-C HSQC-NMR $CDCl_3$, 101

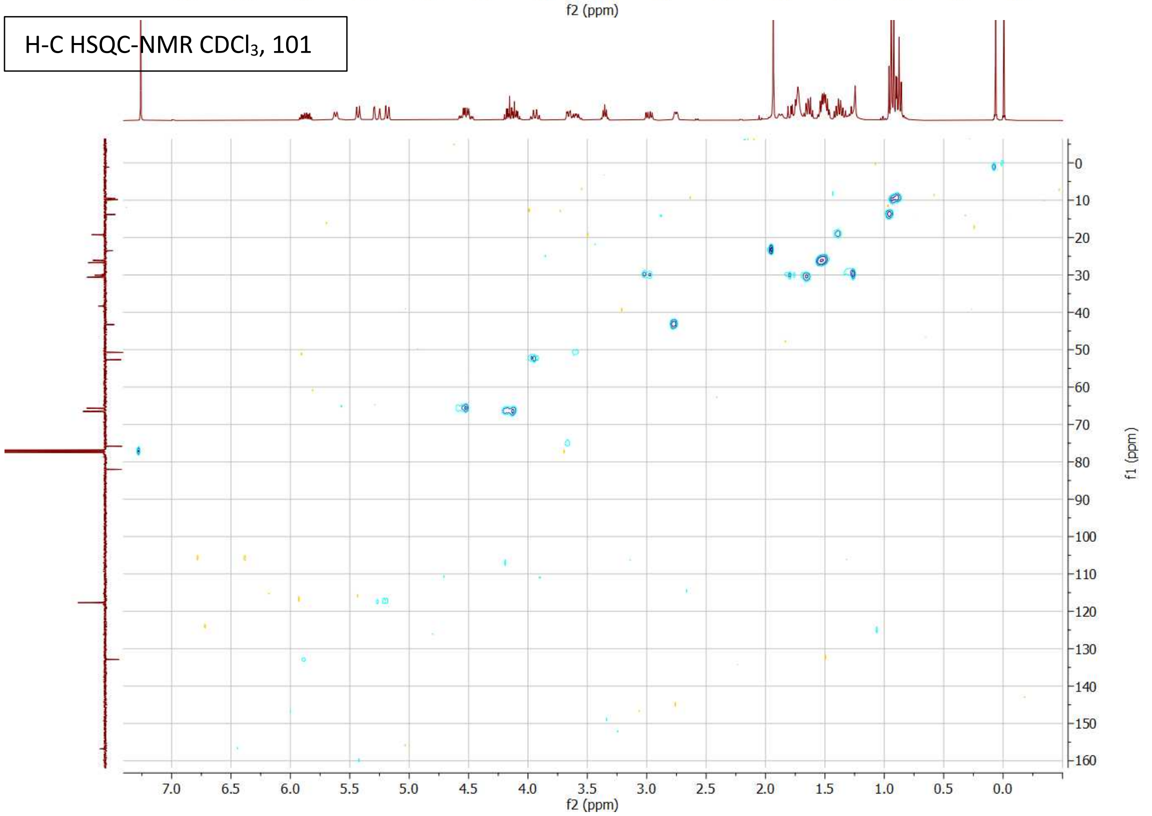

Compound **S2**

^{1}H-NMR D_2O, 400 MHz

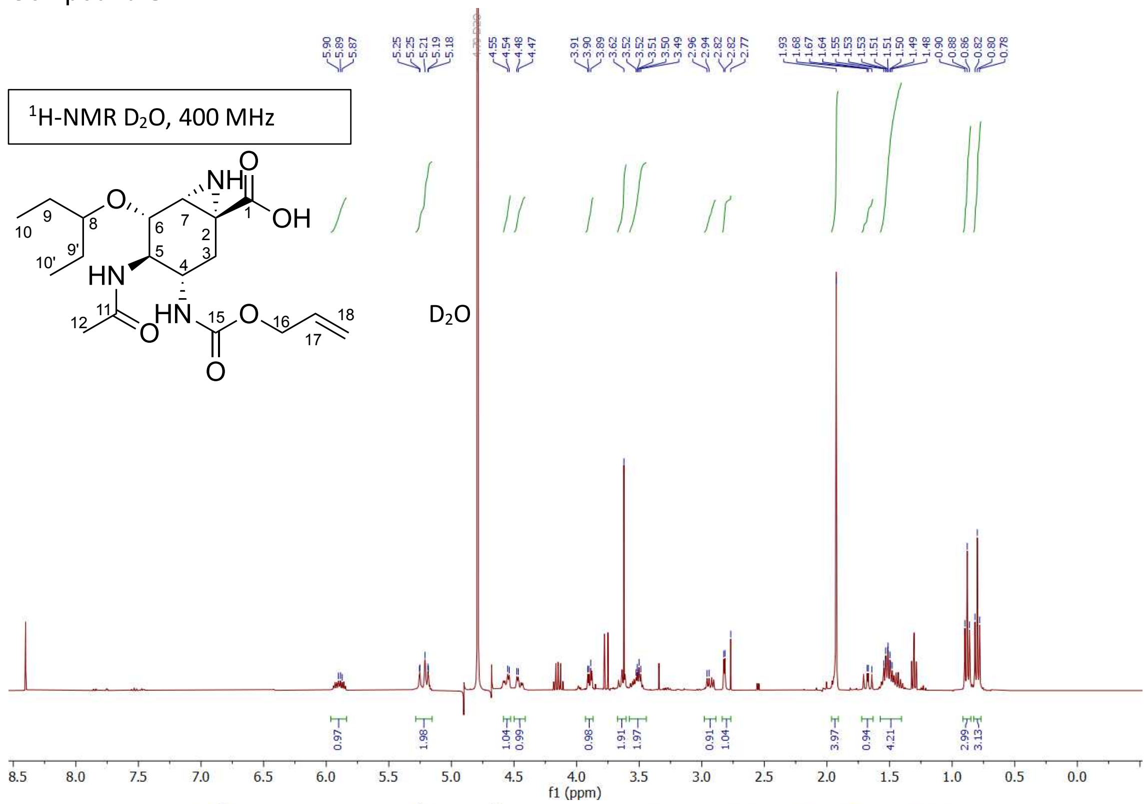

^{13}C-NMR D_2O, 101 MHz

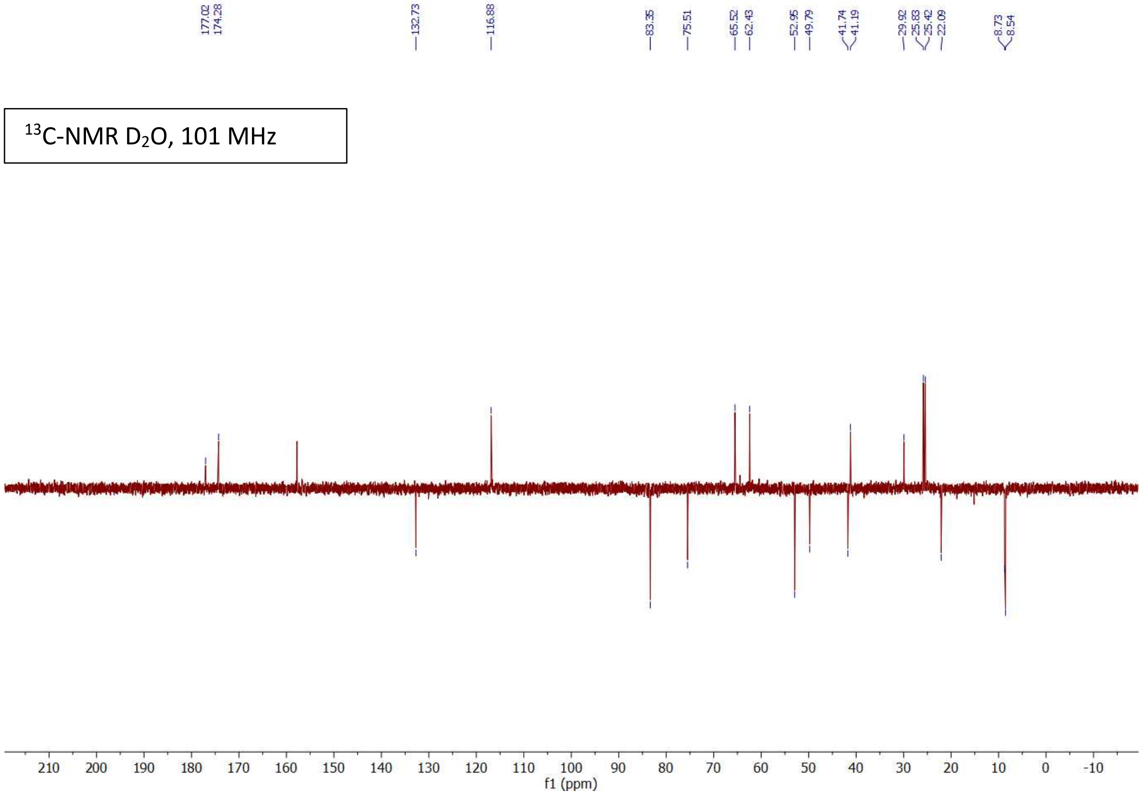

H-H COSY-NMR D_2O, 101

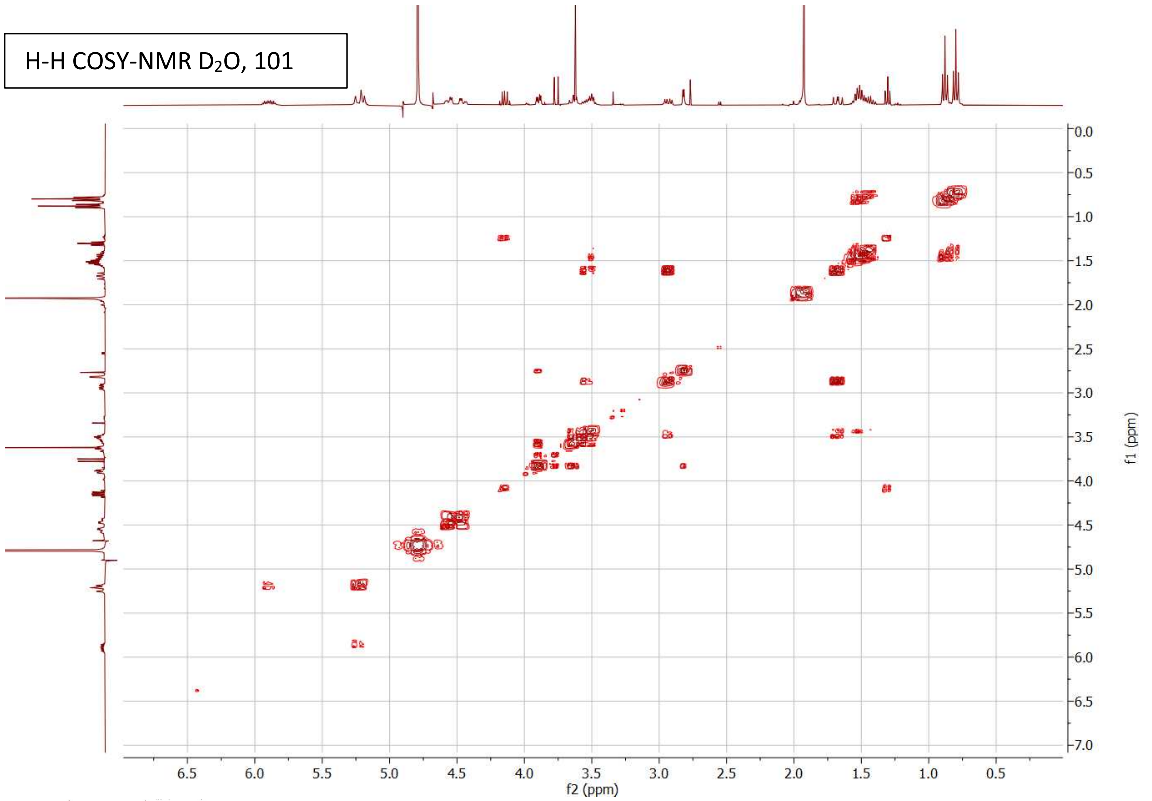

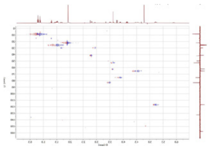

Compound **6**

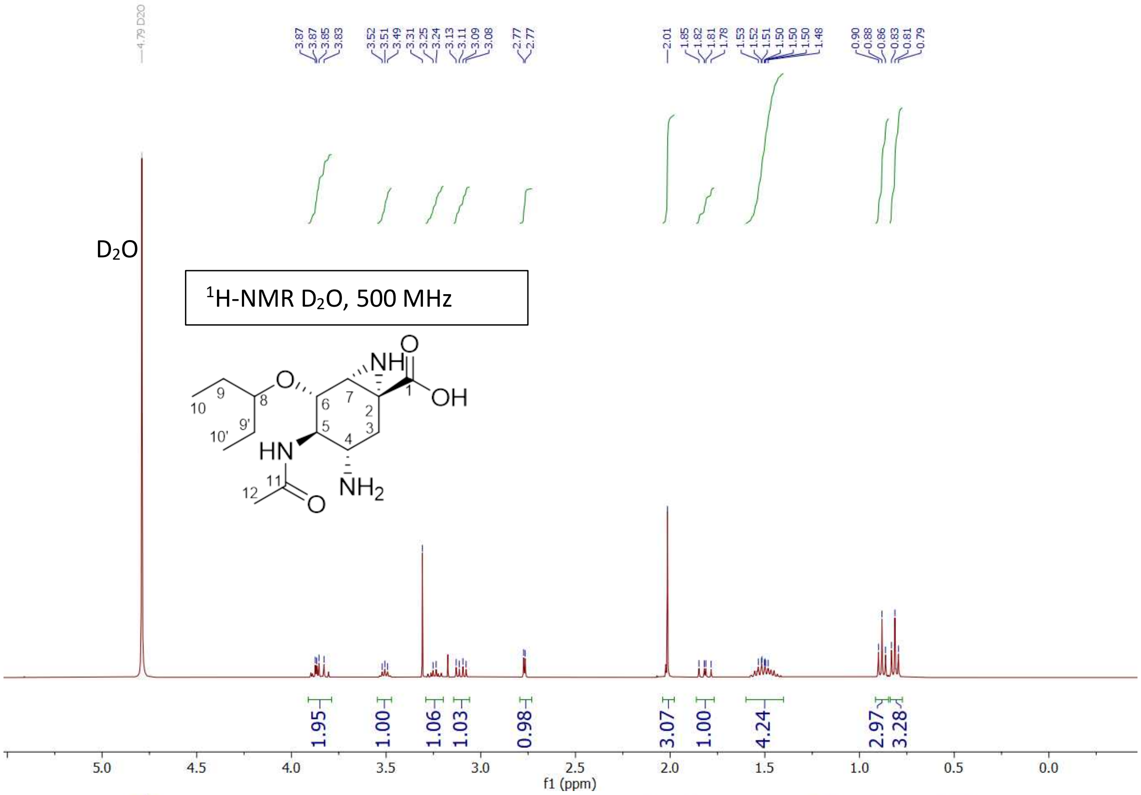
D2O
1H-NMR D2O, 500 MHz
NH
O
OH
HN
NH2
f1 (ppm)

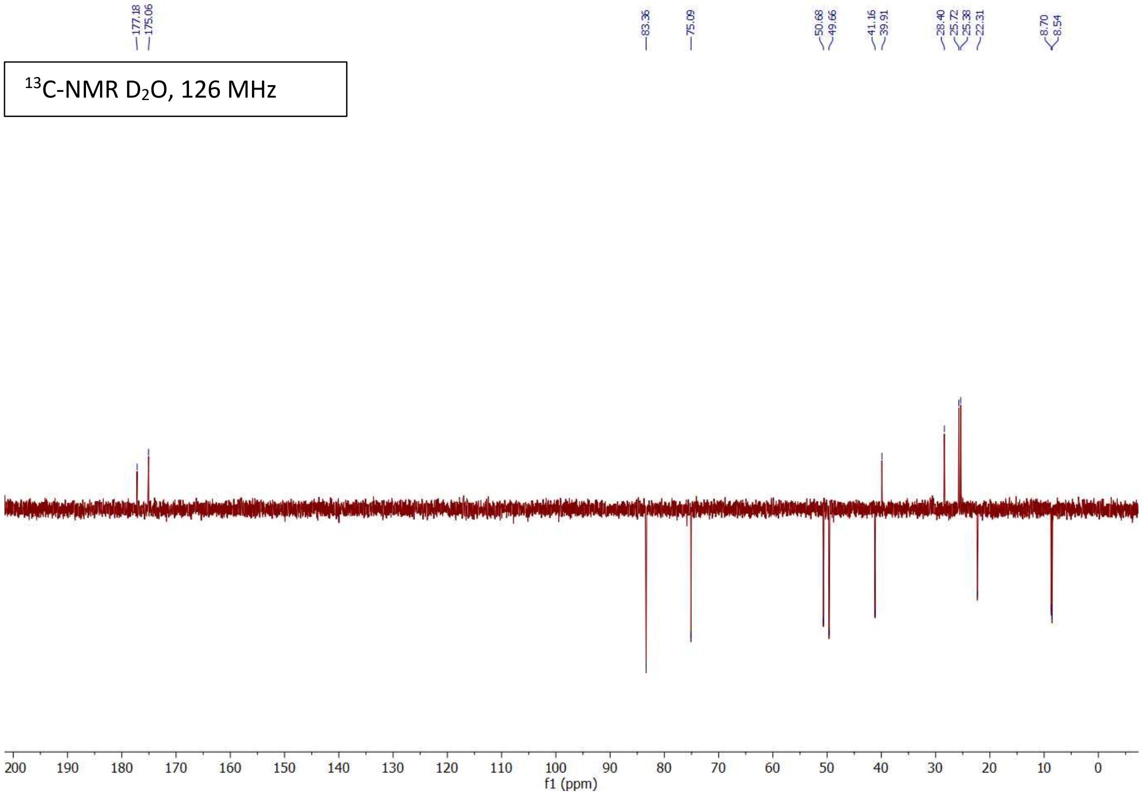
13C-NMR D2O, 126 MHz
f1 (ppm)

Compound **6** (ammonium formate)

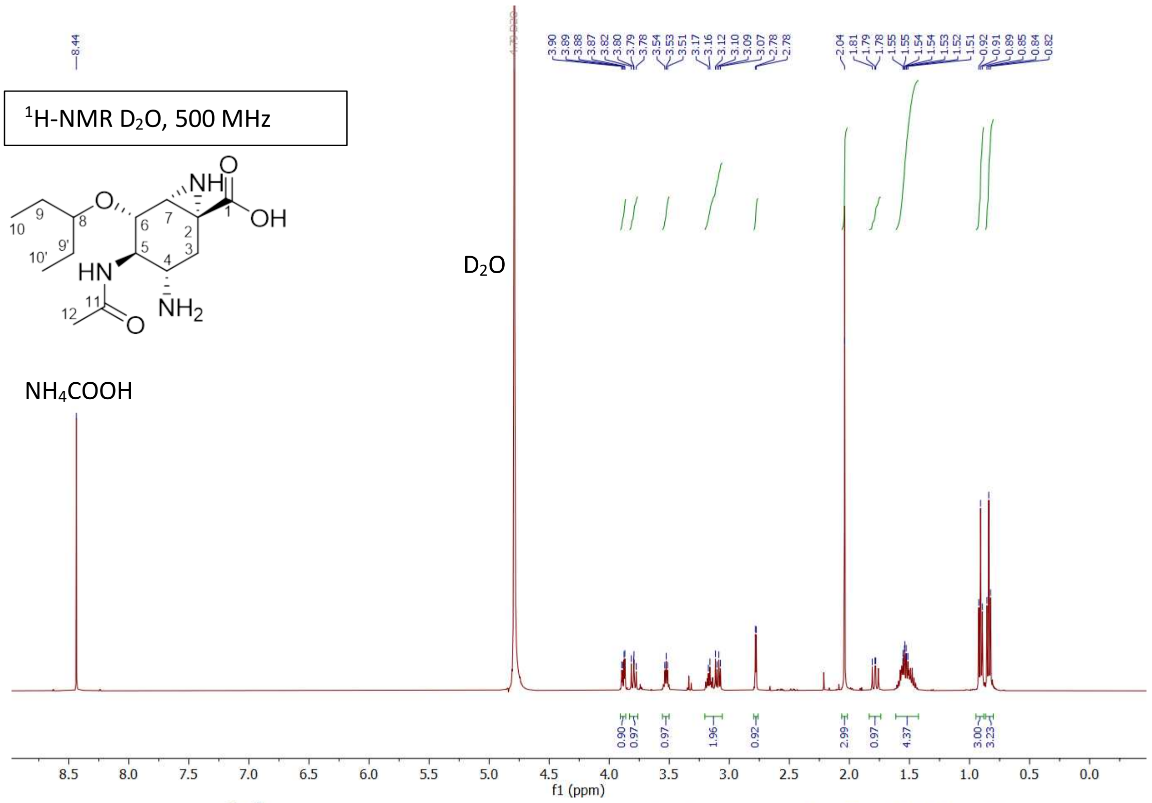

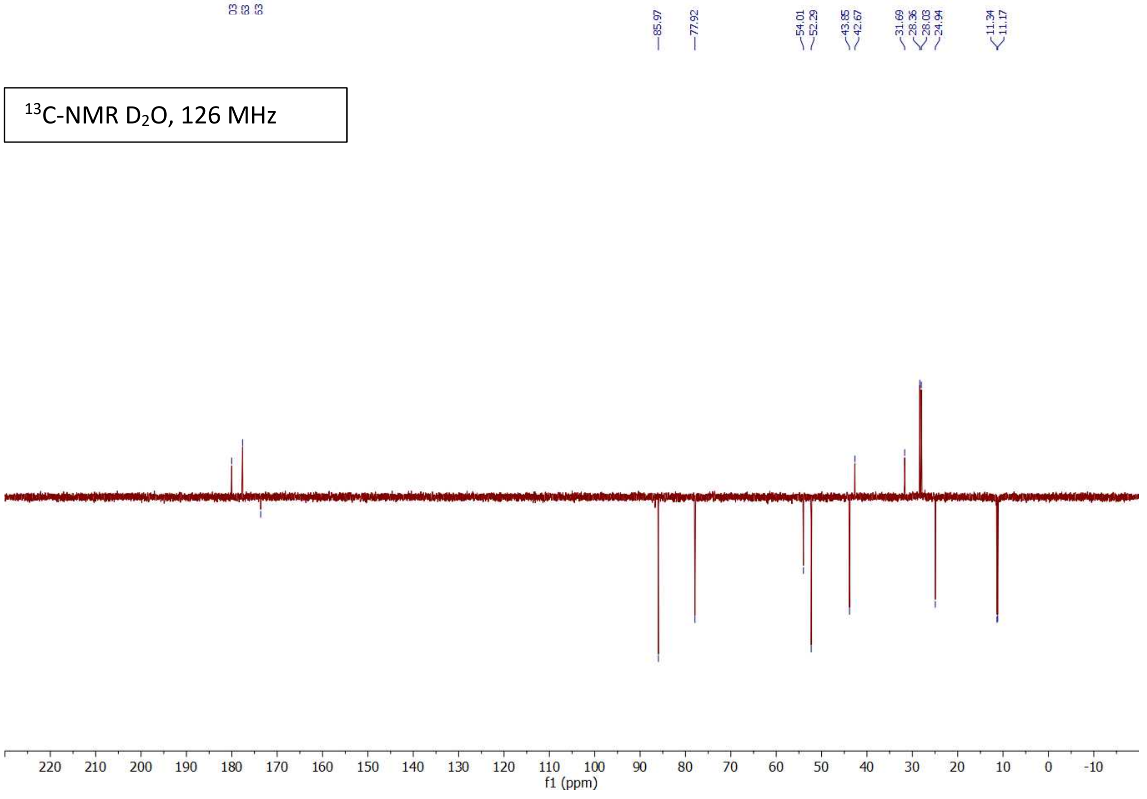

H-H COSY-NMR D_2O, 101

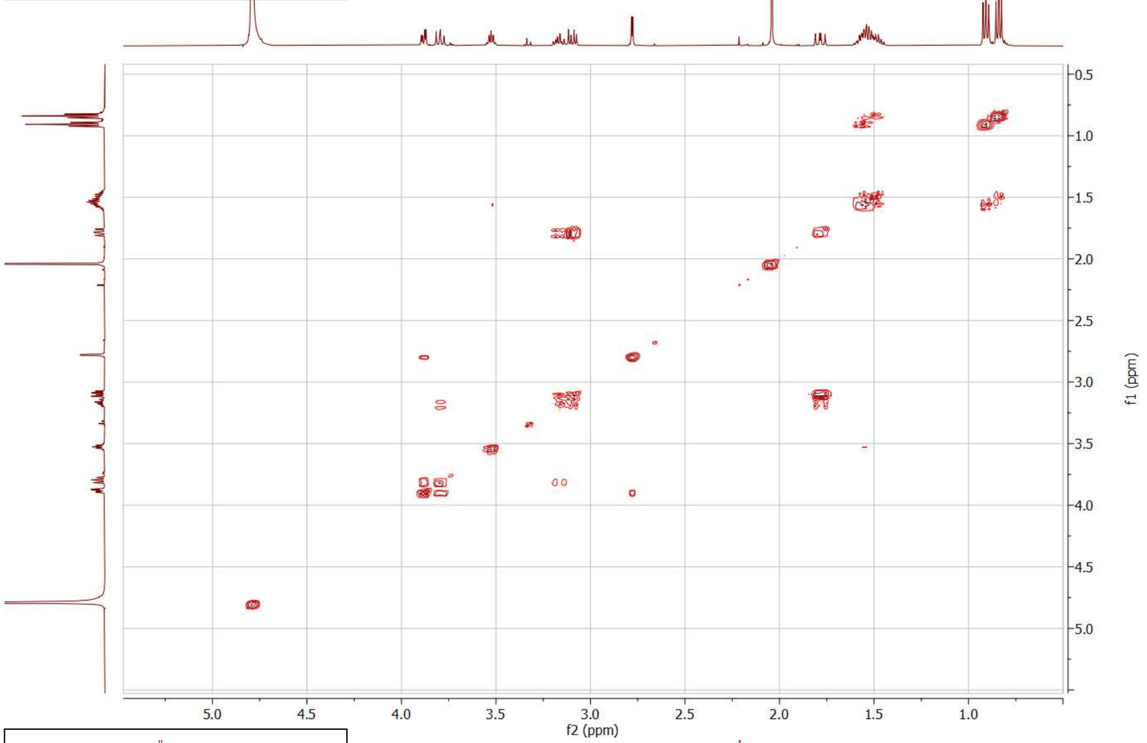

H-C HSQC-NMR D_2O, 101

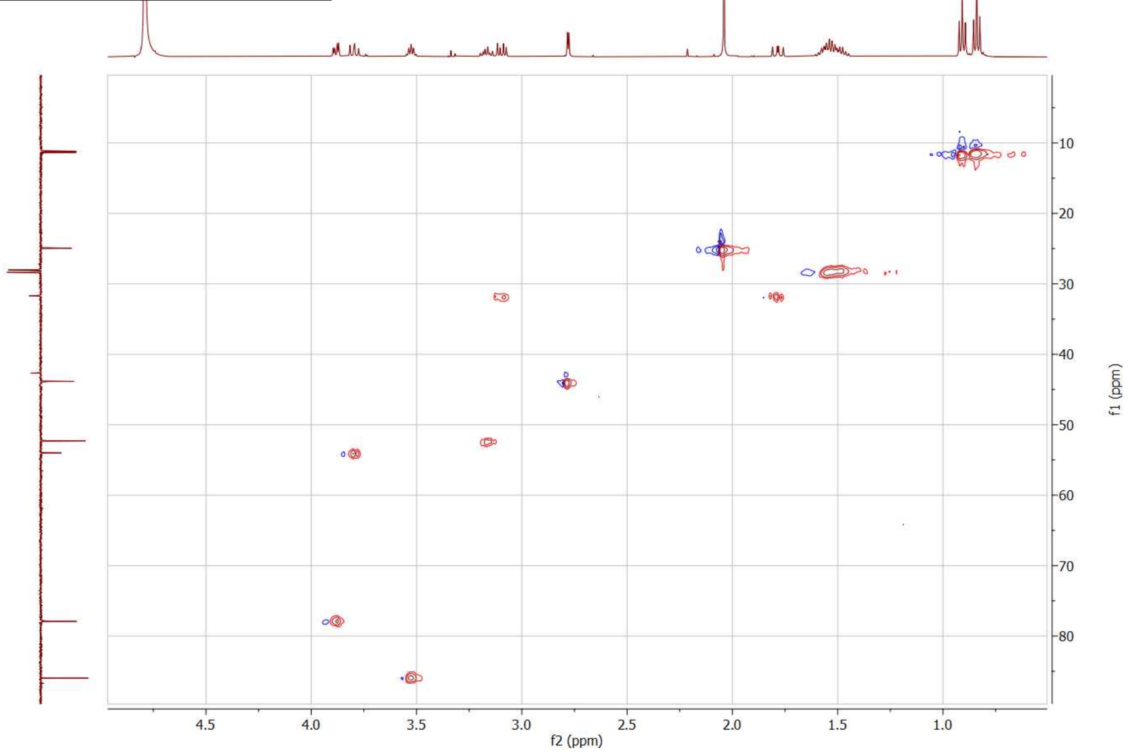

^{1}H-NMR $CDCl_3$, 500 MHz

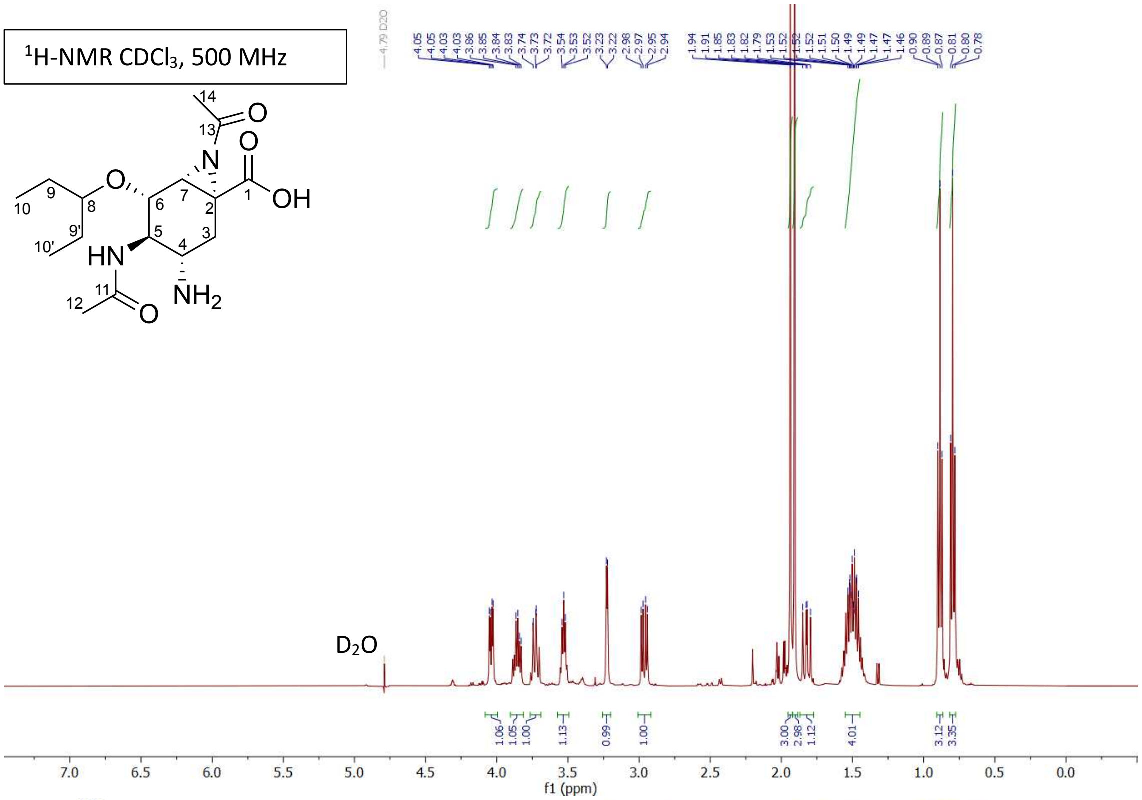

^{13}C-NMR $CDCl_3$, 126 MHz

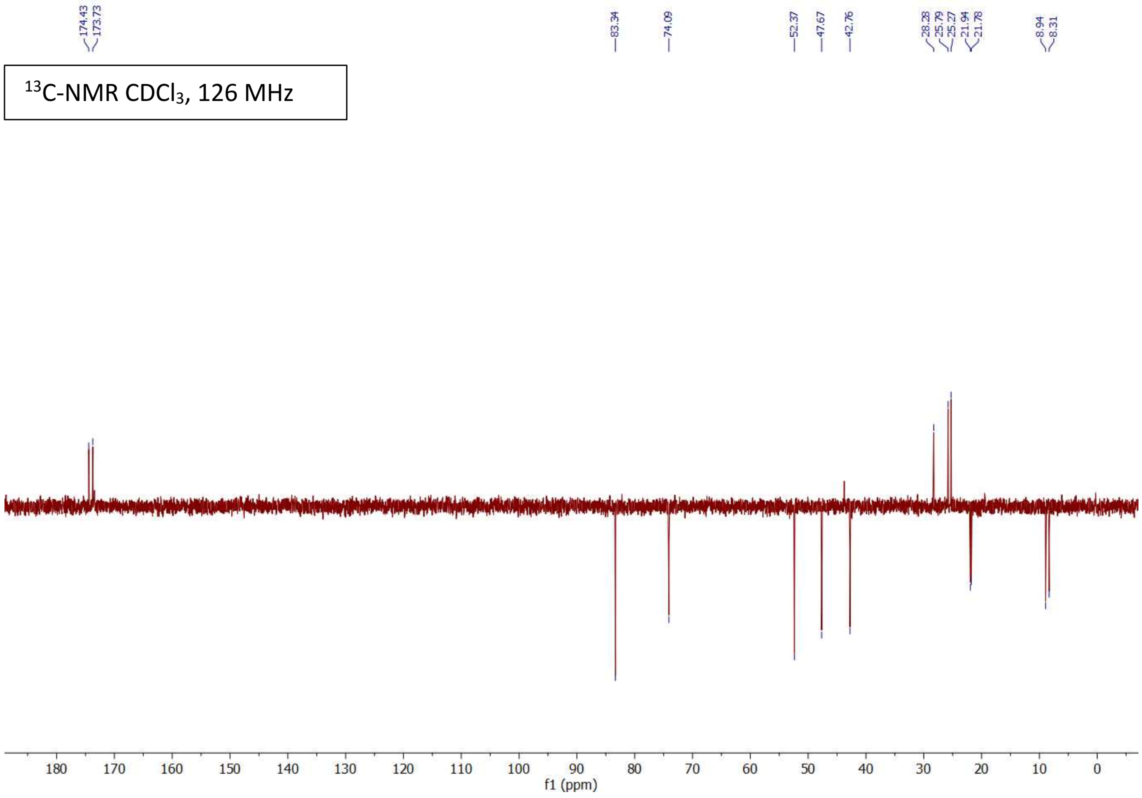

H-H COSY-NMR $CDCl_3$, 101

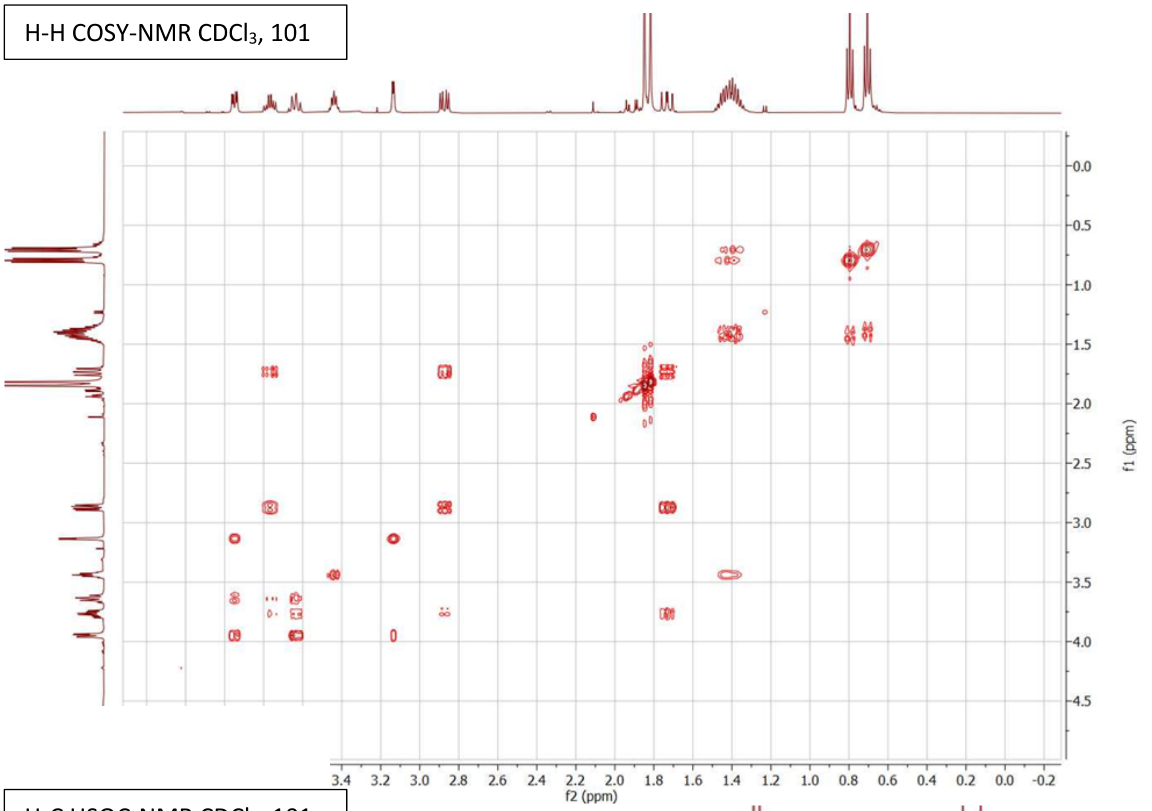

H-C HSQC-NMR $CDCl_3$, 101

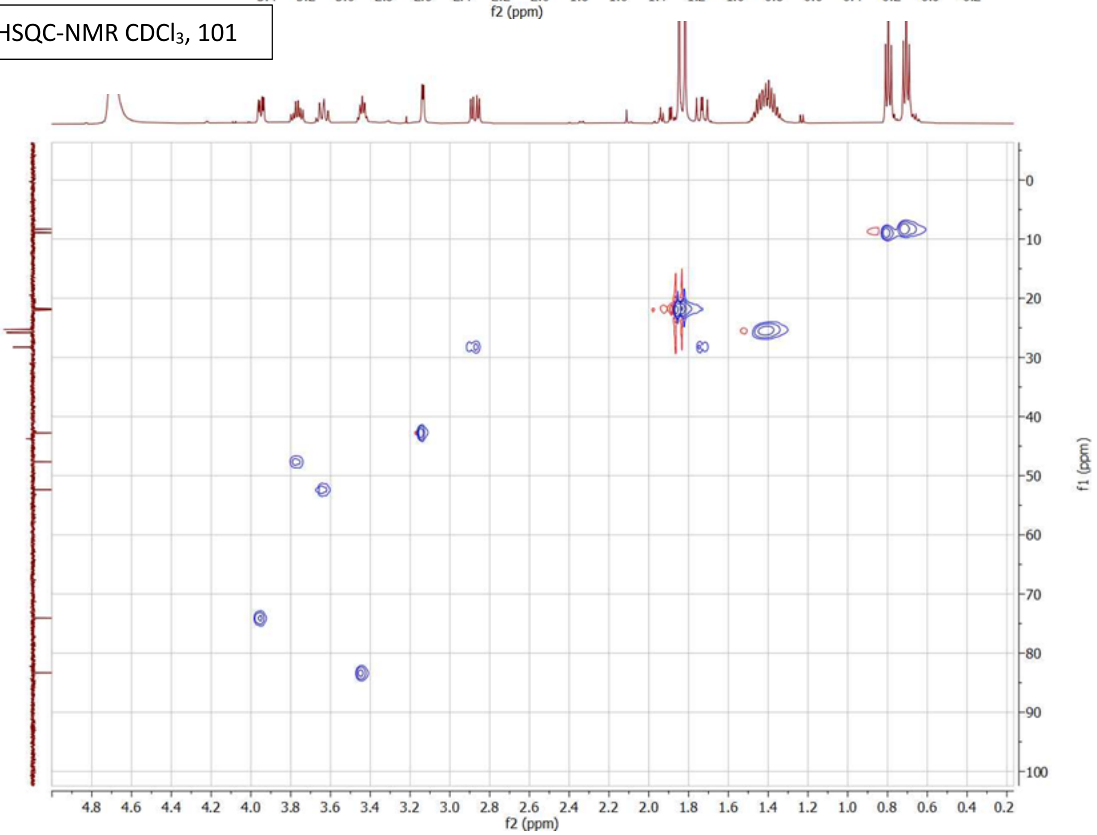

Compound **S4**

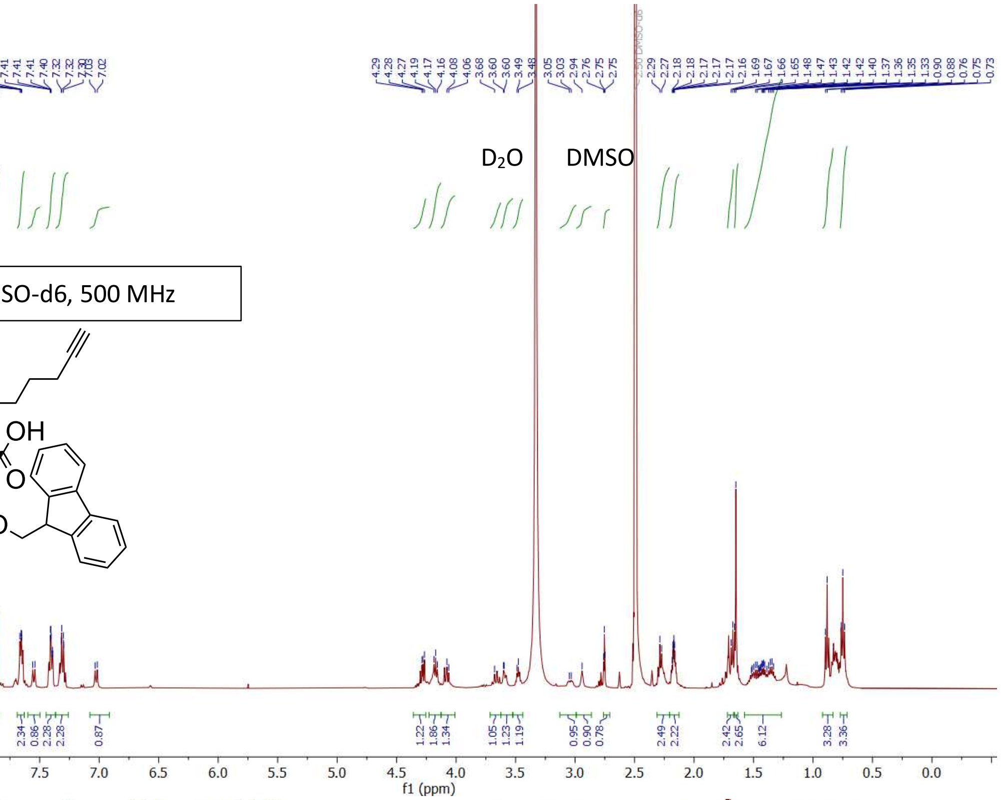

^{1}H-NMR DMSO-d6, 500 MHz

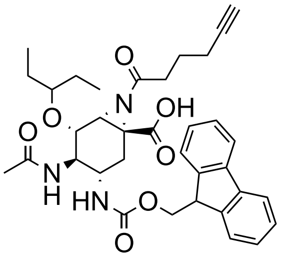

^{13}C-NMR DMSO-d6, 126 MHz

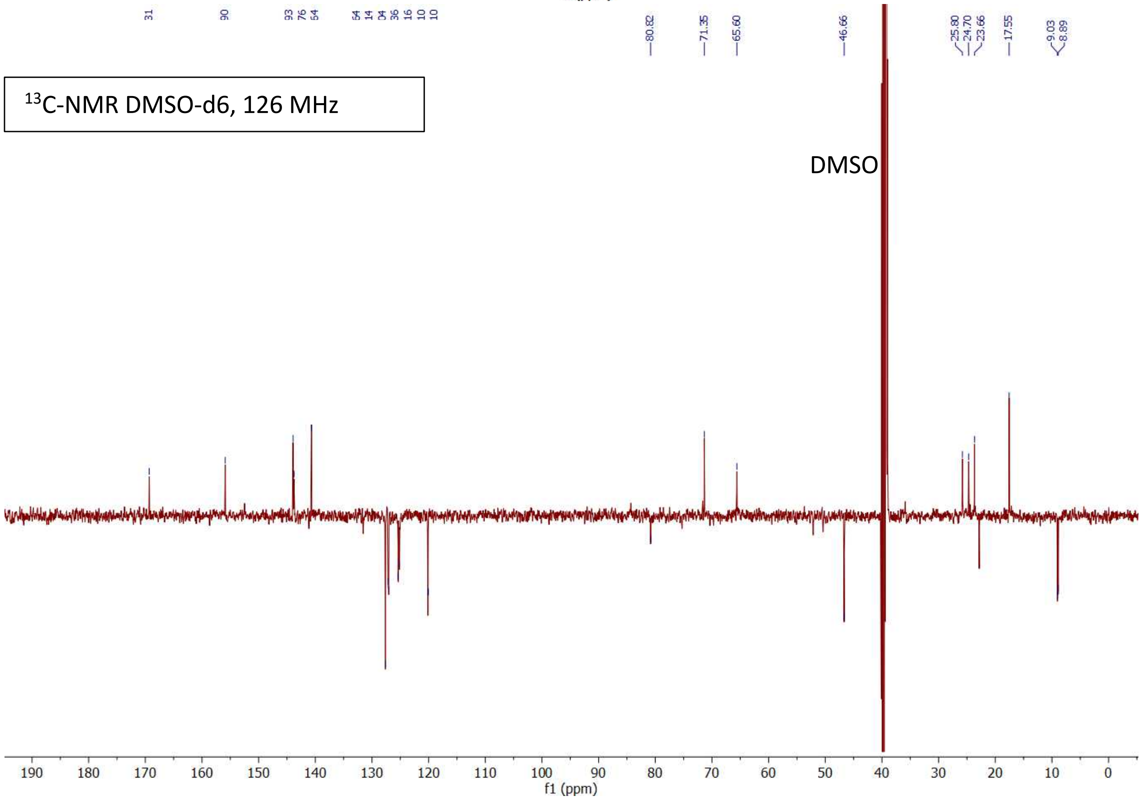

H-H COSY-NMR DMSO-d6, 101 MHz

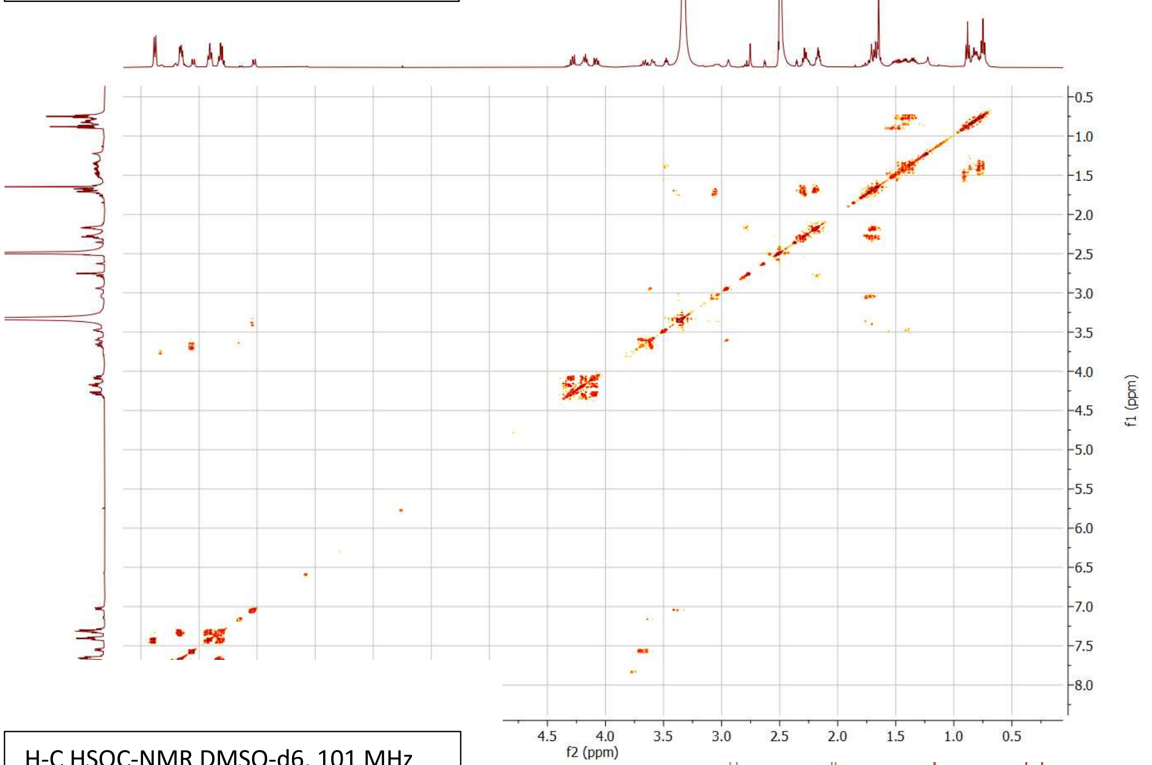

H-C HSQC-NMR DMSO-d6, 101 MHz

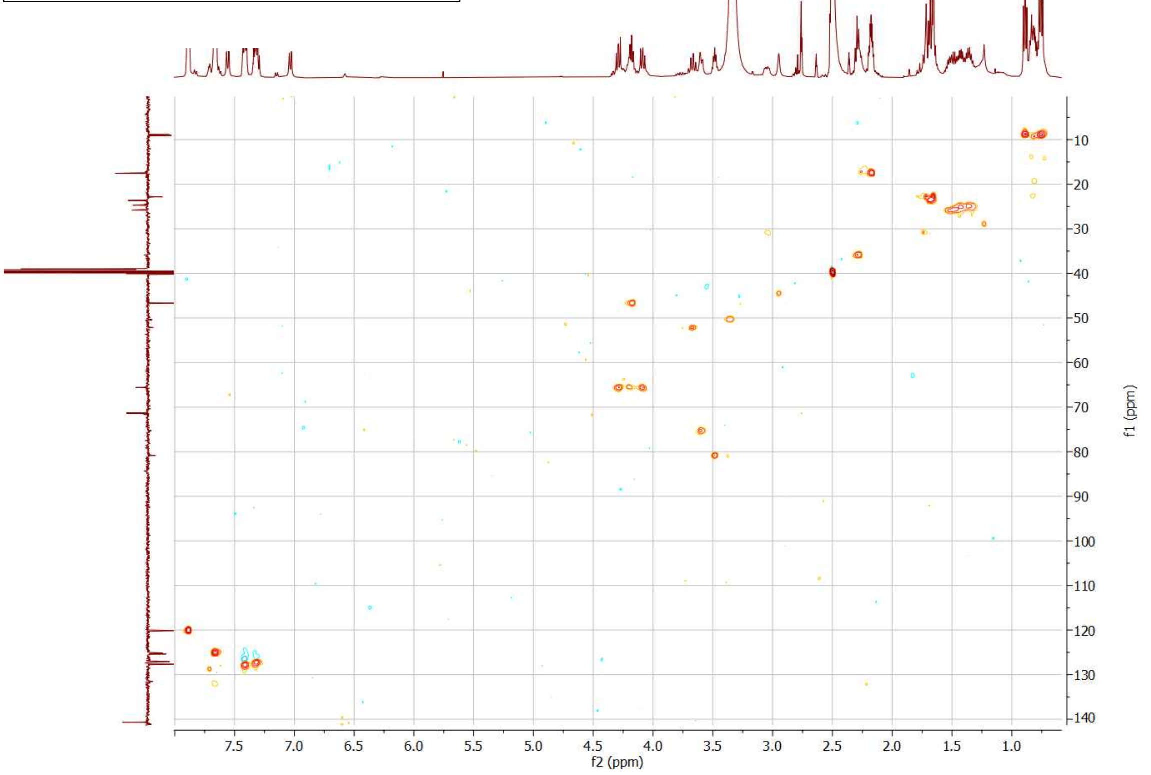

Compound **9**

^{1}H-NMR D_2O, 500 MHz

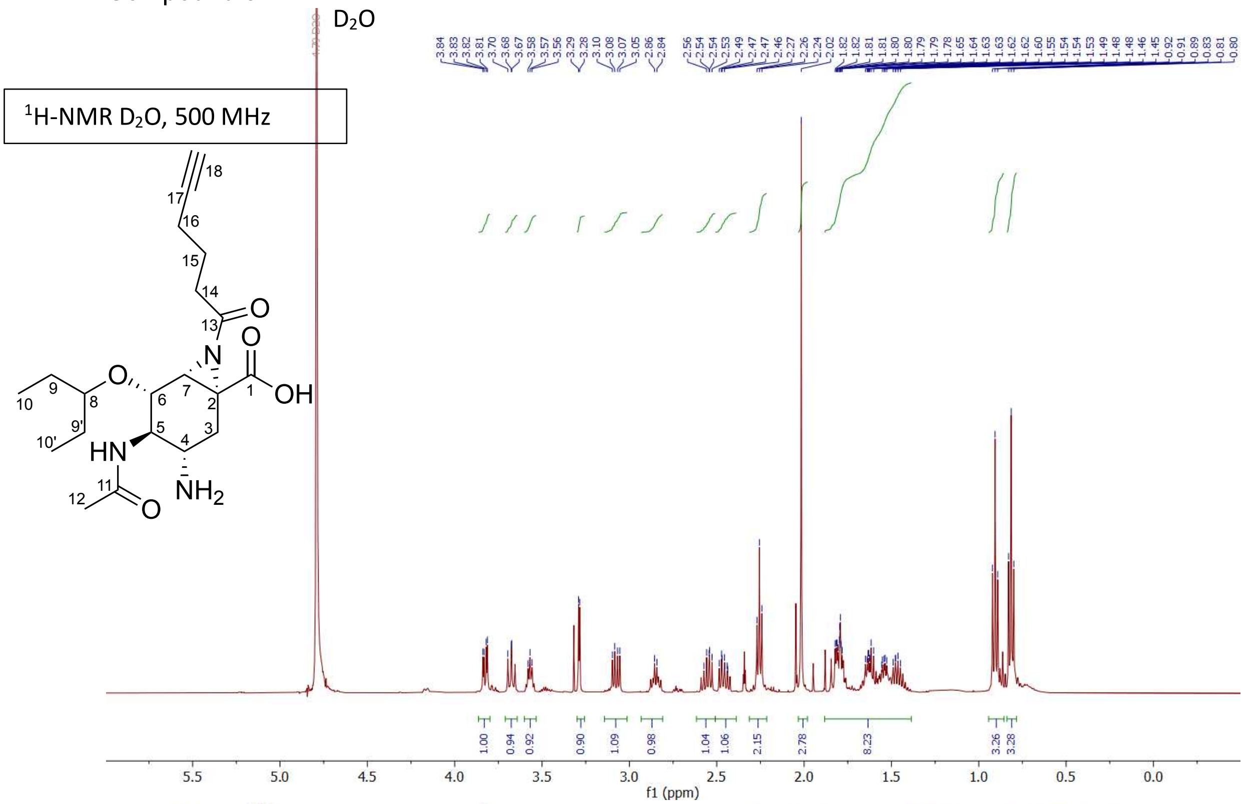

^{13}C-NMR D_2O, 126 MHz

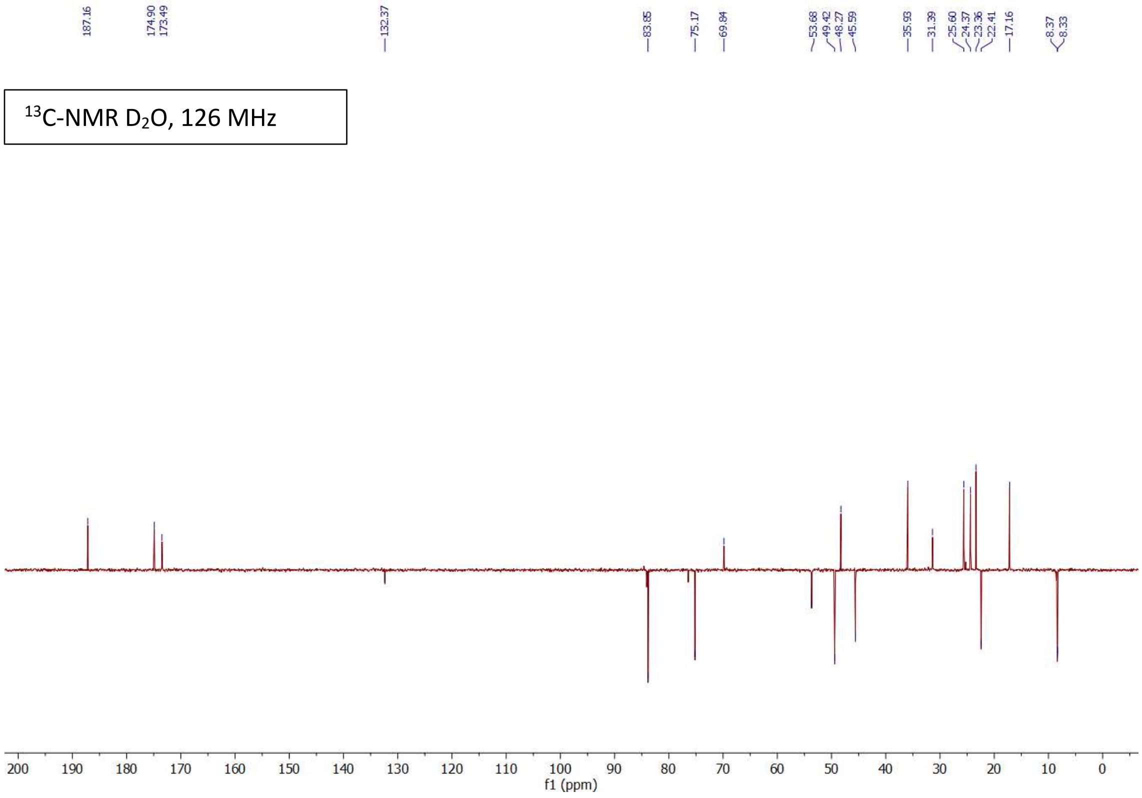

H-H COSY-NMR D_2O, 101 MHz

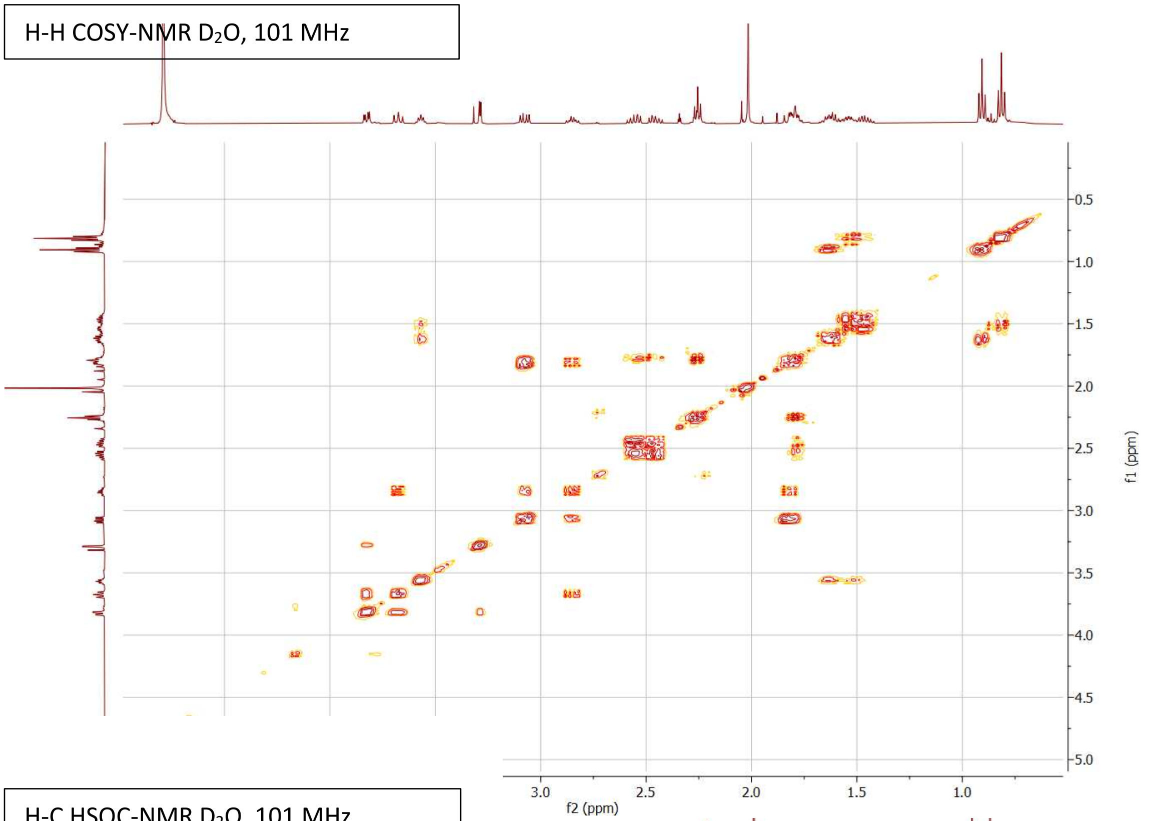

H-C HSQC-NMR D_2O, 101 MHz

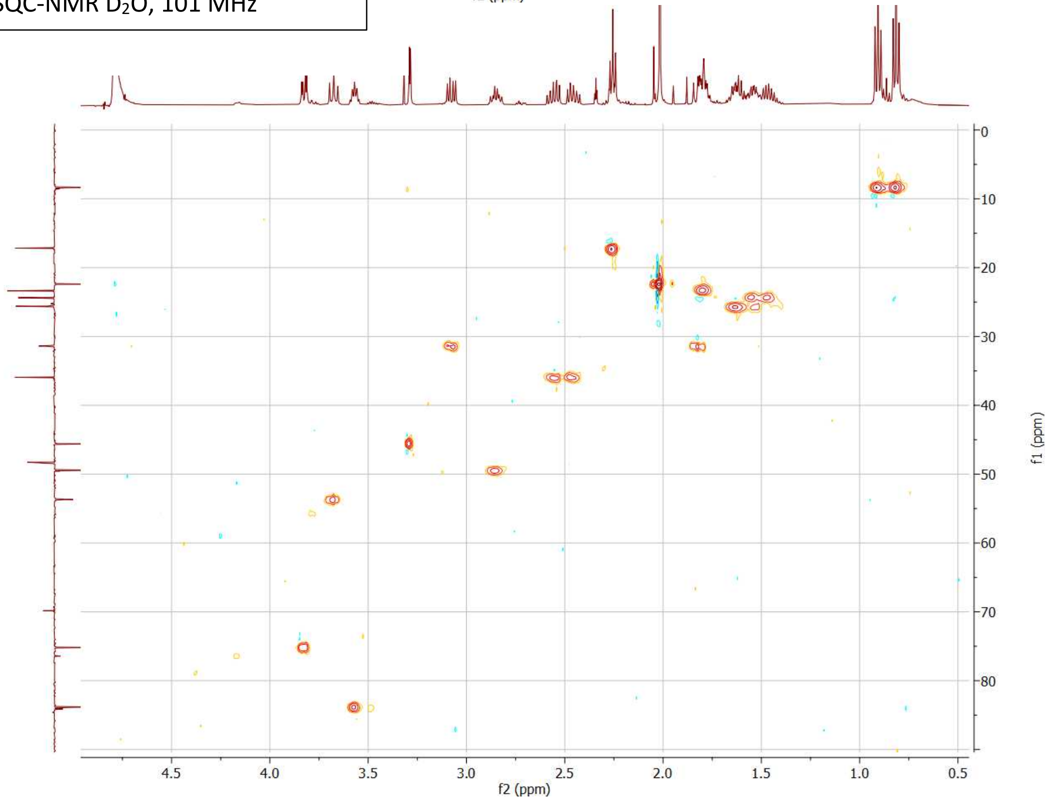

Compound **10**

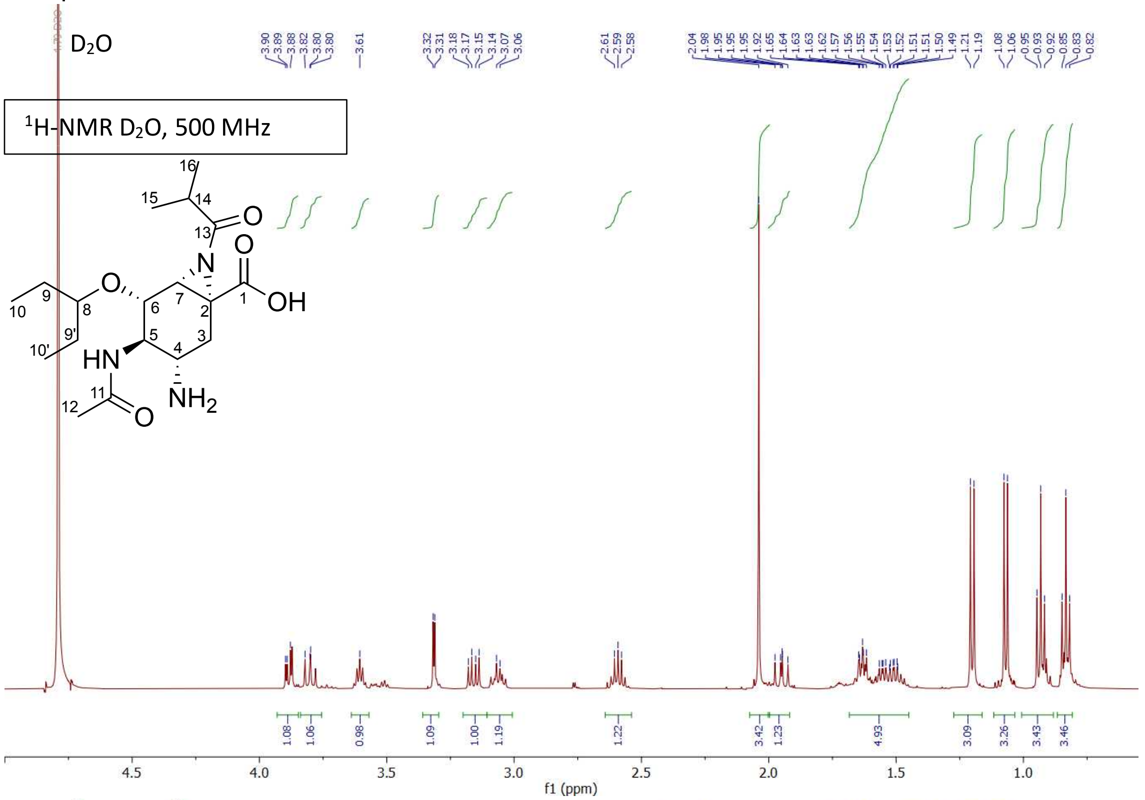

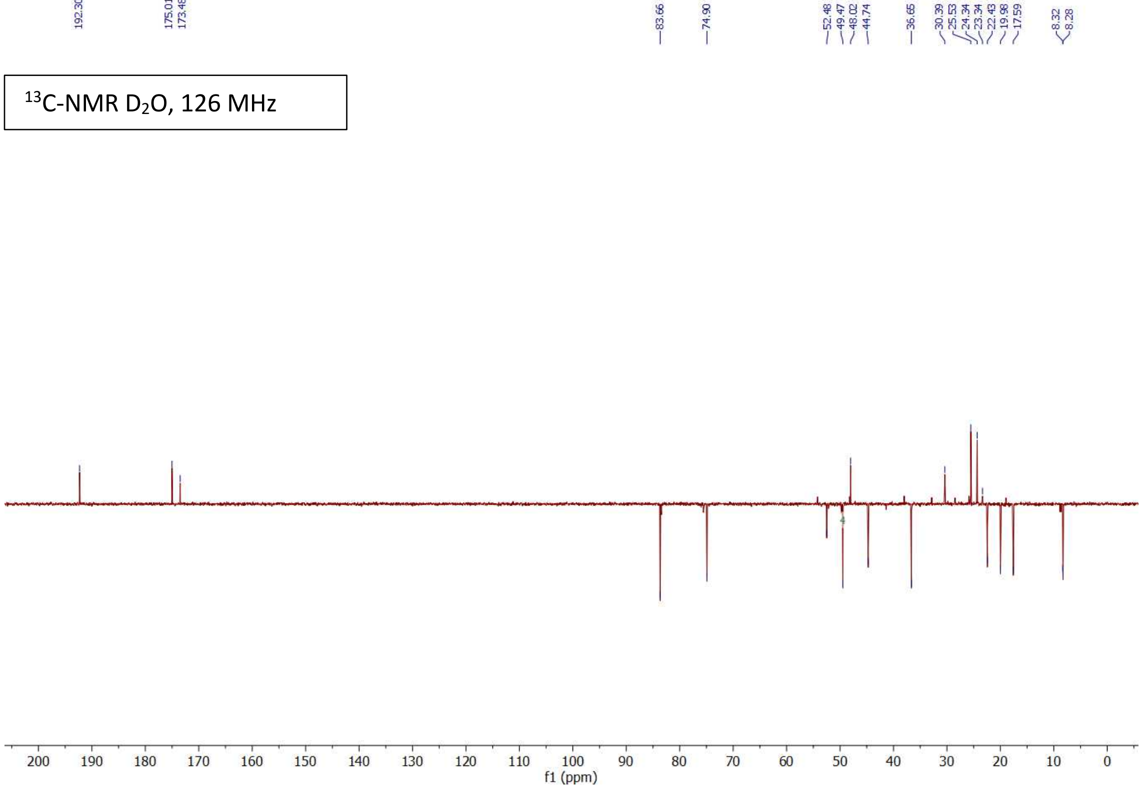

H-H COSY-NMR D_2O, 101 MHz

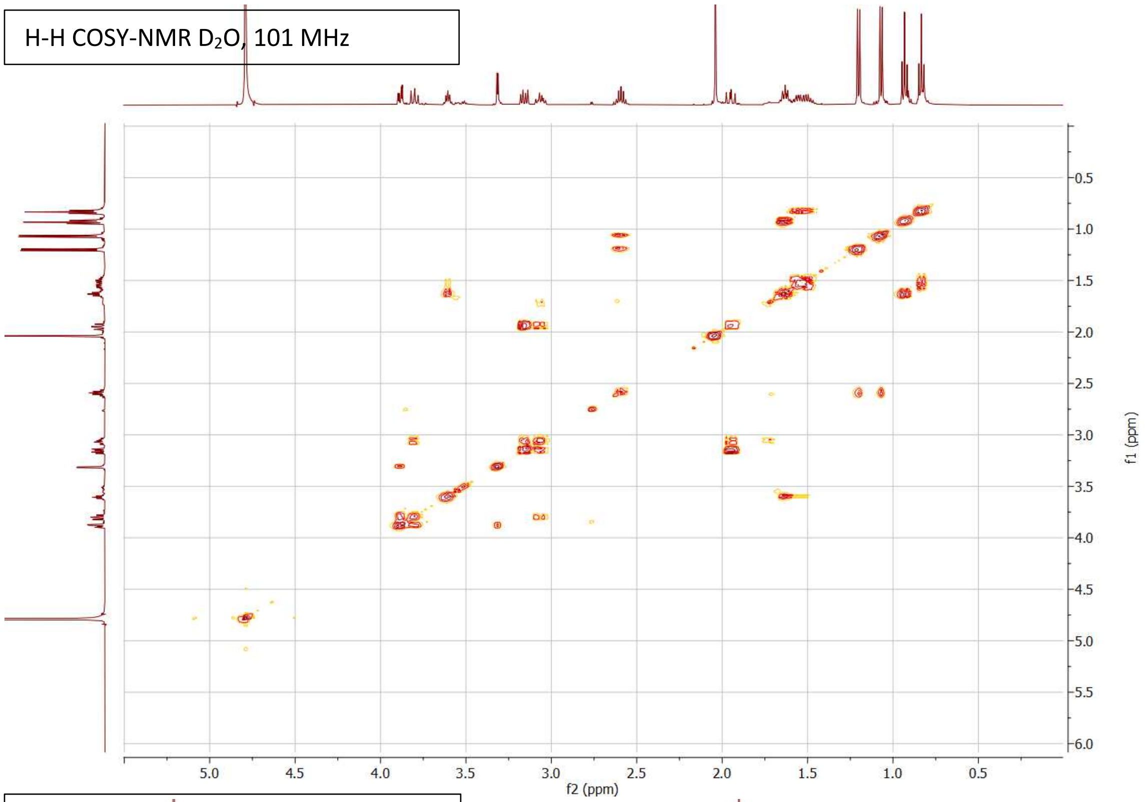

H-C HSQC-NMR D_2O, 101 MHz

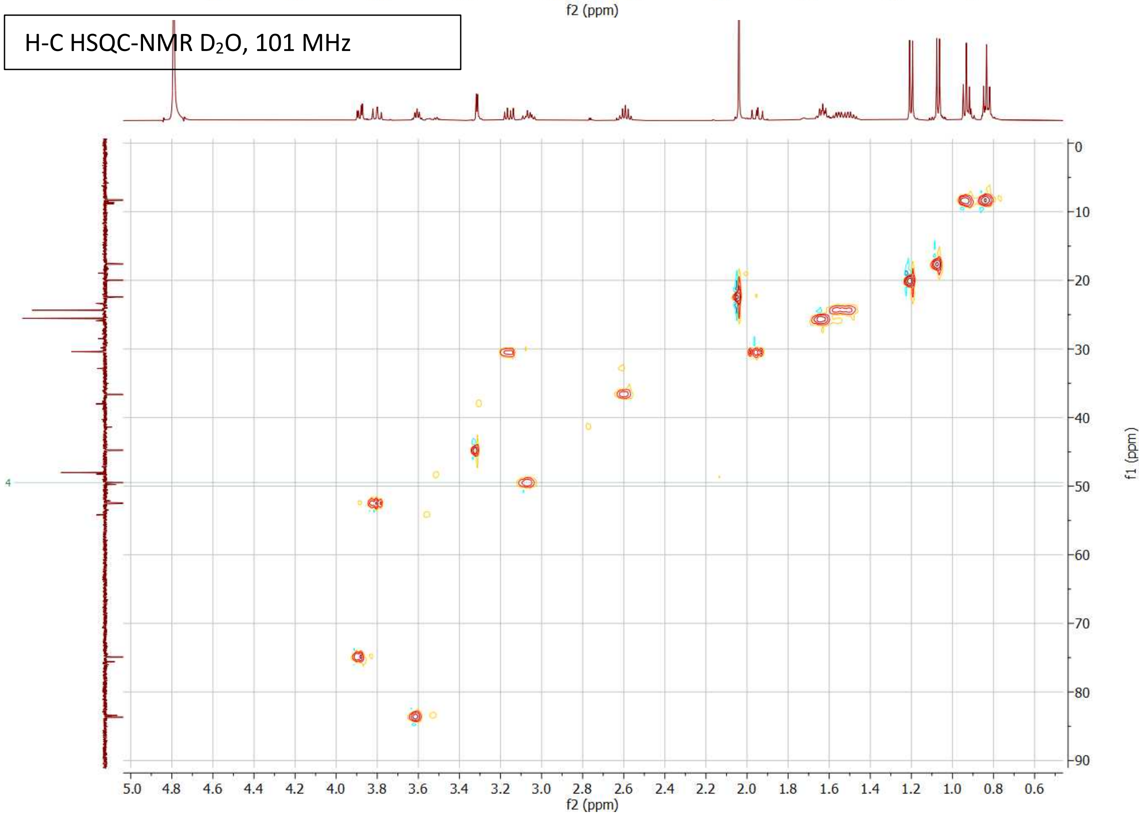

Compound **S5**

^{1}H-NMR $CDCl_3$, 400 MHz

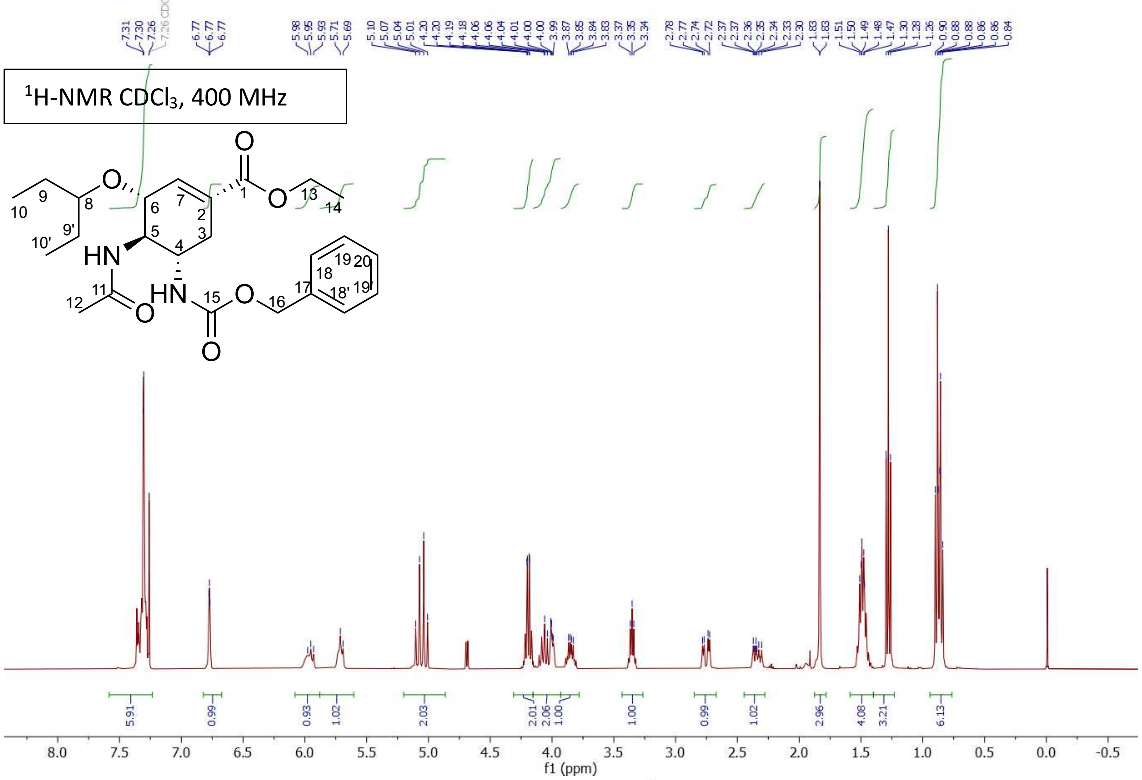

^{13}C-NMR $CDCl_3$, 101 MHz

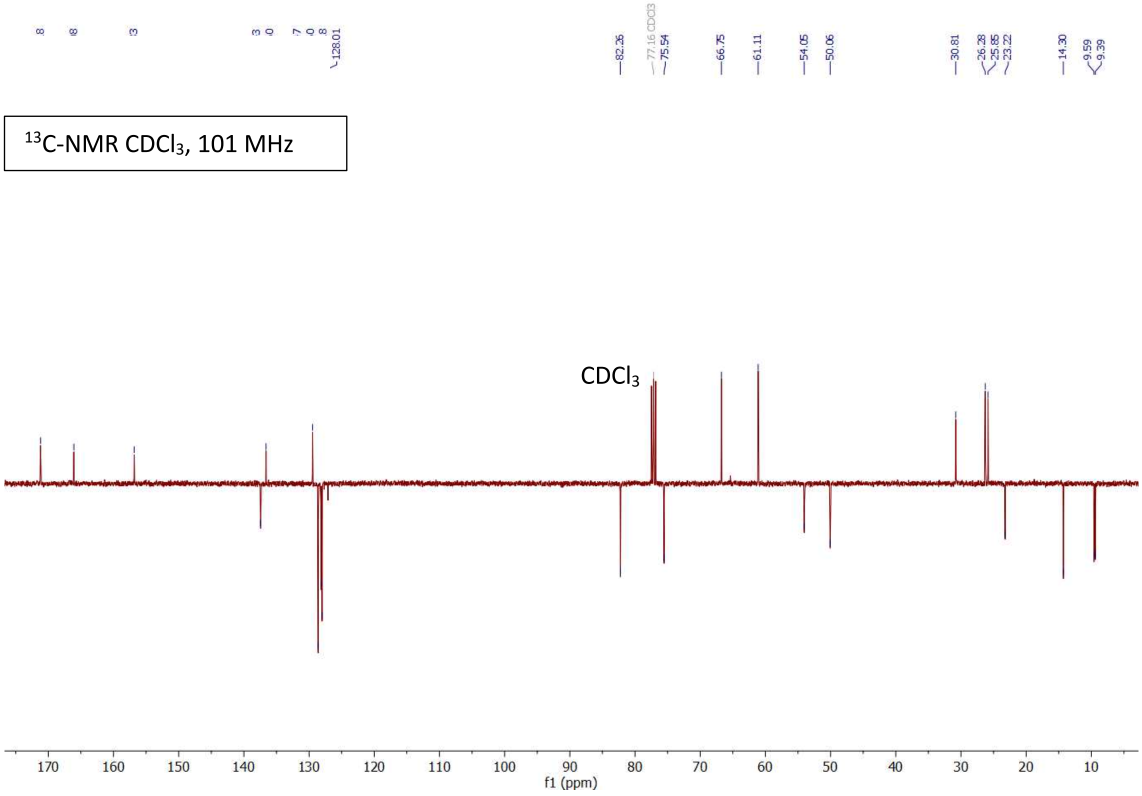

H-H COSY-NMR $CDCl_3$, 101 MHz

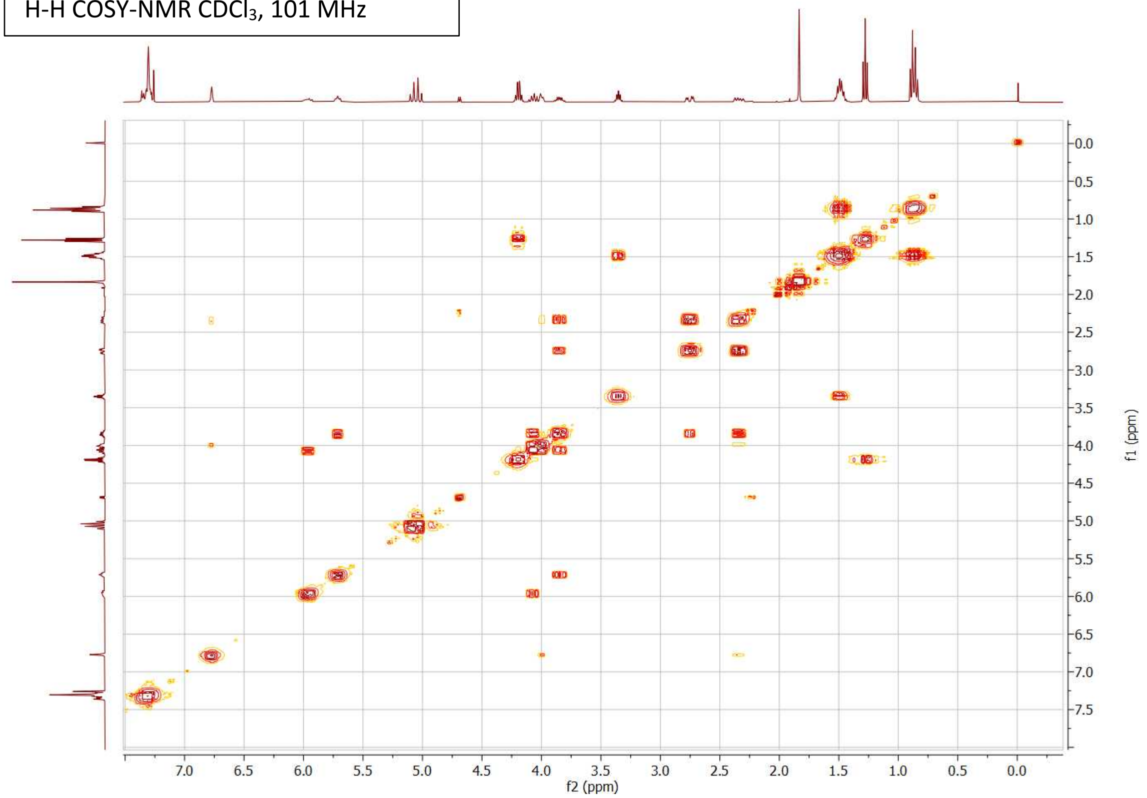

^{1}H-NMR $CDCl_3$, 500 MHz

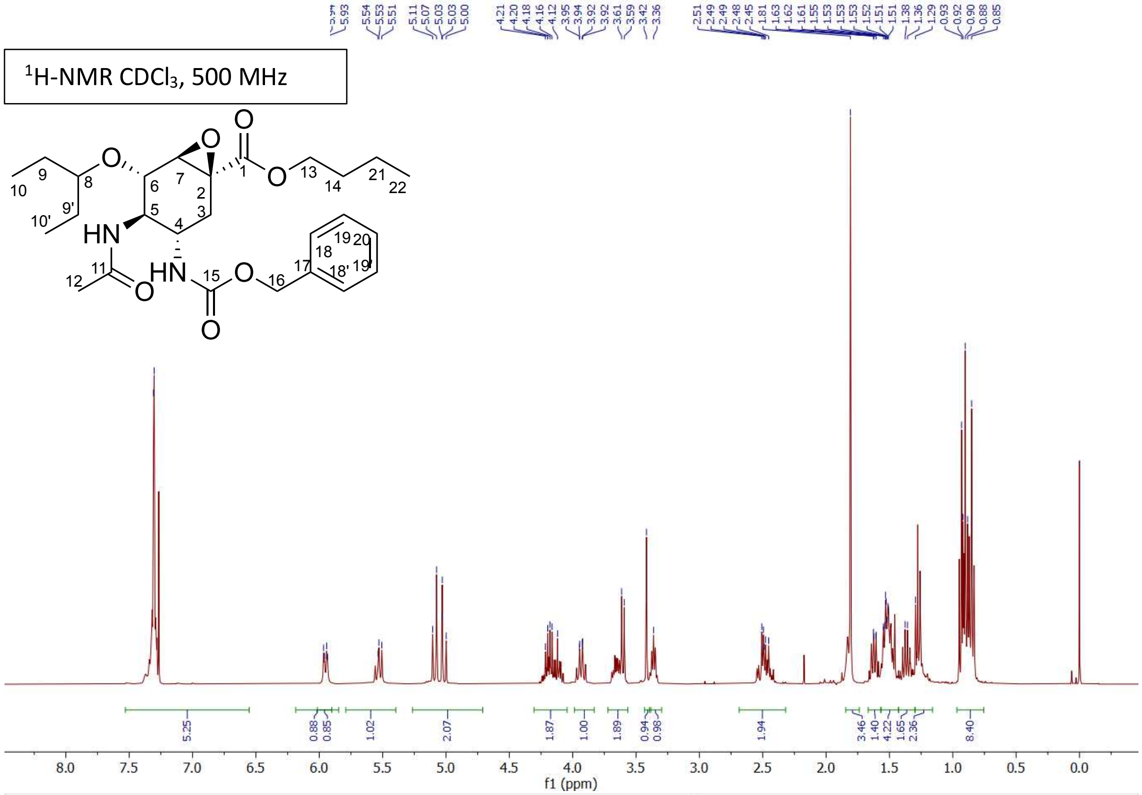

^{13}C-NMR $CDCl_3$, 126 MHz

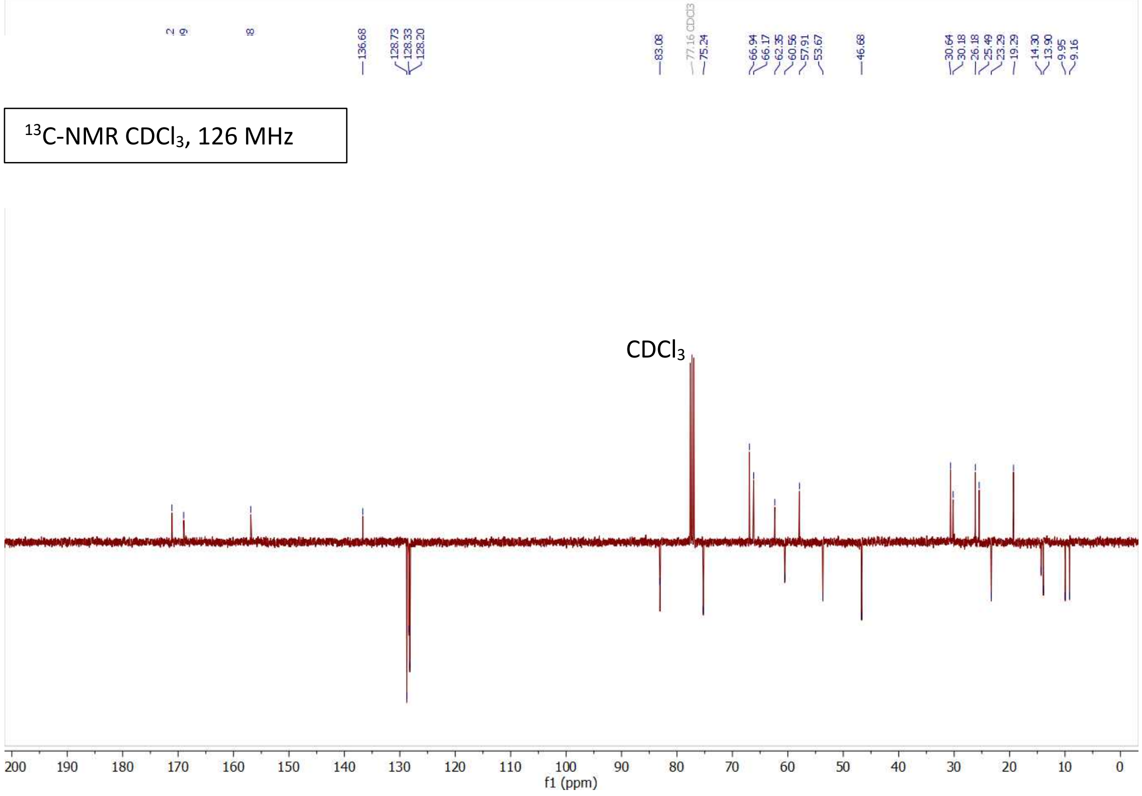

H-H COSY-NMR $CDCl_3$, 101 MHz

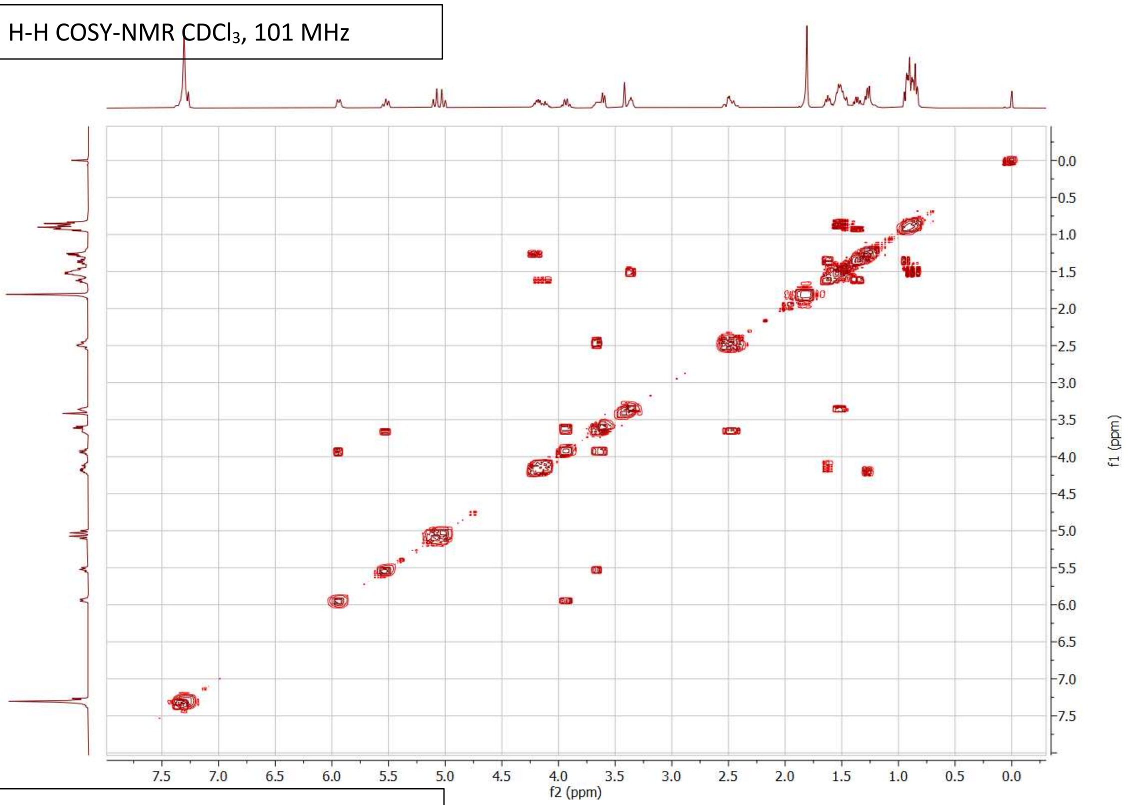

H-C HSQC-NMR $CDCl_3$, 101 MHz

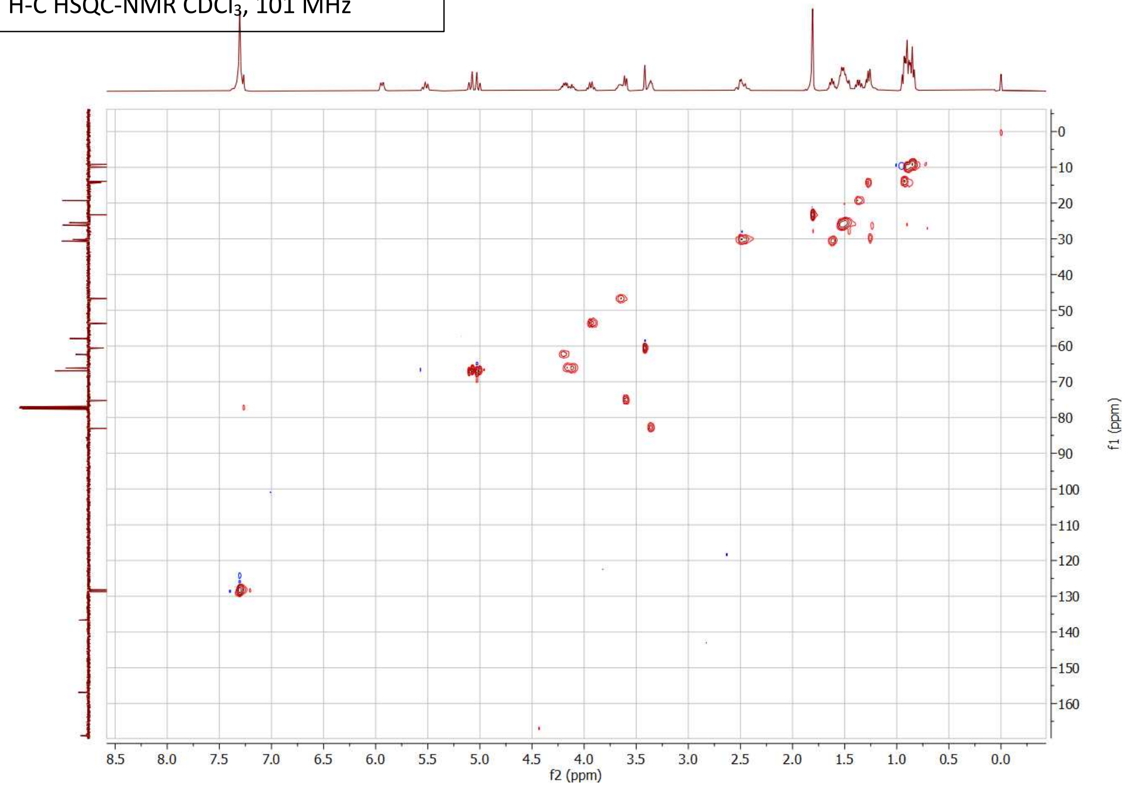

Compound **S7**

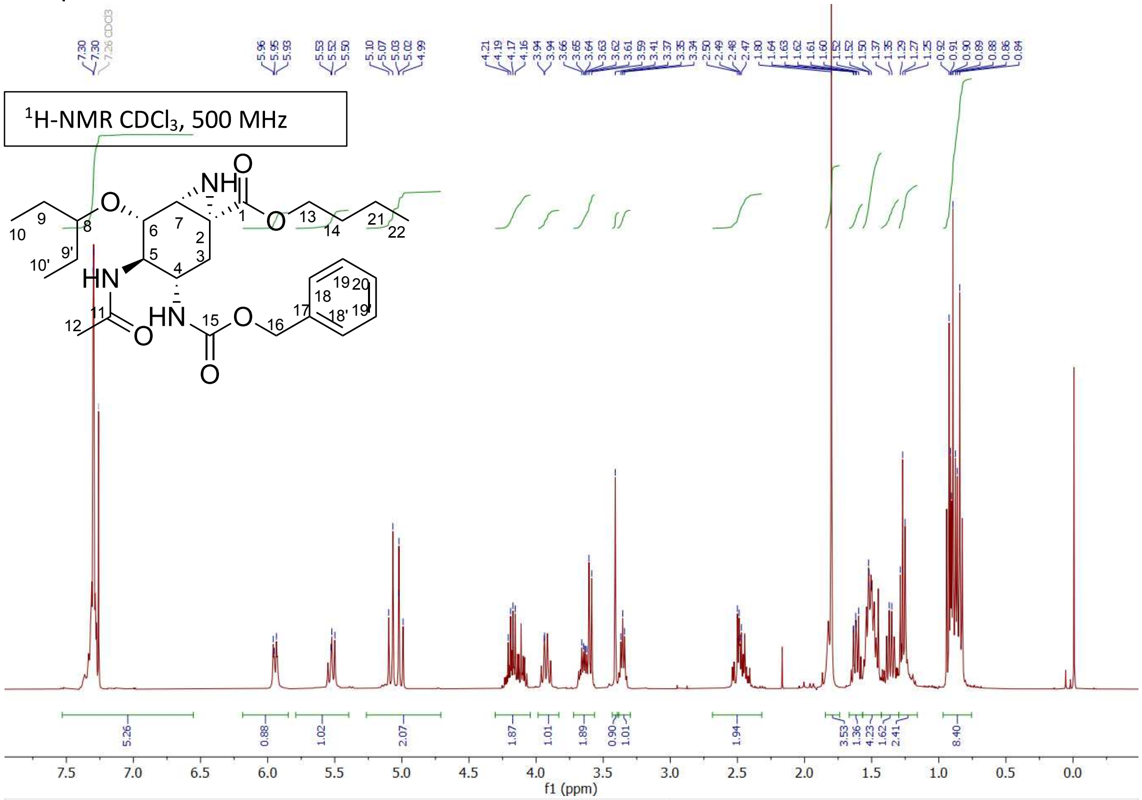

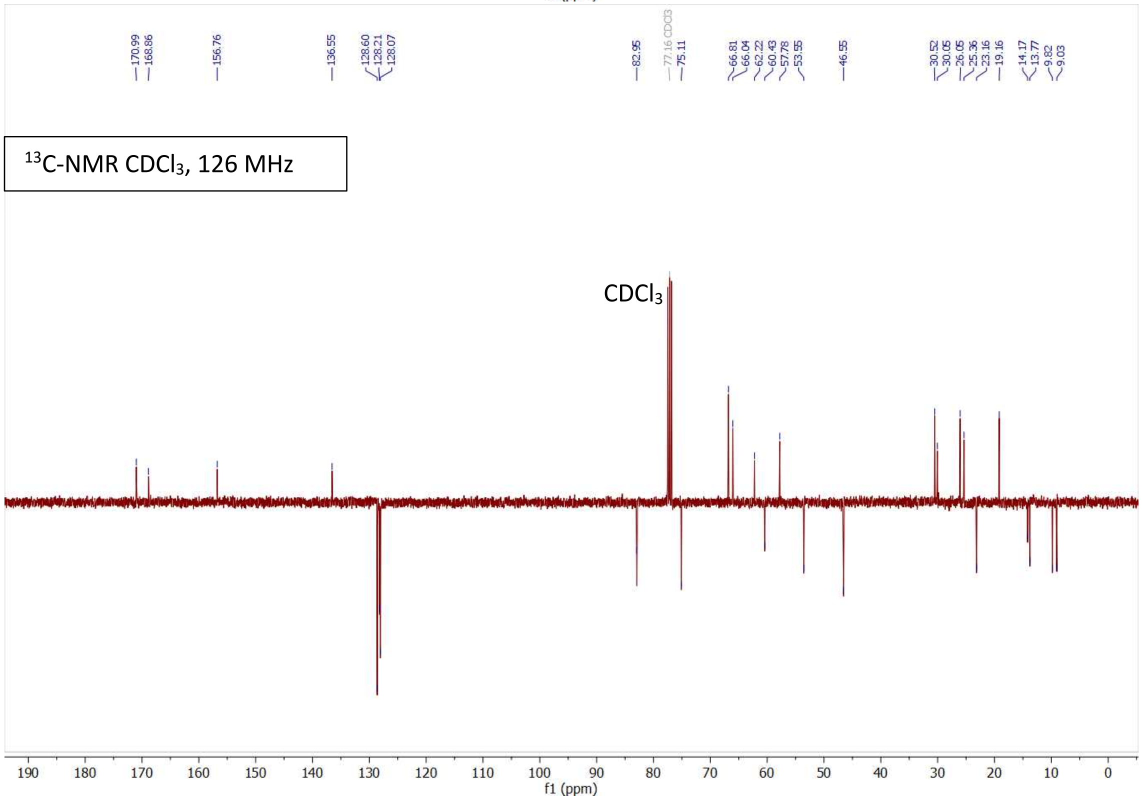

H-H COSY-NMR $CDCl_3$, 101 MHz

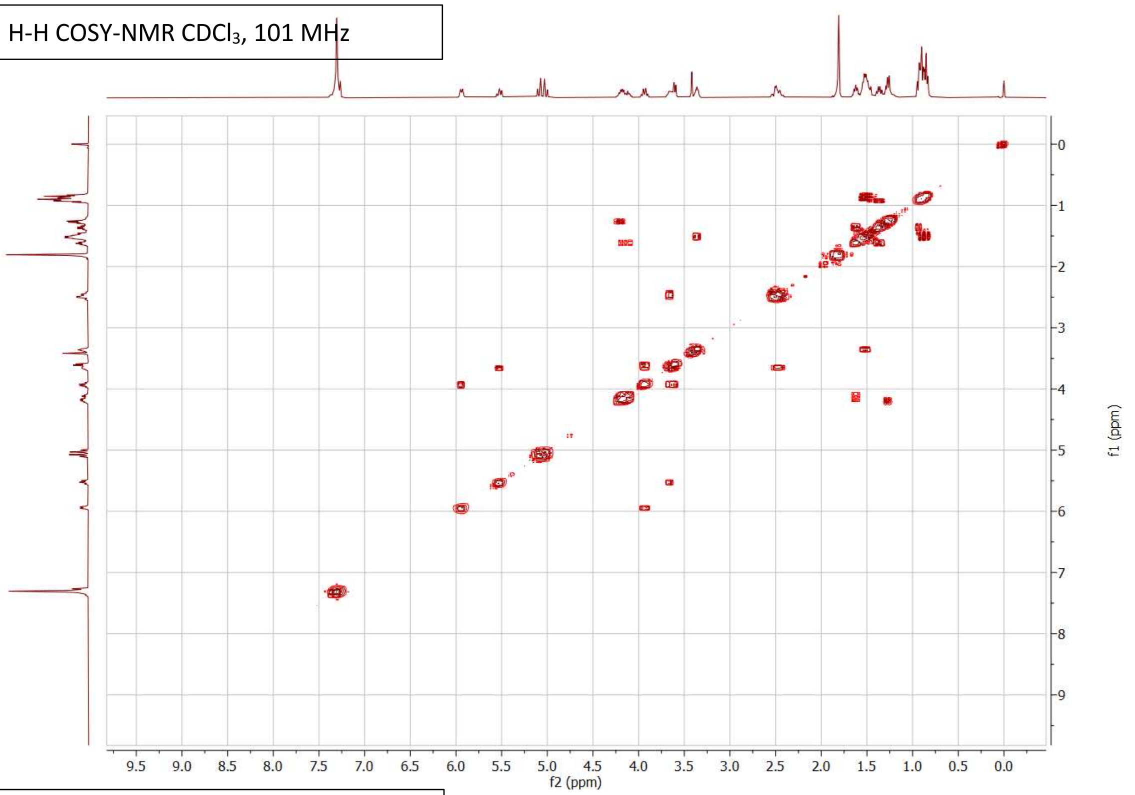

H-C HSQC-NMR $CDCl_3$, 101 MHz

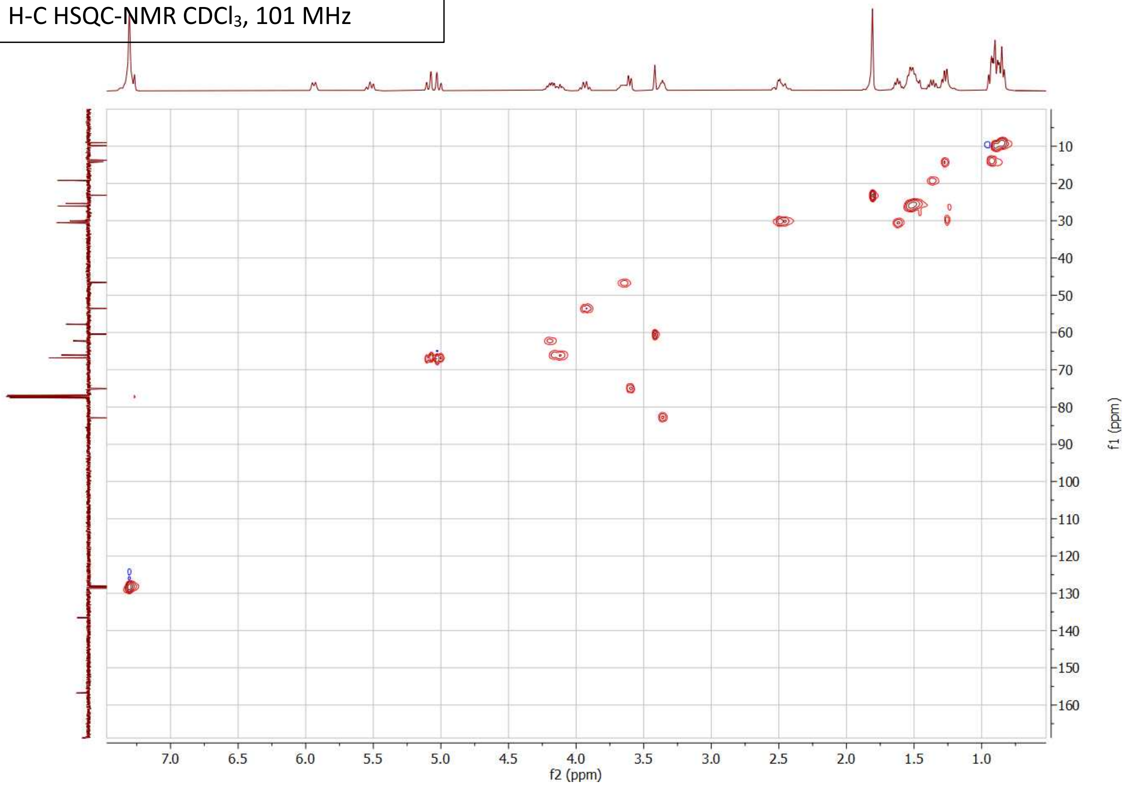

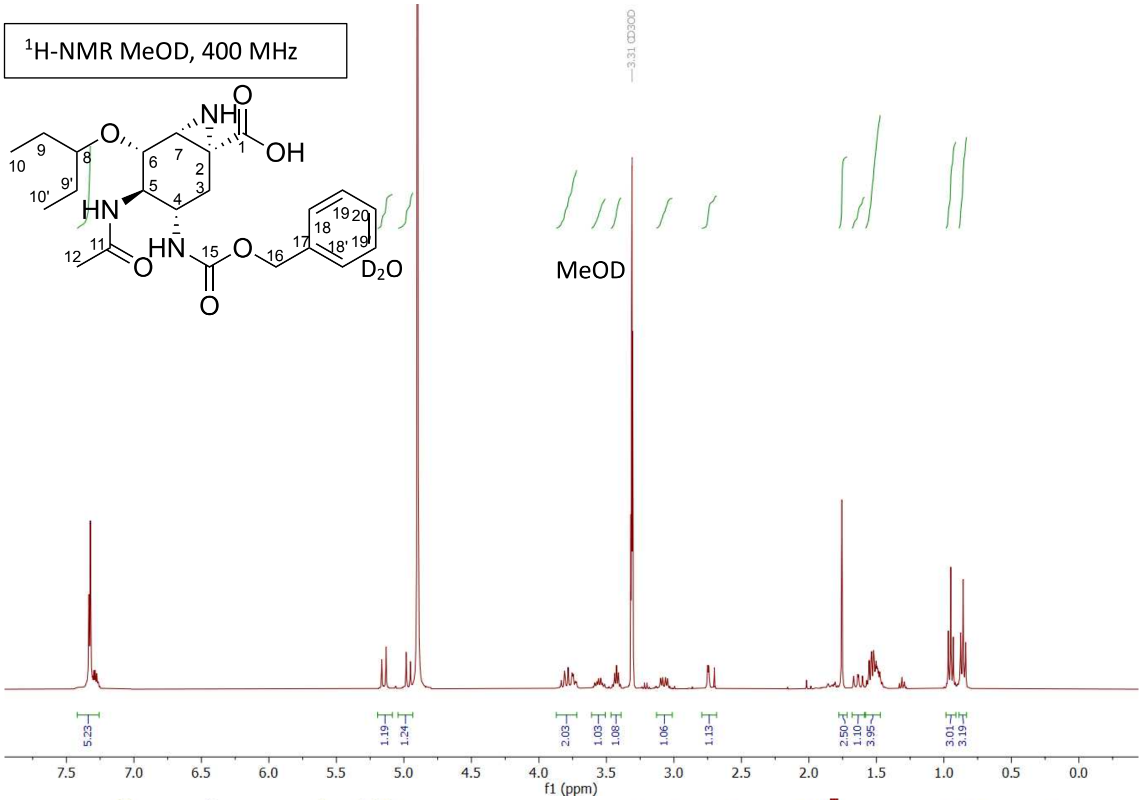
1H-NMR MeOD, 400 MHz
D2O
MeOD
3.31 CD3OD
5.23
1.19
1.24
2.03
1.03
1.08
1.06
1.13
2.50
1.10
3.95
3.01
3.19
f1 (ppm)

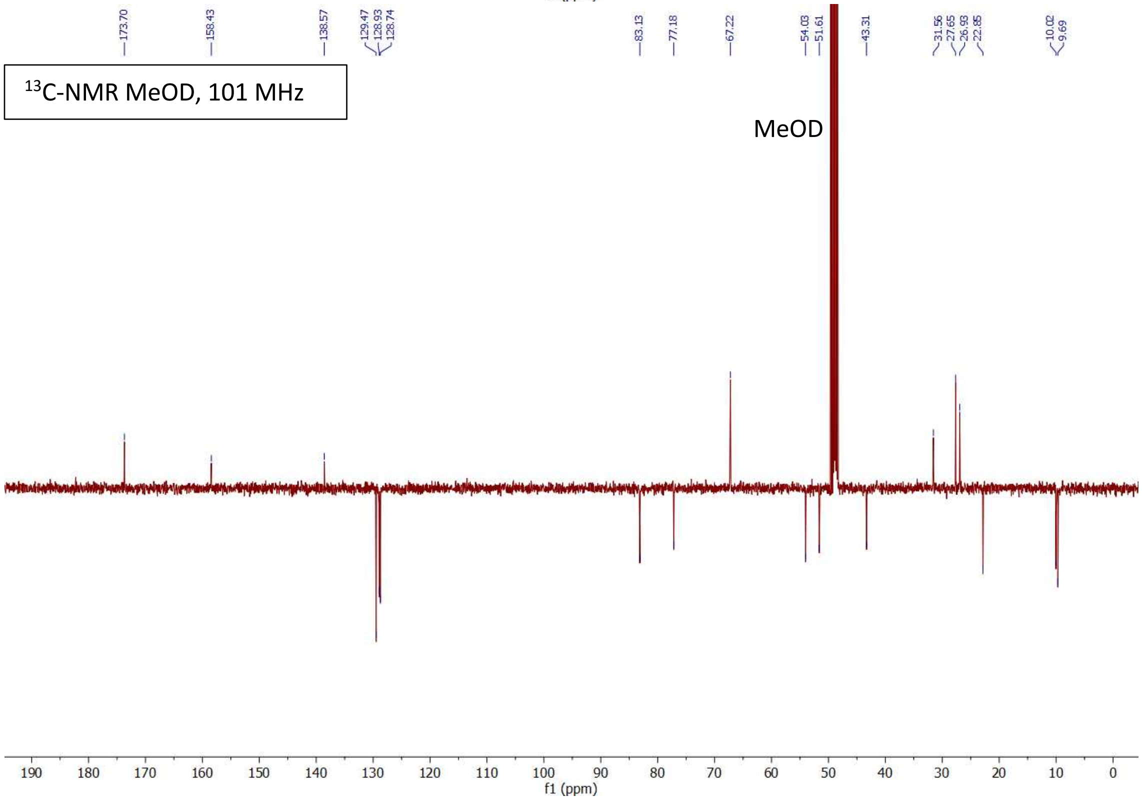
13C-NMR MeOD, 101 MHz
MeOD
173.70
158.43
138.57
129.47
128.93
128.74
83.13
77.18
67.22
54.03
51.61
43.31
31.56
27.65
26.93
22.85
10.02
9.69
f1 (ppm)

H-H COSY-NMR MeOD, 101 MHz

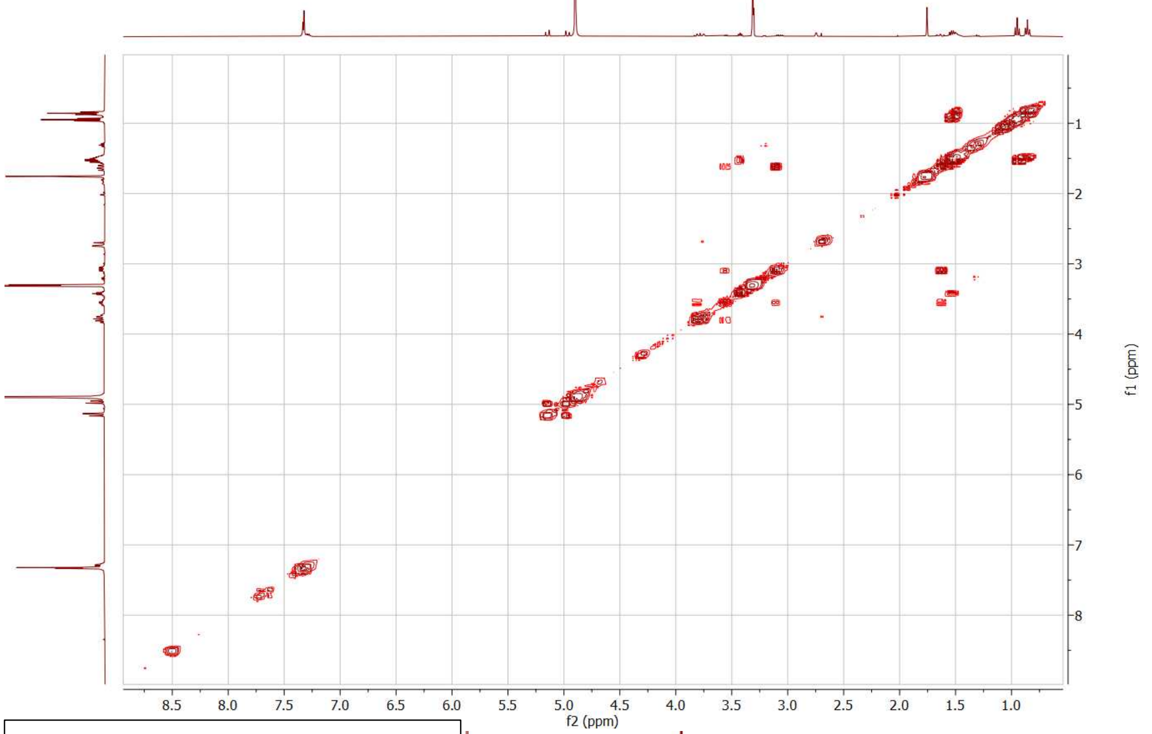

H-C HSQC-NMR MeOD, 101 MHz

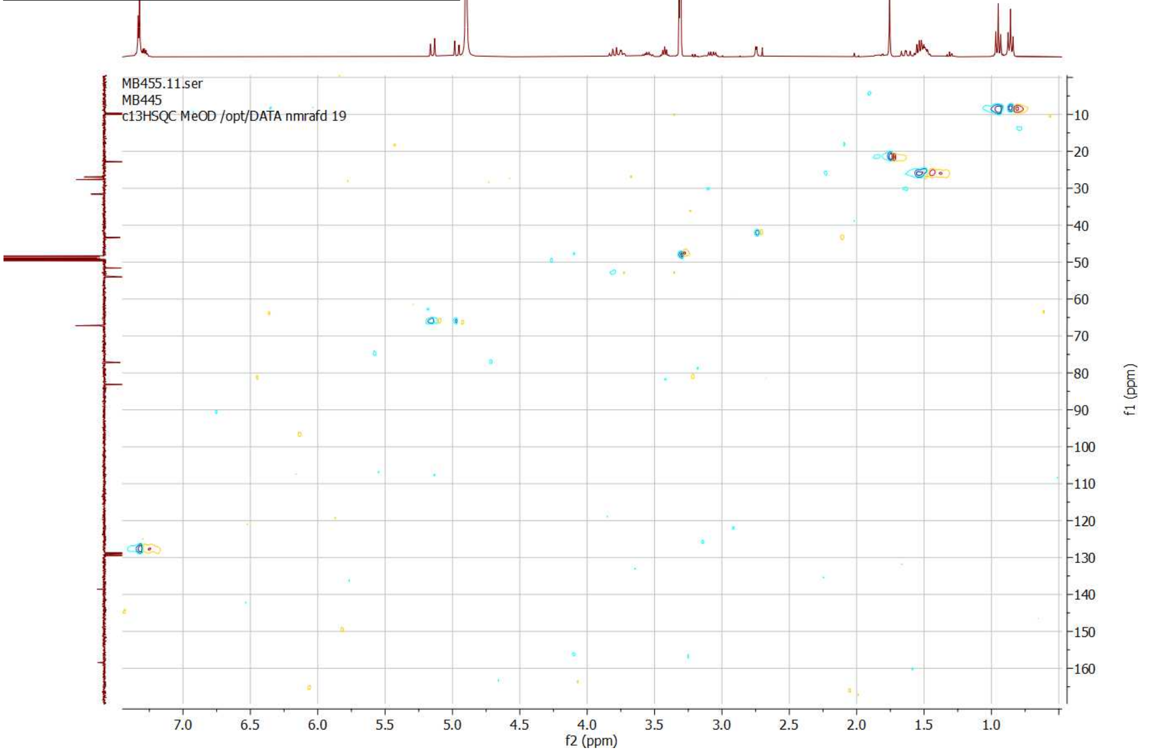

Compound **13**

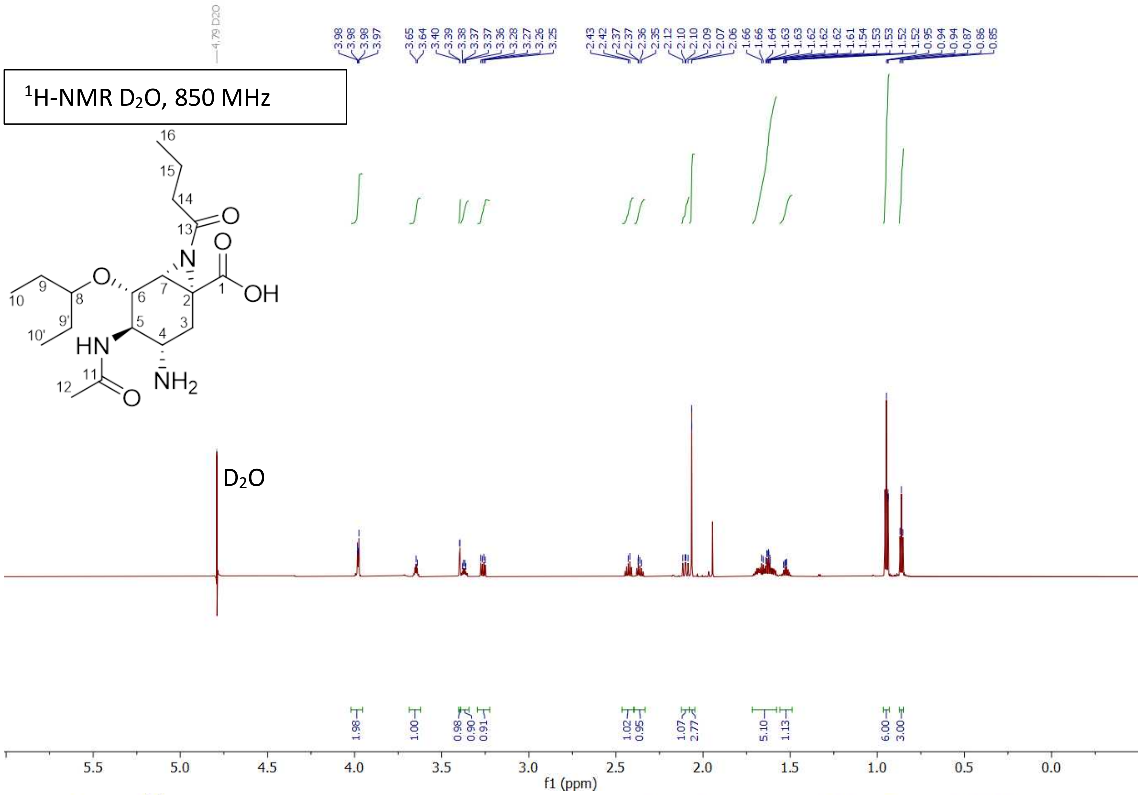

1H-NMR D2O, 850 MHz
4.79 D2O
3.98 3.98 3.98 3.97 3.65 3.64 3.40 3.39 3.38 3.37 3.37 3.36 3.28 3.27 3.26 3.25 2.43 2.42 2.37 2.37 2.36 2.35 2.12 2.10 2.10 2.09 2.07 2.06 1.66 1.66 1.64 1.63 1.63 1.62 1.62 1.61 1.54 1.53 1.53 1.52 1.52 0.95 0.94 0.94 0.87 0.86 0.85
16
15
14
13
O
N
O
OH
1
2
3
4
5
6
7
8
9
10
9'
10'
HN
11
12
O
NH2
D2O
1.98 1.00 0.98 0.90 0.91 1.02 0.95 1.07 2.77 5.10 1.13 6.00 3.00
5.5 5.0 4.5 4.0 3.5 3.0 2.5 2.0 1.5 1.0 0.5 0.0
f1 (ppm)

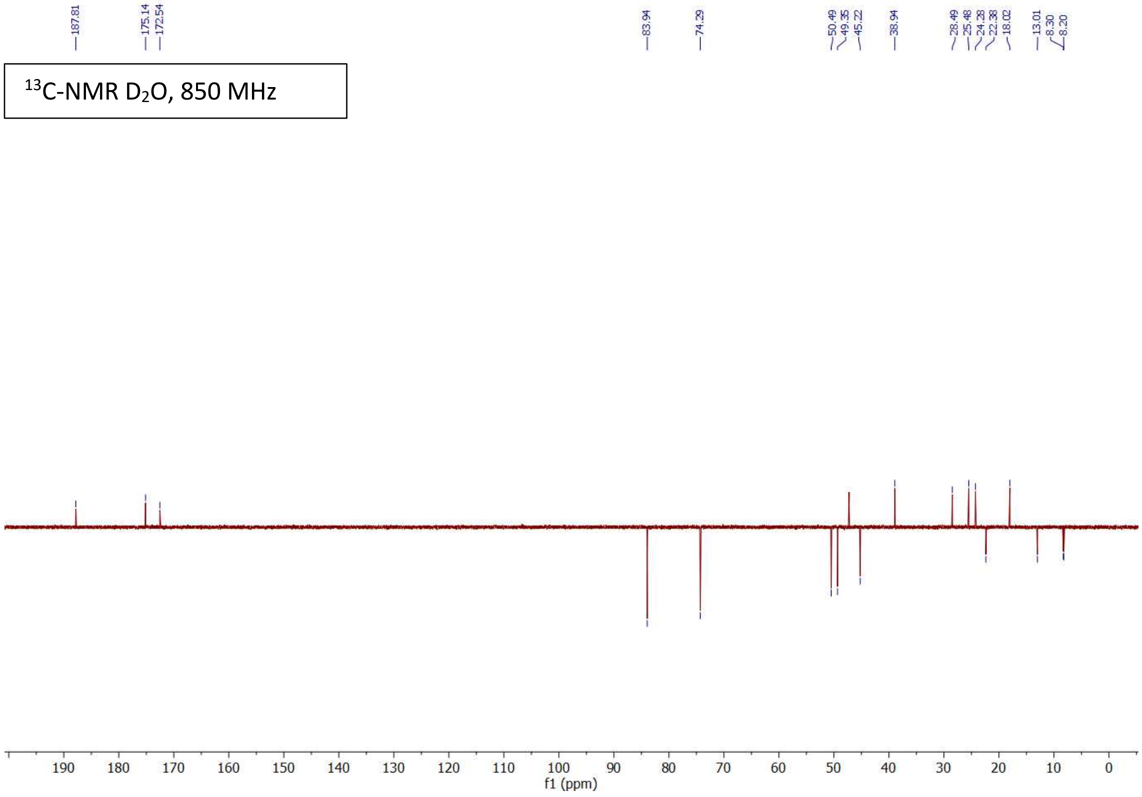

13C-NMR D2O, 850 MHz
187.81 175.14 172.54 83.94 74.29 50.49 49.35 45.22 38.94 28.49 25.48 24.28 22.38 18.02 13.01 8.30 8.20
190 180 170 160 150 140 130 120 110 100 90 80 70 60 50 40 30 20 10 0
f1 (ppm)

H-H COSY-NMR D_2O, 850 MHz

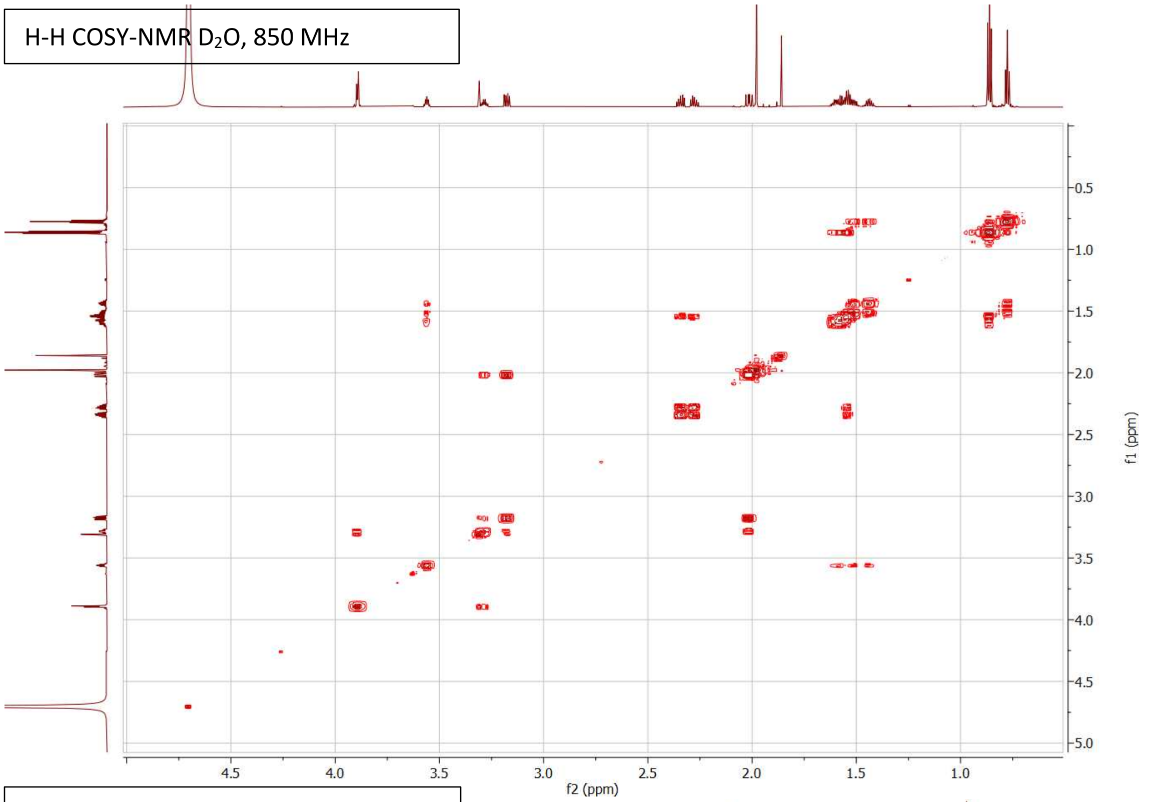

H-C HSQC-NMR D_2O, 850 MHz

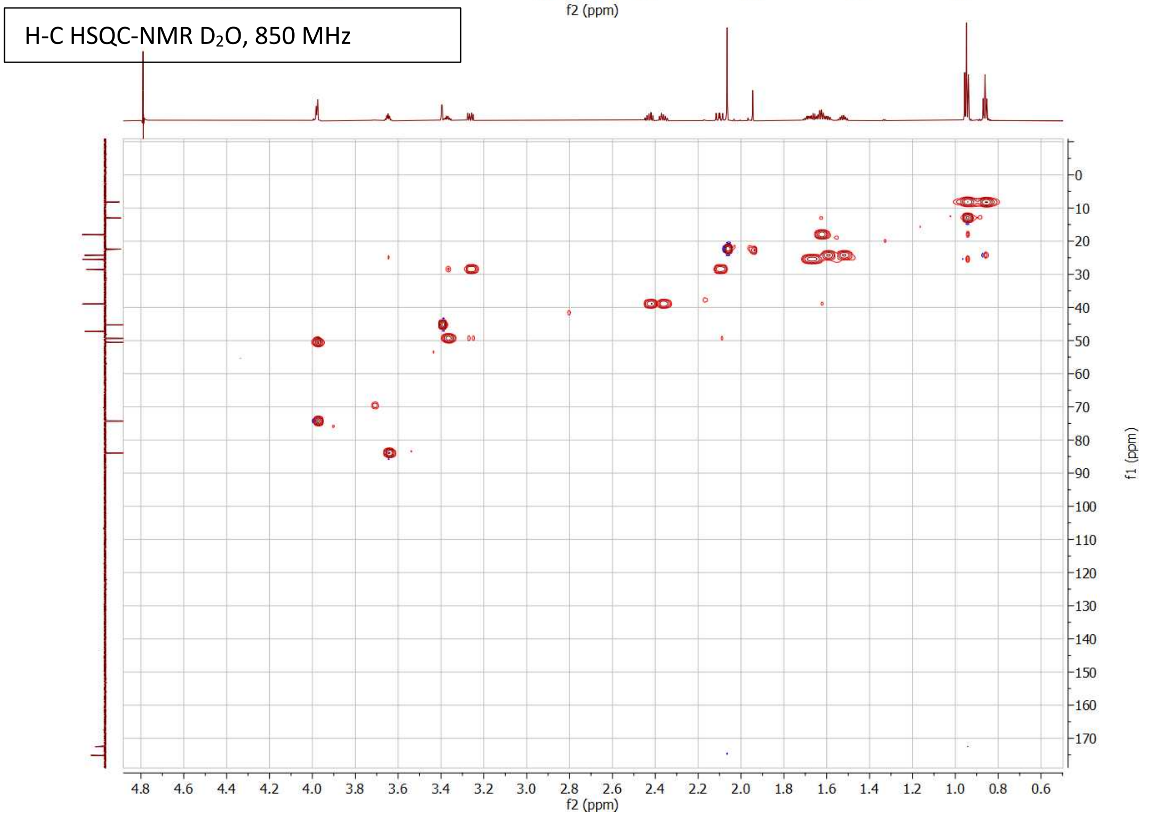

Supplementary References

1. P. J. Collins *et al.*, Crystal structures of oseltamivir-resistant influenza virus neuraminidase mutants. *Nature* **453**, 1258-1261 (2008).

2. R. Anandakrishnan, B. Aguilar, A. V. Onufriev, H++ 3.0: automating pK prediction and the preparation of biomolecular structures for atomistic molecular modeling and simulations. *Nucleic Acids Research* **40**, W537-W541 (2012).

3. D. A. Case *et al.*, AmberTools. *Journal of Chemical Information and Modeling* **63**, 6183-6191 (2023).

4. J. A. Maier *et al.*, ff14SB: Improving the Accuracy of Protein Side Chain and Backbone Parameters from ff99SB. *Journal of Chemical Theory and Computation* **11**, 3696-3713 (2015).

5. W. L. Jorgensen, J. Chandrasekhar, J. D. Madura, R. W. Impey, M. L. Klein, Comparison of simple potential functions for simulating liquid water. *The Journal of Chemical Physics* **79**, 926-935 (1983).

6. J. Wang, R. M. Wolf, J. W. Caldwell, P. A. Kollman, D. A. Case, Development and testing of a general amber force field. *Journal of Computational Chemistry* **25**, 1157-1174 (2004).

7. J. Wang, W. Wang, P. A. Kollman, D. A. Case, Automatic atom type and bond type perception in molecular mechanical calculations. *Journal of Molecular Graphics and Modelling* **25**, 247-260 (2006).

8. M. J. T. Frisch, G. W.; Schlegel, H. B.; Scuseria, G. E.; Robb, M. A.; Cheeseman, J. R.; Scalmani, G.; Barone, V.; Petersson, G. A.; Nakatsuji, H.; Li, X.; Caricato, M.; Marenich, A. V.; Bloino, J.; Janesko, B. G.; Gomperts, R.; Mennucci, B.; Hratchian, H. P.; Ortiz, J. V.; Izmaylov, A. F.; Sonnenberg, J. L.; Williams-Young, D.; Ding, F.; Lipparini, F.; Egidi, F.; Goings, J.; Peng, B.; Petrone, A.; Henderson, T.; Ranasinghe, D.; Zakrzewski, V. G.; Gao, J.; Rega, N.; Zheng, G.; Liang, W.; Hada, M.; Ehara, M.; Toyota, K.; Fukuda, R.; Hasegawa, J.; Ishida, M.; Nakajima, T.; Honda, Y.; Kitao, O.; Nakai, H.; Vreven, T.; Throssell, K.; Montgomery, J. A., Jr.; Peralta, J. E.; Ogliaro, F.; Bearpark, M. J.; Heyd, J. J.; Brothers, E. N.; Kudin, K. N.; Staroverov, V. N.; Keith, T. A.; Kobayashi, R.; Normand, J.; Raghavachari, K.; Rendell, A. P.; Burant, J. C.; Iyengar, S. S.; Tomasi, J.; Cossi, M.; Millam, J. M.; Klene, M.; Adamo, C.; Cammi, R.; Ochterski, J. W.; Martin, R. L.; Morokuma, K.; Farkas, O.; Foresman, J. B.; Fox, D. J. (2016) Gaussian 16. (Gaussian, Inc., Wallingford CT).

9. D. A. Case *et al.*, *Amber 2021* (University of California, San Francisco, 2021).

10. W. Humphrey, A. Dalke, K. Schulten, VMD: Visual molecular dynamics. *Journal of Molecular Graphics* **14**, 33-38 (1996).

11. D. R. Roe, T. E. Cheatham, III, PTRAJ and CPPTRAJ: Software for Processing and Analysis of Molecular Dynamics Trajectory Data. *Journal of Chemical Theory and Computation* **9**, 3084-3095 (2013).

12. T. D. Kühne *et al.*, CP2K: An electronic structure and molecular dynamics software package-Quickstep: Efficient and accurate electronic structure calculations. *The Journal of Chemical Physics* **152** (2020).

13. J. P. Perdew, K. Burke, M. Ernzerhof, Generalized gradient approximation made simple. *Physical review letters* **77**, 3865 (1996).

14. S. Goedecker, M. Teter, J. Hutter, Separable dual-space Gaussian pseudopotentials. *Physical Review B* **54**, 1703 (1996).

15. G. A. Tribello, M. Bonomi, D. Branduardi, C. Camilloni, G. Bussi, PLUMED 2: New feathers for an old bird. *Computer Physics Communications* **185**, 604-613 (2014).

16. M. Invernizzi, M. Parrinello, Exploration vs convergence speed in adaptive-bias enhanced sampling. *Journal of Chemical Theory and Computation* **18**, 3988-3996 (2022).

17. M. Marianski, A. Supady, T. Ingram, M. Schneider, C. Baldauf, Assessing the Accuracy of Across-the-Scale Methods for Predicting Carbohydrate Conformational Energies for the Examples of Glucose and α-Maltose. *Journal of Chemical Theory and Computation* **12**, 6157-6168 (2016).

18. D. Cremer, J. A. Pople, General definition of ring puckering coordinates. *Journal of the American Chemical Society* **97**, 1354-1358 (1975).

19. A. Ardèvol, C. Rovira, Reaction Mechanisms in Carbohydrate-Active Enzymes: Glycoside Hydrolases and Glycosyltransferases. Insights from ab Initio Quantum Mechanics/Molecular Mechanics Dynamic Simulations. *Journal of the American Chemical Society* **137**, 7528-7547 (2015).

20. J. Iglesias-Fernández, L. Raich, A. Ardèvol, C. Rovira, The complete conformational free energy landscape of β-xylose reveals a two-fold catalytic itinerary for β-xylanases. *Chemical Science* **6**, 1167-1177 (2015).

21. P. Hohenberg, W. Kohn, Inhomogeneous Electron Gas. *Physical Review* **136**, B864-B871 (1964).

22. F. Neese, Software Update: The ORCA Program System—Version 6.0. *WIREs Computational Molecular Science* **15**, e70019 (2025).

23. C. Adamo, V. Barone, Toward reliable density functional methods without adjustable parameters: The PBE0 model. *The Journal of Chemical Physics* **110**, 6158-6170 (1999).

24. F. Weigend, R. Ahlrichs, Balanced basis sets of split valence, triple zeta valence and quadruple zeta valence quality for H to Rn: Design and assessment of accuracy. *Physical Chemistry Chemical Physics* **7**, 3297-3305 (2005).

25. S. Grimme, J. Antony, S. Ehrlich, H. Krieg, A consistent and accurate ab initio parametrization of density functional dispersion correction (DFT-D) for the 94 elements H-Pu. *The Journal of Chemical Physics* **132**, 154104 (2010).

26. S. Grimme, S. Ehrlich, L. Goerigk, Effect of the damping function in dispersion corrected density functional theory. *Journal of Computational Chemistry* **32**, 1456-1465 (2011).

27. V. Ásgeirsson *et al.*, Nudged Elastic Band Method for Molecular Reactions Using Energy-Weighted Springs Combined with Eigenvector Following. *Journal of Chemical Theory and Computation* **17**, 4929-4945 (2021).

28. G. Mills, H. Jónsson, G. K. Schenter, Reversible work transition state theory: application to dissociative adsorption of hydrogen. *Surface Science* **324**, 305-337 (1995).

29. C. A. Mooney *et al.*, Oseltamivir Analogues Bearing N-Substituted Guanidines as Potent Neuraminidase Inhibitors. *Journal of Medicinal Chemistry* **57**, 3154-3160 (2014).

30. D. Ellis *et al.*, Structure-based design of stabilized recombinant influenza neuraminidase tetramers. *Nature Communications* **13**, 1825 (2022).

31. S. Q. Zheng *et al.*, MotionCor2: anisotropic correction of beam-induced motion for improved cryo-electron microscopy. *Nature Methods* **14**, 331-332 (2017).

32. A. Rohou, N. Grigorieff, CTFFIND4: Fast and accurate defocus estimation from electron micrographs. *J Struct Biol* **192**, 216-221 (2015).

33. J. Zivanov *et al.*, New tools for automated high-resolution cryo-EM structure determination in RELION-3. *eLife* **7**, e42166 (2018).

34. A. Burt *et al.*, An image processing pipeline for electron cryo-tomography in RELION-5. *bioRxiv* 10.1101/2024.04.26.591129, 2024.2004.2026.591129 (2024).

35. J. Zivanov, T. Nakane, S. H. W. Scheres, A Bayesian approach to beam-induced motion correction in cryo-EM single-particle analysis. *IUCrJ* **6**, 5-17 (2019).

36. E. F. Pettersen *et al.*, UCSF Chimera—A visualization system for exploratory research and analysis. *J. Comput. Chem* **25**, 1605-1612 (2004).

37. C. J. Vavricka *et al.*, Structural and Functional Analysis of Laninamivir and its Octanoate Prodrug Reveals Group Specific Mechanisms for Influenza NA Inhibition. *PLOS Pathogens* **7**, e1002249 (2011).

38. P. Emsley, B. Lohkamp, W. G. Scott, K. Cowtan, Features and development of Coot. *Acta Crystallogr., Sect. D: Struct. Biol.* **66**, 486 (2010).

39. D. Liebschner *et al.*, Macromolecular structure determination using X-rays, neutrons and electrons: recent developments in Phenix. *Acta Crystallogr., Sect. D: Struct. Biol.* **75**, 861 (2019).

40. Y. Lin, Y. Gu, J. W. McCauley, Optimization of a Quantitative Micro-neutralization Assay. *JoVE* doi:10.3791/54897, e54897 (2016).

41. M. Matrosovich, T. Matrosovich, W. Garten, H. D. Klenk, New low-viscosity overlay medium for viral plaque assays. *Virol J* **3**, 63 (2006).
